# Understanding the risk of developing weight-related complications associated with different body mass index categories: a systematic review

**DOI:** 10.1186/s13098-022-00952-4

**Published:** 2022-12-07

**Authors:** Adam Ben Taieb, Erika Roberts, Maria Luckevich, Sara Larsen, Carel W. le Roux, Paulo Gomes de Freitas, Dingeman Wolfert

**Affiliations:** 1 Wickenstones Ltd., Oxford, UK; 2 Novo Nordisk Canada Inc., Mississauga, ON Canada; 3 Novo Nordisk A/S, Søborg, Denmark; 4 Diabetes Complications Research Centre, Conway Institute, University College, Dublin, Ireland

**Keywords:** Obesity, Complication, Risk equation, Body mass index, Cardiovascular disease, Type 2 diabetes, Stroke, Risk assessment, Overweight, Comorbidity

## Abstract

**Background:**

Obesity and overweight are major risk factors for several chronic diseases. There is limited systematic evaluation of risk equations that predict the likelihood of developing an obesity or overweight associated complication. Predicting future risk is essential for health economic modelling. Availability of future treatments rests upon a model’s ability to inform clinical and decision-making bodies.

This systematic literature review aimed to identify studies reporting (1) equations that calculate the risk for individuals with obesity, or overweight with a weight-related complication (OWRC), of developing additional complications, namely T2D, cardiovascular (CV) disease (CVD), acute coronary syndrome, stroke, musculoskeletal disorders, knee replacement/arthroplasty, or obstructive sleep apnea; (2) absolute or proportional risk for individuals with severe obesity, obesity or OWRC developing T2D, a CV event or mortality from knee surgery, stroke, or an acute CV event.

**Methods:**

Databases (MEDLINE and Embase) were searched for English language reports of population-based cohort analyses or large-scale studies in Australia, Canada, Europe, the UK, and the USA between January 1, 2011, and March 29, 2021. Included reports were quality assessed using an adapted version of the Newcastle Ottawa Scale.

**Results:**

Of the 60 included studies, the majority used European cohorts. Twenty-nine reported a risk prediction equation for developing an additional complication. The most common risk prediction equations were logistic regression models that did not differentiate between body mass index (BMI) groups (particularly above 40 kg/m^2^) and lacked external validation. The remaining included studies (31 studies) reported the absolute or proportional risk of mortality (29 studies), or the risk of developing T2D in a population with obesity and with prediabetes or normal glucose tolerance (NGT) (three studies), or a CV event in populations with severe obesity with NGT or T2D (three studies). Most reported proportional risk, predominantly a hazard ratio.

**Conclusion:**

More work is needed to develop and validate these risk equations, specifically in non-European cohorts and that distinguish between BMI class II and III obesity. New data or adjustment of the current risk equations by calibration would allow for more accurate decision making at an individual and population level.

**Supplementary Information:**

The online version contains supplementary material available at 10.1186/s13098-022-00952-4.

## Background

Obesity is a disease in its own right but also a major risk factor for several chronic diseases, including type 2 diabetes (T2D), cardiovascular (CV) disease (CVD), particularly heart disease and stroke, musculoskeletal disorders, fatty liver disease, hypertension, and some cancers [1]. Obesity is therefore strongly associated with increased risk of morbidity and mortality in affected individuals. Globally, obesity prevalence is rising with a close to tripling of the worldwide prevalence between 1975 and 2016, bringing the number of adults with overweight to 1.9 billion, of whom over 650 million had obesity [2].

Obesity and overweight are defined as abnormal or excessive fat accumulation causing a deterioration in health [3–5]. Epidemiologically, obesity is also classified by body mass index (BMI), a simple index derived from weight (kg) and height (m^2^). A BMI between 25 and 29.9 kg/m^2^ is classified as overweight and obesity as a BMI greater than or equal to 30 kg/m^2^, further subdivided into class I (30.0–34.9 kg/m^2^) and class II (35.0–39.9 kg/m^2^) [2]. A BMI greater than or equal to 40 kg/m^2^ is referred to as class III obesity.

Furthermore, obesity imposes an increasing economic burden on society and exacerbates the costs of both obesity-related and unrelated comorbidities [6]. This is especially true of class III obesity which has a disproportionately large burden on the healthcare system [7], and in 2013 the healthcare burden of individuals with a BMI above 35 kg/m^2^ was 60% of total obesity-related costs [8]. By 2030, obesity above a BMI of 35 kg/m^2^ is predicted to rise to one in four adults in the USA, becoming the leading BMI category for certain populations, including women, non-Hispanic Black adults, and low-income adults [8, 9]. Direct healthcare and indirect economic cost burden are forecast to be upwards of tens of billions of dollars per year by 2030 [10].

Overweight, a precursor to obesity, significantly increases the risk of developing many complications also related to obesity [11]. The overweight and obesity class I–III cut-offs were developed in Western populations but vary depending on ethnicity [12], for example the original class I, BMI 30–34.9 kg/m^2^, has been lowered for Asian and South Asian populations to BMI 27.5 kg/m^2^ or 25 kg/m^2^ [13, 14]. To consider the health consequences of overweight while potentially distinguishing them from those that are not the result of excess adipose tissue, the patient population with a BMI of 25–29.9 kg/m^2^ was included in this systematic literature review (SLR) population criteria when a weight-related complication was present.

Very few analyses of populations with class III obesity exist, although a BMI of 40–59 kg/m^2^ is associated with substantially elevated rates of total mortality due to obesity complications [15]. The health and economic burden caused by increasing BMI have resulted in adiposity being included among the World Health Organization’s (WHO) global action plan for the prevention and control of non-communicable diseases. This action plan includes a specific objective to halt the rise in obesity prevalence at its 2010 level by 2025 [16, 17]. Controlling the growth of populations with obesity is especially relevant during global health emergencies, for example the coronavirus disease 2019 pandemic, where obesity complications are a major risk factor for survival [18, 19].

Risk equations can be developed to predict the likelihood of a person with or without a pre-existing condition of developing an associated obesity complication. These equations can help decision making at the patient and health management level. Informative risk equation outcomes hinge on choosing relevant risk predictors. Risk predictors can be lifestyle based (e.g. smoking, and exercise), socio-economic (e.g. income, and education), phenotypic (e.g. BMI, and sex), endotypic (e.g. hypertension, and insulin sensitivity), as well as health and disease related (e.g. diabetes, and heart attack). Risk predictor usage varies between equations and BMI’s association with the aforementioned diseases make it an obvious risk predictor choice to determine health outcome risk.

Greater understanding is required as to how risk equations can be used to reliably predict individual and population-based risk [20–22]. For example, in order to assess the risk of obesity-associated chronic diseases (such as CVD and T2D), multiple risk equations have been developed, such as the Cambridge Risk Score, QDiabetes, Finnish Diabetes Risk Score (FINDRISC), Leicester Risk Assessment, QD Score, (T2D) Framingham, and QRISK2 (CVD). Yet they lack sensitivity or validation for individuals with high BMI levels, although health outcomes are differentially impacted dependent on the specific BMI level [23, 24].

Weight related risk prediction equations that account for the statistical interaction between BMI levels and other risk predictors with the risk of outcome are of high value, without which risks associated with certain populations will not be accurately predicted in models. In the absence of sensitivity or validation for individuals with high BMI levels, a calibration (to estimate uncertain parameters and more accurately defining model uncertainty, by comparing model outputs with empirical data) of risk equations using current risk estimates from the literature (such as of the occurrence of CVD and development of T2D in high BMI individuals) may lend precision to estimates of health outcomes. We sought to identify reliable risk prediction usage that distinguishes between, and were robust for, all BMI levels.

While some studies have sought to predict the risk of weight-related mortality or complication development using risk equations, there has been limited systematic evaluation of these equations. This SLR had two overarching aims, firstly to identify published risk equations for developing obesity and overweight with a weight-related complication (OWRC) associated complications. Second, to identify studies that calculated absolute or proportional risk of individuals with OWRC, obesity, or severe obesity developing specific disease outcomes (e.g., T2D, CV event, or death). This insight will aid economic modelers in developing more accurate predictions of weight-related disease outcomes, which will in turn aid decision makers in evaluating measures that promise to improve health outcomes and reduce the social and economic burden in these populations.

## Methods

A systematic review was undertaken to identify all studies that provide information on the risk associated with people living with obesity or OWRC developing complications. This review was conducted according to the York Centre for Reviews and Dissemination guidelines for conducting reviews [25] and is reported in accordance with the Preferred Reporting Items for Systematic Reviews and Meta-Analyses (PRISMA) statement [26, 27]. The protocol was pre-registered on the PROSPERO register of systematic reviews (CRD42021245324).

### Study identification

The following electronic databases were searched as standard evidence sources: MEDLINE, MEDLINE In-Process and e-publications ahead-of-print (via PubMed), Embase (via Embase.com), the technology appraisal submissions to the National Institute for Clinical Excellence (NICE) using risk equations, and bibliographic reference lists from included studies.

Searches were conducted on March 25, 2021. All peer-reviewed articles or errata published in the past 10 years (January 2011–March 2021) in Australia, Canada, Europe, the UK, and the USA were included. Only English language studies were included, full inclusion and exclusion criteria are reported in Additional file 1: Table S1. The search strings can be found in Additional file 1: Tables S2 (Embase) and Additional file 1: Table S3 (PubMed) (see Additional file 1).

Included studies reported on adults of ≥ 18 years with a BMI ≥ 30 kg/m^2^ or BMI ≥ 25 kg/m^2^ with the presence of at least one weight-related complication. There were no restrictions for interventions or comparators. Studies reported at least one of the following four outcomes: (1) the risk equation which calculated the risk of developing at least one additional complication: prediabetes, T2D, CVD, heart disease (including heart failure with preserved ejection fraction), acute coronary syndrome (unstable angina or myocardial infarction [MI]), stroke, musculoskeletal disorders, knee replacement/arthroplasty, obstructive sleep apnea in a population with obesity or OWRC; (2) absolute risk or proportional risk of developing T2D in a population with obesity and prediabetes or normal glucose tolerance (NGT); (3) absolute risk or proportional risk of having a CV event in a population with class III obesity and NGT or T2D; (4) absolute risk or proportional risk of mortality from knee surgery, stroke, or acute CV events in a population with obesity or OWRC. For outcomes 2–4: complications are T2D, hypertension, dyslipidemia, obstructive sleep apnea, or CVD; absolute risk refers to risk, rate, odds, and hazard outcomes; proportional risk refers to risk ratio, odds ratio, hazard ratio, relative risk, and risk reduction.

Study selection was conducted independently in duplicate. Disagreement between the reviewers was reviewed by a senior researcher (PGF), who made the final decision on inclusion or exclusion of the record. Quality appraisal and data extraction (extraction categories in Additional file 1: Table S4) were undertaken by one reviewer (ABT) and checked by a second (ER).

A thematic analysis of the evidence was completed, and results are described in a narrative summary of the evidence. Outcome data was grouped where possible to enable descriptive analysis, e.g., patient characteristics and comorbidities. Heterogeneity observed between studies, in terms of methodology and the use of definitions or descriptors, was recorded and accounted for.

### Quality assessment

Studies were quality assessed based on three categories outlined by the Newcastle Ottawa Scale (NOS) [28] (with modification; categories were selection, comparability, outcome), as well as an additional category (risk equation validation) (see Additional file 1: Table S5). Between zero and fifteen stars were allocated to each study (a maximum of eight for the selection category, two for the comparability category, three for the outcome category, two for the validation category). Studies were ranked as high (≥ 13 stars), medium (11–12 stars), or low quality (≤ 10 stars).

## Results

### Study selection

**Fig. 1:**
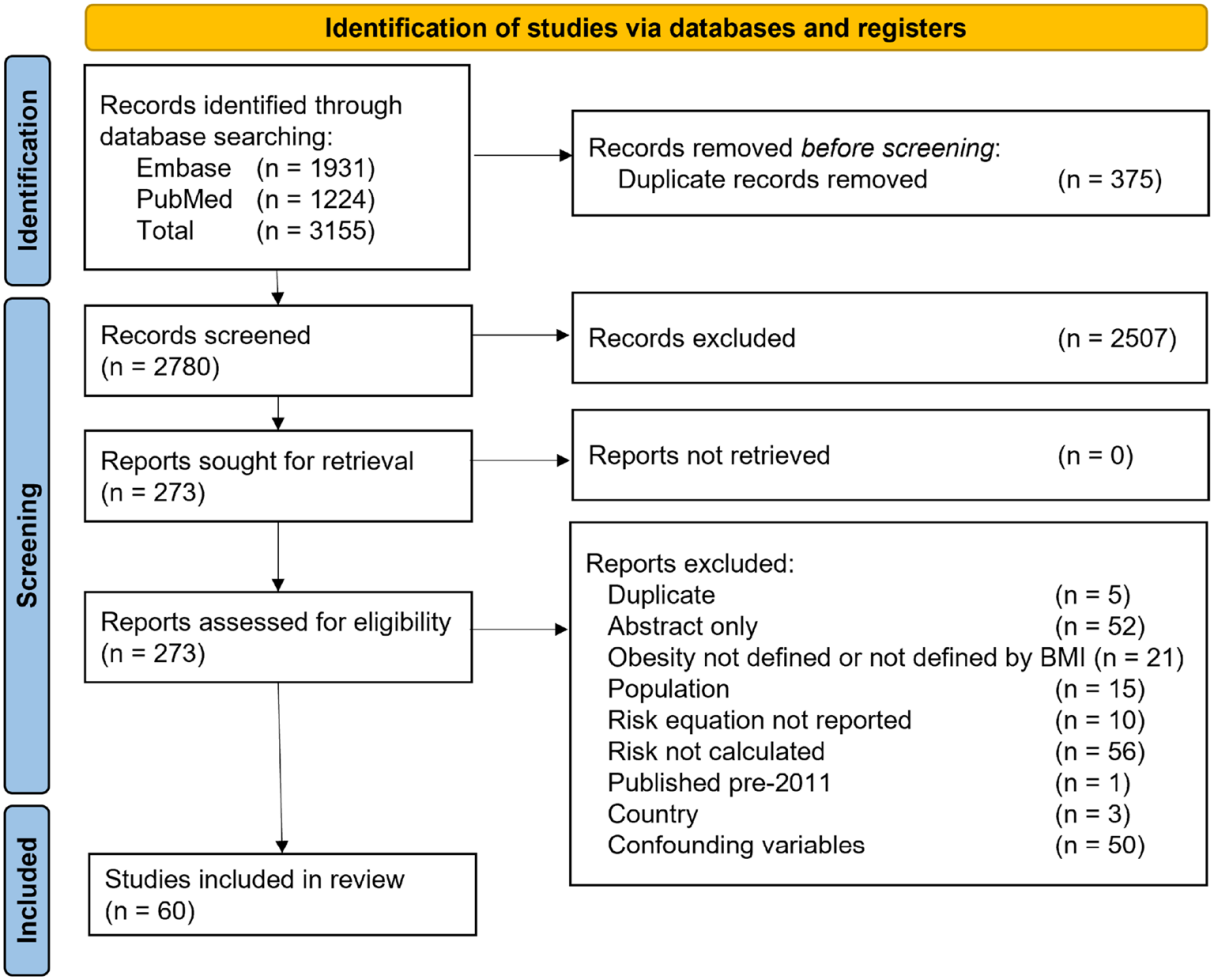
PRISMA flow diagram of study selection process. *BMI* body mass index, *PRISMA* Preferred Reporting Items for Systematic Reviews and Meta-Analysis

The details of the study selection are presented in a PRISMA flow diagram in Fig. 1.

A total of 3155 potentially relevant studies were identified for review. A de-duplication step to remove overlapping studies resulted in 375 exclusions. A further 2507 were excluded at the primary screening stage, based on their titles and/or abstracts, as they were not of relevance to the research question. These studies were excluded for reasons such as using a study sample from a small region from which generalising would not be robust, investigating other diseases, or being reviews or editorials.

During the evaluation of the full-text articles 213 were excluded for reasons such as being abstract only (52), having confounding variables (50), having incorrect population criteria (15), not measuring BMI or not defining obesity, or investigating other diseases (21), and can be found in Additional file 1: Table S6. Therefore, a total of 60 citations were included for this review (see Additional file 1: Table S7).

Of the 60 included studies, 29 reported risk equations, three reported risk of T2D, three reported the risk of a CV event, and 28 the risk of mortality. All were cohort studies or used data from cohort studies, most (49) were large (> 2000 participants) and 11 were small (< 2000 participants).

### Studies reporting risk equations

Twenty-nine included studies reported an equation to calculate the risk of people with obesity or OWRC developing at least one additional complication. Of these, 12 reported equations calculating the risk of T2D [23, 29–39], eight of CVD [29, 38, 40–45], five of stroke [46–50], two of obstructive sleep apnea [51, 52], MI [49, 53], and knee osteoarthritis or replacement [54, 55], and one of heart failure [56]. Outcomes were measured and defined in various ways, with some uniformity amongst complications. For example, of studies reporting a T2D risk equation, four used clinical medical diagnosis or diabetic medication use [29, 31, 37, 38], three WHO [2] diagnostic criteria [34, 36, 39], one the American Diabetes Association criteria [33], one a UK Read Code [35], and one patient-reported [32], while one did not report a definition [23] and another used multiple definitions, including medical diagnosis and WHO diagnostic criteria [30]. Of studies reporting a CV risk equation, five used relevant International Classification of Disease codes [29, 38, 40, 42, 45], two medical records/patient surveys [41, 43], and one did not report a definition [44].

The most common risk equations reported were logistic regression models [32, 38, 39, 49, 50, 52, 55], followed by a Cox proportional hazard model [30, 36, 40, 46, 53, 54], Cox regression model [33, 42, 45, 47, 56], survival model [31, 34], QRISK2 [41, 44], and FINDRISC [23, 37] (see Table 1 for full list). Most of the equations calculated the relative risk as a hazard ratio [30, 33, 36, 40, 42, 45–48, 53, 54, 56] using a Cox proportional hazard model also referred to as a Cox regression model. Other expressions of risk included odds ratios [29, 32, 49, 50, 52, 55] (all but one of which were logistic regression models), risk scores [33, 35, 41, 43, 51], regression coefficients [29, 31], and absolute risk, e.g., percentage [23, 34, 37–39, 44].

**Table 1:** Included studies reporting equations for predicting overweight/obesity-associated comorbidities

Study	Year	Country	Risk equation	Risk of developing	Risk predictors in the equation
Ahlin et al. [51]	2019	Italy	Population-based risk score	Obstructive sleep apnea	BMI, T2D, hypertension, age, sex, fasting glucose, aspartate transaminase, alanine transaminase, hepatic steatosis
Alssema et al. [29]	2012	Netherlands	Single risk stratification tool	T2D, CVD (and/or chronic kidney disease^a^)	BMI, hypertension^b^, age, sex, waist circumference, smoking, family history of MI or stroke, family history of diabetes
Apold et al. [54]	2014	Norway	Cox proportional hazard model	Knee replacement	BMI, height, age, sex, physical activity, smoking
Borgeraas et al. [53]	2014	Norway	Cox proportional hazard model	Acute myocardial infarction	BMI, diabetes, age, gender, left ventricular ejection fraction, smoking, ACE inhibitors, loop diuretics, pulmonary disease, systolic/diastolic blood pressure, previous acute myocardial infarction, coronary artery disease, serum creatinine levels, serum C-reactive protein, cholesterol, vitamin B6 or folate/B12, intervention status
Booth et al. [30]	2014	UK	Cox proportional hazard model	T2D	BMI, hypertension^b^, coronary heart disease, age, sex, HbA_1c_, index year, stroke, previous diagnosis of depression, smoking, total cholesterol, blood pressure, statin use
Bruce [40]	2015	USA	Cox proportional hazard model	CVD	BMI, diabetes, age, race, education, smoking, alcohol use, statin use, aspirin use, beta-blocker use
Burns et al. [46]	2019	UK	Cox proportional hazard model	Cerebrovascular event	BMI, diabetes, hypertension, age, sex, smoking, previous MI, chronic pulmonary disease, previous cerebrovascular event, peripheral vascular disease, coronary artery disease, left ventricular ejection fraction, redo-sternotomy, mitral valve replacement, perioperative return to the operating room (non-infectious), postoperative hemodialysis, postoperative cerebrovascular event, deep surgical wound infection
Chang et al. [41]	2016	UK	QRISK2	CVD	BMI, systolic blood pressure, total cholesterol/high-density lipoprotein ratio, smoking, vascular disease (atrial fibrillation, chronic kidney disease, hypertension, diabetes), age, sex, ethnicity, family history of premature coronary artery disease, deprivation
Coles et al. [31]	2020	UK	Survival model	T2D	BMI, sleep apnea, hypertension^b^, CVD, age, sex, ethnicity, deprivation, family history of diabetes, polycystic ovary syndrome, schizophrenia or bipolar affective disorder, depression, learning disabilities, renal/kidney disease, gestational diabetes, statin use, corticosteroid use, aspirin use, second generation ‘atypical’ antipsychotics, HbA_1c_, pulse rate, serum cholesterol, systolic/diastolic blood pressure, liver function test, waist circumference, smoking, alcohol use
Costanzo et al. [42]	2015	UK	Cox regression model	CVD	BMI, age, sex, duration of diabetes, smoking, systolic blood pressure, chronic obstructive pulmonary disorder, cancer, chronic kidney disease, previous CVD
de Boer et al. [43]	2015	Netherlands	SCORE-NL	CVD	BMI, diabetes, age, sex, systolic blood pressure, total cholesterol/high-density lipoprotein ratio, smoking, first-degree family history of CVD, physical activity, estimated glomerular filtration rate, poor metabolic control, albuminuria
Ding et al. [32]	2015	Australia	Logistic regression models	T2D	BMI, CVD, age, sex, country of birth, education, disadvantage, family history of high blood pressure, family history of heart disease, family history of T2D, high blood pressure, high cholesterol, smoking, alcohol use, physical activity, psychological distress, daily sitting and sleeping time
Erridge et al. [52]	2021	UK	Logistic regression model	Obstructive sleep apnea	BMI, hypertension, T2D, hyperlipidemia, age, sex, bariatric surgery, smoking, gastroesophageal reflux disease, chronic obstructive pulmonary disorder, chronic renal disease, hypothyroidism, acromegaly, craniofacial/upper airway anomalies, benzodiazepine use
Ferket et al. [47]	2014	USA and Netherlands	Cox regression model	Intracerebral hemorrhage, ischemic stroke	BMI, hypertension^b^, diabetes, coronary heart disease, age, sex, African American ethnicity, smoking, systolic/diastolic blood pressure, total cholesterol, high-density lipoprotein, waist-to-hip ratio, estimated glomerular filtration rate
Glogner et al. [56]	2014	Sweden	Cox regression model	Heart failure	BMI, T2D, CVD (or medication for MI, arterial fibrillation, or valve disease), hypertension^b^, age, sex, systolic/diastolic blood pressure, HbA_1c_, smoking, microalbuminuria
Gray et al. [44]	2014	UK	QRISK2, Framingham Lipids, Framingham BMI, Joint British Societies 2	CVD	BMI, hypertension^b^, age, systolic/diastolic blood pressure, sex, total cholesterol/high-density lipoprotein ratio, total cholesterol, triglycerides, family history of CVD, smoking, alcohol use, physical activity, psychological distress, daily sitting and sleeping time
Gray et al. [23]	2015	UK	Cambridge Risk Score, FINDRISC, Leicester Risk Assessment, QDiabetes	T2D	BMI, hypertension^b^, age, ethnicity, sex, family history of diabetes, smoking, steroid treatment, deprivation, physical activity, fruit and vegetable intake, history of high blood glucose
Guasch Ferré et al. [33]	2012	Spain	Cox regression model	T2D	BMI, hypertension, smoking, family history of T2D, alcohol use, fasting plasma glucose
Hippisley-Cox et al. [48]	2013	UK	QStroke	Stroke, transient ischemic attack	BMI, hypertension^b^, coronary heart disease, T2D, age, sex, ethnicity, smoking, atrial fibrillation, systolic blood pressure, total cholesterol/high-density lipoprotein ratio, family history of coronary disease, deprivation, rheumatoid arthritis, chronic renal disease, type 1 diabetes, congestive cardiac failure, valvular heart disease
Jackson et al. [49]	2012	USA	Logistic regression model	MI, stoke, cardiac arrest	BMI, hypertension, diabetes, age, sex, race, smoking, alcohol use, transient ischemic attack, stroke, hemiplegia, central nervous system tumor, MI, previous percutaneous coronary intervention, previous cardiac surgery, angina, previous surgery for peripheral vascular disease, ventilator, disseminated cancer, preoperative open/infected wound, chronic obstructive pulmonary disease, pneumonia, dyspepsia, esophageal varices, steroid use, weight loss, systemic inflammatory response syndrome/sepsis, dependent
Joshy et al. [45]	2014	Australia	Cox regression model	CVD	BMI, hypertension^b^, diabetes, age, sex, aspirin use, hypercholesterolemia, physical activity, smoking, alcohol use, annual income, education, region of residence, health insurance
Ligthart et al. [34]	2016	Netherlands	Survival model	T2D	BMI, prediabetes, glucose tolerance, age
Mathur et al. [35]	2012	UK	QDScore	T2D	BMI, hypertension^b^, CVD, age, sex, ethnicity, deprivation, family history of T2D, smoking, corticosteroid use, estimated glomerular filtrate
Mustafina et al. [36]	2021	Russia	Cox proportional hazard model	T2D	BMI, hypertension, dyslipidemia, CVD, age, sex, fasting plasma glucose, smoking, alcohol consumption, education, marital status, fruit and vegetable consumption, family history of T2D, physical activity
Phillips et al. [37]	2013	Ireland	Wilson, Balkau FINDRISC, Schulze Kahn Enhanced, Kahn Basic, Griffin	T2D	Varied (all include BMI)
Rauh et al. [38]	2018	Australia	Logistic regression model	T2D, CVD (and/or chronic kidney disease^a^)	BMI, hypertension^b^, age, sex, waist circumference, smoking, parent or sibling with MI or stroke, parent or sibling with diabetes
Wilkinson et al. [39]	2020	USA	Logistic regression model	T2D	BMI, hypertension, age, sex, race, systolic/diastolic blood pressure, high-density lipoproteins, triglycerides, waist circumference
Winter et al. [50]	2016	Germany	Logistic regression model	Stroke, transient ischemic attack	BMI, diabetes, dyslipidemia, arterial hypertension, age, sex, waist-to-hip ratio, waist-to-height ratio, physical activity, smoking
Zhang et al. [55]	2011	UK	Logistic regression model	knee osteoarthritis	BMI, age, gender, occupational risk, previous knee injury, family history of knee osteoarthritis

*ACE* angiotensin converting enzyme, *BMI* body mass index, *CVD* cardiovascular disease, *FINDRISC* Finnish Diabetes Risk Score, *HbA*_*1c*_ glycated hemoglobin, *MI* myocardial infarction, *SLR* systematic literature review, *T2D* type 2 diabetes

^a^Comorbidity not in SLR scope

^b^Antihypertensive use

### Risk equation building

All equations in the included studies were developed and/or validated using cohort data. These data ranged in terms of cohort size (161 [51] to 3.5 million [48] participants) and differed with regard to cohort populations used to validate the equation.

The time period over which risk was assessed ranged from 30 days [49] to lifetime [34]. The most reported (31%) risk time frame was 10 years [23, 31, 35, 37, 39, 43, 44, 47, 48], 48% of the risk equations were based on the time frame of the cohort follow-up period [30, 32, 33, 39–42, 45, 49, 52–56], and two did not report a time frame [46, 50].

In terms of geography, most risk equations were developed or validated with cohorts in Europe (UK [23, 30, 31, 35, 41, 42, 44, 46, 48, 52, 55], the Netherlands [29, 34, 43, 47], Norway [53, 54], Germany [50], Ireland [37], Italy [51], Russia [36], Spain [33], Sweden [56]), and the remaining on cohorts either in the USA [39, 40, 47, 49] or Australia [32, 38, 45]. One study used cohort data from two countries (the Netherlands and the USA) [47].

### Validation and equation comparisons

Validation of equation performance was carried out on single or multiple data sets from internal or external cohorts. Not all studies mentioned equation validation and only six studies validated their equations with external cohorts, which is considered the most accurate validation method [23, 35, 38, 41, 43, 44]. The equations were typically tested against more than one externally validated model.

### Risk predictors

**Fig. 2:**
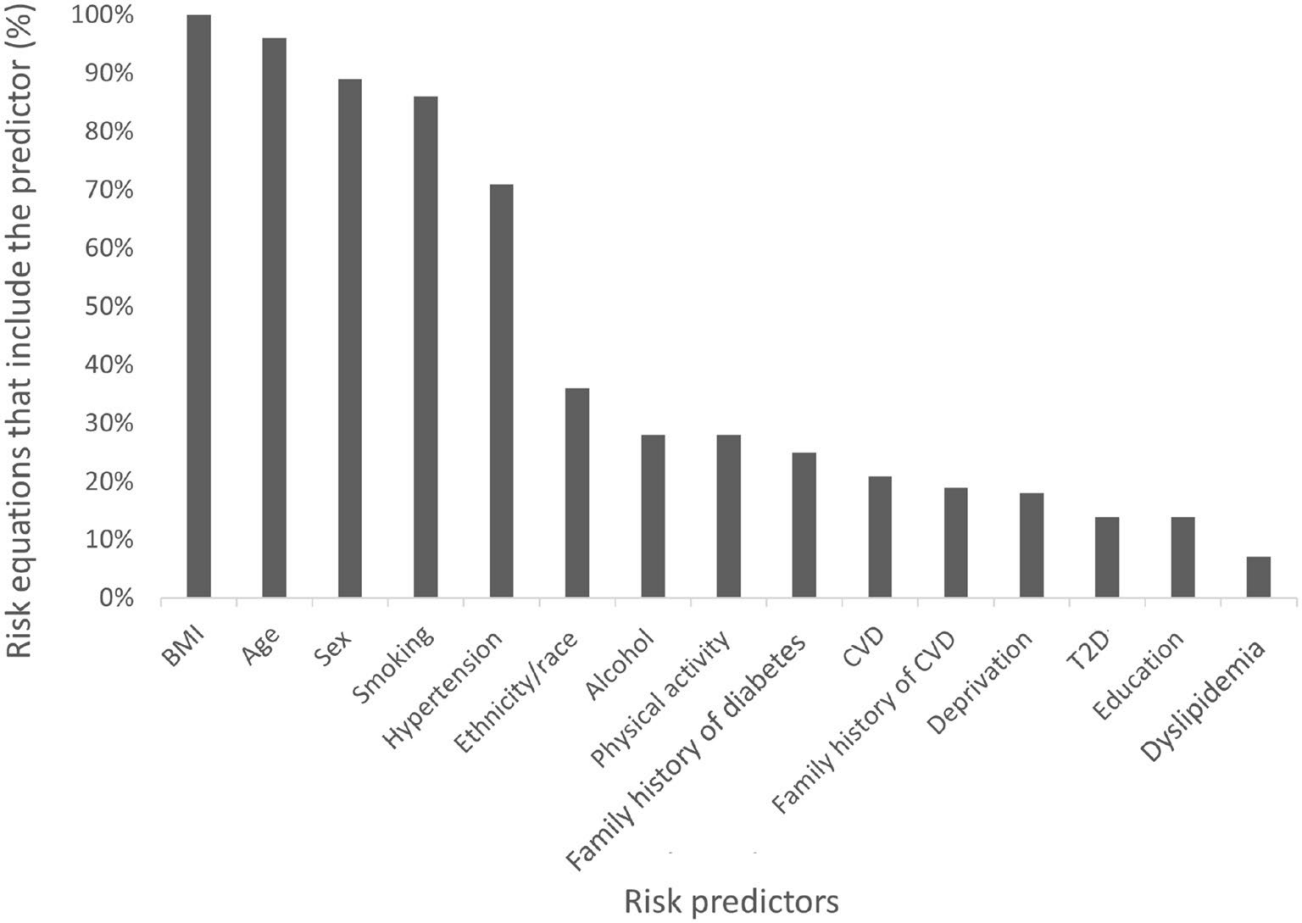
The most common risk predictors used in overweight and obesity risk equations. *BMI* body mass index, *CVD* cardiovascular disease, *T2D* type 2 diabetes

As the studies measured different outcomes, there was some variation in the risk predictors included. Even when calculating the risk of developing the same complication, risk predictors varied, which could have been due to differences in equations used, individual cohort data, or definitions of the disease outcomes measured (Fig. 2, and Table 1 for full list of variables and references).

In terms of population characteristics, after BMI, age was the most widely included across studies (93%). When individual background was taken into account, both deprivation (17% of studies) and education (14% of studies) were considered as well as income (3%) and family medical history (41%). Smoking was the major lifestyle risk predictor used in 83% of studies, followed by alcohol consumption (28%) and physical activity (28%). Predictors that could alleviate risk such as lifestyle intervention or pharmacotherapy were not included in any study and neither was the metabolic health of people with obesity.

### Risk predictor selection

The rationale behind why risk factors were selected for the equations varied. The majority reported on their rationale. Tools such as the Framingham or QRISK2 include risk predictors that were used in six studies [23, 35, 37, 38, 41, 44] and use of previously cited risk predictors or established clinical risk factors [29, 31, 39, 48, 55] was not uncommon. Some studies designed methods to identify risk predictors specific for their study, such as Jackson et al. who considered all cohort baseline characteristics and used a backwards step regression to select the equation risk predictors [49]. A minority of studies either did not discuss risk predictor selection or their chosen variables were assessed using the cohort data at baseline as risk predictors without detailed explanation [30, 32, 34, 40, 42, 43, 45–47, 50–52, 54, 56].

### BMI risk predictor

The use of BMI as a categorization of obesity levels was an inclusion criterion for this SLR, however, the range and granularity of how BMI was included into each equation differed. WHO defines weight according to six BMI ranges (underweight < 18 kg/m^2^, normal weight 18.5–24.9 kg/m2, overweight 25.0–29.9 kg/m^2^, obesity class I 30.0–34.9 kg/m^2^, obesity class II 35.0–39.9 kg/m^2^, obesity class III ≥ 40 kg/m^2^) yet only 13 studies used these categories [2, 29, 30, 32, 42–44, 48–50, 52–55], and only five studies included a breakdown of BMI above 30 kg/m^2^ [30, 42, 49, 52, 56]. Generally, most studies (18) did not report beyond BMI ≥ 30 kg/m^2^ in the baseline characteristics of the cohort used to develop or validate the risk equation or in the outcomes.

### Quality assessment

NOS scores for the included risk equation studies ranged between eight to 14 stars with nine assessed as high quality [31, 33, 35–37, 39, 48, 54, 56], eight as medium [23, 29, 41, 43, 44, 47, 49, 52], and 12 as low quality [30, 32, 34, 38, 40, 42, 45, 46, 50, 51, 53, 55]. For the validation component, six out of the 29 papers scored full marks and 13 received no stars (see Additional file 1: Table S8).

Two studies achieved the highest NOS score of 14, Hippisley-Cox et al. [48] and Phillips et al. [37].

Phillips et al. [37] compared seven diabetes risk assessment tools, on the same cohort of 2047 adults, aged 50–69, with a BMI range of 28.3–32.3. Their main findings were that most diabetes risk scores offer limited ability to identify subjects with metabolic abnormalities and at risk of developing T2D. Estimates varied greatly, even when comparing risk scores that were based on the same factors (e.g. lifestyle – FINDRISC or clinical – Wilson risk scores) there was not complete concordance. The wide range of risk estimates between the risk scores were related to exclusion of certain factors, differential variable weighting, variation in high-risk thresholds, and differences in populations used to develop these scores. The study promoted the necessity to validate equations specifically for each population to identify, not just high-risk individuals, but disease hot spots that can be targeted by public health interventions.

Hippisley-Cox et al. [48] aimed to develop and validate QStroke, an algorithm to estimate risk of stroke or transient ischemic attack, in 3.5 million adults aged 25–84 years old. They compared QStroke to CHADS2 and CHA2DS2VASc in patients with atrial fibrillation, and they compared it to the Framingham stroke score in the stroke/transient ischemic attack free population. QStroke had improved performance compared against all three scores although levels of discrimination were lower for patients with atrial fibrillation. In atrial fibrillation patients there is a slight over prediction for women at high levels of predicted risk, whereas the model is well calibrated for men. They identified a specific population, those for whom anticoagulation is considered, that could particularly benefit from Qstroke. Qstroke incorporates weighted established risk factors, such as ethnicity and deprivation, that are absent from existing stroke risk tools. It is also more patient friendly, in terms of giving absolute measures of stroke risk and it is aimed at being incorporated into GP practices.

### Studies reporting absolute or proportional risk

#### Risk of T2D

Three studies reported the risk of developing T2D in a population with obesity (BMI ≥ 30 kg/m^2^) and NGT or prediabetes [34, 57, 58] and were carried out in the Netherlands [34], Poland [58], or the USA [57] (Table 2).

**Table 2:** Included studies reporting T2D risk in a population with obesity with NGT or prediabetes

Study	Year	Country	Glycemic status	BMI (kg/m^2^)	Risk	Note
Blume et al. [57]	2015	USA	NGT	30.0–34.9	HR 3.4	Relative to normal weight (BMI 18.5–25.0)
				35.0–39.9	HR 5.8	
				≥ 40	HR 9.5	
			Prediabetes	30–34.9	HR 2.0	
				35.0–39.9	HR 2.5	
				≥ 40	HR 2.9	
Ligthart et al. [34]	2016	Netherlands	NGT	30.0–34.9	AR 43.9% (95% CI 38.6–49.2)	Absolute risk of developing T2D at age 45 years
				≥ 35	AR 56.6% (95% CI 46.7–66.6)	
			Prediabetes	30.0–34.9	AR 87.7% (95% CI 79.4–96.0)	
				≥ 35	AR 80.9% (95% CI 65.9–95.9)	
Zatońska et al. [58]	2020	Poland	Pooled NGT and prediabetes	≥ 30	HR 5.731 (95% CI 2.56–12.82)	Relative to normal weight (BMI 18.5–24.9)

*AR* absolute risk, *BMI* body mass index, *CI* confidence interval, *HR* hazard ratio, *NGT* normal glucose tolerance, *T2D* type 2 diabetes

Only one study split obesity into multiple categories, BMI 30–34.9 kg/m^2^, 35–39.9 kg/m^2^ including a separate one for ≥ 40 kg/m^2^ (class III obesity) [57], the others grouped obesity classes together, including the Zatonska et al. study that used only one category ≥ 30 kg/m^2^ [58]. All studies reported an increased risk of developing T2D, either with absolute risk [34] or relative risk [57, 58].

On the NOS quality assessment scale, two of the studies were of low quality (10 stars) [34, 57] and one of medium quality (11 stars) [58] (see Additional file 1: Table S8).

#### Risk of CV event

Three studies reported the risk of experiencing a CV event in a population with class III obesity and NGT or T2D [59–61] and were carried out in Norway [60], Sweden [59], and the UK [61] (Table 3).

**Table 3:** Included studies reporting the risk of CV events in populations with severe obesity with NGT/T2D

Study	Year	Country	Glycemic status	BMI (kg/m^2^)	Risk of	HR	Note
Edqvist et al. [59]	2019	Sweden	T2D (HbA_1c_ <53 mmol/mol)	≥ 40	MI	1.53 (95% CI 1.25–1.87)	Relative to no T2D
			T2D (HbA_1c_ 53–70 mmol/mol)			1.97 (95% CI 1.63–2.38)	
			T2D (HbA_1c_ <53 mmol/mol)		Hospitalization for heart failure	5.01 (95% CI 3.93–6.39)	
			T2D (HbA_1c_ 53–70 mmol/mol)			5.86 (95% CI 4.57–7.51)	
Iyen et al. [61]	2021	UK	*Not reported*	Morbidly obese (average BMI 49.1)	Stroke/transient ischemic attack	1.04 (95% CI 0.93–1.18)	Relative to overweight (BMI 25–30)
Mørkedal et al. [60]	2014	Norway	NGT	≥ 40	MI	0.9 (95% CI 0.3–2.9)	Relative to BMI < 25
			T2D or prediabetes			1.8 (95% CI 1.1–3.1)	

*BMI* body mass index, *CI* confidence interval, *CV* cardiovascular, *HbA*_*1c*_ glycated hemoglobin, *HR* hazard ratio, *NGT* normal glucose tolerance, *MI* myocardial infarction, *T2D* type 2 diabetes

All studies reported that class III obesity increased the relative risk of a CV event, except in the case of metabolically healthy obesity (obesity without hypertension, dyslipidaemia, and insulin resistance) where there was no increased risk of MI [60].

Two of the studies were of medium quality (11 stars) [59, 60] and one of high quality (13 stars) [61] according to the NOS rating (see Additional file 1: Table S8).

#### Risk of mortality

Three studies reported the risk of mortality from total knee arthroplasty [62–64], four from stroke [65–68], and 22 from CV events in a population with obesity or OWRC [53, 61, 67, 69–87] (Table 4). Studies were carried out in Australia (one study), Europe (14 studies over five countries), and the USA (13 studies).

**Table 4:** Included studies reporting mortality risk from CV events/total knee arthroplasty/stroke in populations with obesity or OWRC

Study	Year	Country	Comorbidity	BMI (kg/m^2^)	Risk			Mortality	Note
Batsis et al. [69]	2014	USA	*Not reported*	≥ 30.0	HR 0.90 (95% CI 0.57–1.43)			CV	Relative to normal weight (BMI 18.5–25.0)
Crotti et al. [71]	2018	Italy	*Not reported*	30.0–39.9	HR 1.01 (95% CI 0.75–1.35)			CV	Relative to normal weight (BMI 18.5–24.9)
				≥ 40	HR 1.32 (95% CI 0.53–3.28)				
Perotto et al. [70]	2013	Italy	T2D	26.8–30.0 (< 65 years old)	HR 1.29 (95% CI 0.62–2.65)			CV	Relative to BMI < 24.2
				> 30.0 (< 65 years old)	HR 0.83 (95% CI 0.39–1.74)				
				26.8–30.0 (≥ 65 years old)	HR 0.86 (95% CI 0.64–1.19)				
				> 30.0 (≥ 65 years old)	HR 0.67 (95% CI 0.45–0.95)				
Ratwatte et al. [72]	2021	Australia	*Not reported*	30.0–39.9	OR 0.42 (95% CI 0.25–0.72)			CV (in-hospital mortality for acute coronary syndrome)	Relative to normal weight (BMI 18.5–24.9)
				> 40.0	OR 0.97 (95% CI 0.45–2.1)				
				30.0–39.9	OR 0.37 (95% CI 0.15–0.91)			CV (6 months post hospitalization for acute coronary syndrome)	
				> 40.0	OR 0.88 (95% CI 0.22–3.54)				
Bhaskaran et al. [73]	2018	UK	*Not reported*	30.0–34.9	HR 1.27 (95% CI 1.25–1.30)			CV	Relative to BMI 22.5–24.9
				35.0–39.9	HR 1.65 (95% CI 1.60–1.70)				
				≥ 40.0	HR 2.49 (95% CI 2.37–2.61)				
Boggs et al. [74]	2011	USA	*Not reported*	30.0–34.9	HR 2.77 (95% CI 1.62–4.73)			CV	Relative to BMI 20.0–24.9
				35.0–49.9	HR 3.90 (95% CI 2.29–6.64)				
Borgeraas et al. [53]	2014	Norway	*Not reported*	≥ 30.0 (male)	HR 1.19 (95% CI 0.76–1.85)			CV	Relative to normal weight (BMI 18.5–24.9)
				≥ 30.0 (female)	HR 0.43 (95% CI 0.14–1.28)				
Champagne-Langabeer et al. [75]	2017	USA	*Not reported*	30.0–34.9	OR 1.20 (95% CI 0.46–3.11)			CV	Relative to normal weight (BMI 18.5–24.9)
				35.0–39.9	OR 0.65 (95% CI 0.14–3.06)				
				≥ 40.0	OR 3.83 (95% CI 1.02–14.41)				
Czernichow et al. [78]	2011	UK	*Not reported*	20.0–35.0	HR 1.10 (95% CI 1.05–1.16)			CV	HR for one higher standard deviation in BMI
Das et al. [79]	2011	USA	*Not reported*	35.0–<40.0	HR 1.10 (95% CI 0.95–1.26)			CV	Relative to BMI 30.0 to <35.0
				≥ 40.0	HR 1.64 (95% CI 1.32–2.03)				
Eeg-Olofsson et al. [80]	2014	Sweden	T2D	25.0–27.4	HR 0.79 (95% CI 0.65–0.96)			CV	Relative to normal weight (BMI 18.5–24.9)
				27.5–29.9	HR 0.92 (95% CI 0.76–1.11)				
				30.0–34.9	HR 1.16 (95% CI 0.97–1.38)				
				≥ 35.0	HR 1.55 (95% CI 1.26–1.90)				
Hotchkiss et al. [85]	2013	UK	*Not reported*	≥ 30.0 (male)	HR 1.13 (95% CI 0.69–1.86)			CV	Relative to normal weight (BMI 18.5–25.0)
				≥ 30.0 (female)	HR 1.51 (95% CI 0.90–2.56)				
Kjøllesdal et al. [86]	2019	Norway	*Not reported*	≥ 30.0	HR 1.66 (95% CI 1.53–1.74)			CV	Relative to BMI < 25.0
Kjøllesdal et al. [87]	2018	Norway	*Not reported*	≥ 30.0 (male, early adulthood)	HR 2.70 (95% CI 1.88–3.78)			CV	Relative to normal weight (BMI 18.5–25.0)
				≥ 30.0 (male, midlife)	HR 1.46 (95% CI 1.25–1.70)				
				≥ 30.0 (female, early adulthood)	HR 1.81 (95% CI 0.82–3.97)				
				≥ 30.0 (female, midlife)	HR 1.21 (95% CI 0.78–1.89)				
Ma et al. [81]	2011	USA	*Not reported*	30.0–<35.0	HR 2.98 (95% CI 2.30–3.87)			CV	Relative to BMI 18.5 to <20.0
				≥ 35.0	HR 5.25 (95% CI 3.89–7.07)				
Ma et al. [82]	2013	USA	*Not reported*	30.0–34.9 (never smoked)	HR 3.22 (95% CI 2.11–4.91) (age 18–44)	HR 1.94 (95% CI 1.51–2.49) (age 45–64)	HR 1.16 (95% CI 1.06–1.28) (age 65–99)	CV	Relative to normal weight (BMI 18.5–24.9) never smoked
				30.0–34.9 (former smoker)	HR 3.68 (95% CI 2.09–6.50) (age 18–44)	HR 2.50 (95% CI 2.05–3.05) (age 45–64)	HR 1.39 (95% CI 1.21–1.59) (age 65–99)		
				30.0–34.9 (current smoker)	HR 7.00 (95% CI 4.66–10.51) (age 18–44)	HR 4.08 (95% CI 3.21–5.18) (age 45–64)	HR 2.21 (95% CI 1.76–2.76) (age 65–99)		
				≥ 35.0 (never smoked)	HR 8.27 (95% CI 4.95–13.81) (age 18–44)	HR 3.26 (95% CI 2.49–4.28) (age 45–64)	HR 1.19 (95% CI 0.98–1.44) (age 65–99)		
				≥ 35.0 (former smoker)	HR 5.39 (95% CI 2.68–10.83) (age 18–44)	HR 3.80 (95% CI 2.80–5.15) (age 45–64)	HR 1.68 (95% CI 1.36–2.07) (age 65–99)		
				≥ 35.0 (current smoker)	HR 12.20 (95% CI 7.73–19.27) (age 18–44)	HR 4.99 (95% CI 3.36–7.42) (age 45–64)	HR 2.17 (95% CI 1.37–3.43) (age 65–99)		
McAuley et al. [83]	2014	USA	*Not reported*	≥ 30.0	HR 1.76 (95% CI 1.17–2.67)			CV	Relative to normal weight (BMI 18.5–24.9)
Payvar et al. [84]	2013	USA	*Not reported*	> 40.0	OR 1.14 (95% CI 1.04–1.25)			CV (all in-hospital mortality post percutaneous coronary intervention	Relative to BMI 20.0–25.0
					OR 1.22 (95% CI 1.08–1.39)			CV (mortality following percutaneous coronary intervention following presentation with STEMI)	
Iyen et al. [61]	2021	UK	*Not reported*	Trajectory 2: 33.7 → 34.9	HR 1.44 (95% CI 1.31–1.58)			CV	(Trajectories over 10 years) Relative to trajectory 1: BMI 28.7 → 29.7
				Trajectory 3: 39.9 → 41.1	HR 2.06 (95% CI 1.86–2.29)				
				Trajectory 4: 49.1 → 49.8	HR 3.31 (95% CI 2.84–3.86)				
Silventoinen et al. [67]	2014	Sweden	*Not reported*	≥ 30.0	HR 2.35 (95% CI 1.81–3.05)			CV	Relative to BMI 20.1–22.4
					HR 2.08 (95% CI 1.56–2.77)			Stroke	
Bo et al. [76]	2012	Italy	*Not reported*	> 30.0 (insulin resistant)	HR 2.43 (95% CI 1.57–3.29)			CV	Relative to BMI < 25.0, insulin sensitive
				> 30.0 (insulin sensitive)	HR 2.95 (95% CI 1.03–3.98)				
Calori et al. [77]	2011	Italy	*Not reported*	≥ 30.0 (insulin resistant)	HR 1.52 (95% CI 1.02–2.26)			CV	Relative to BMI < 30, insulin sensitive
				≥ 30.0 (insulin sensitive)	HR 1.04 (95% CI 0.32–3.30)				
Alvi et al. [62]	2015	USA	*Not reported*	30.0–35.0	OR 0.64 (95% CI 0.05–8.36)			Total knee arthroplasty	Relative to normal weight (BMI 18.5–25.0)
				35.0–40.0	OR 1.56 (95% CI 0.21–11.50)				
				> 40.0	OR 0.90 (95% CI 0.09–9.54)				
Sing et al. [63]	2016	USA	*Not reported*	8.5 → 40.0	0.03% trend in absolute risk of mortality across BMI increase			Total knee arthroplasty	
Sundara et al. [64]	2019	USA	*Not reported*	30.0–39.9	OR 0.125 (95% CI 0.011–1.385)			Total knee arthroplasty	Relative to normal weight (BMI 18.5–24.9)
				≥ 40.0	OR 0.000 (95% CI 0.000)				
Andersen et al. [65]	2015	Denmark	*Not reported*	≥ 30.0	HR 0.53 (95% CI 0.49–0.57)			Stroke	Relative to normal weight (BMI 18.5–24.9)
Hoffman et al. [66]	2020	USA	*Not reported*	30–39.9	OR 0.62 (95% CI 0.56–0.69)			Stroke	Relative to BMI < 30.0
				≥ 40.0	OR 0.76 (95% CI 0.66–0.88)				
Skolarus et al. [68]	2014	USA	*Not reported*	33.0–34.0	HR 0.75^a^			Stroke	Relative to BMI 24
				35.0–36.0	HR 0.6^a^				
				37.0–38.0	HR 0.5^a^				
				39.0–41.0	HR 1.0^a^				
				≥ 42.0	HR 1.5^a^				

*BMI* body mass index, *CI* confidence interval, *CV* cardiovascular, *HR* hazard ratio, *OR* odds ratio, *STEMI* ST-elevation myocardial infarction, *T2D* type 2 diabetes

^a^Estimated results taken from graph (not tabulated)

Almost all studies measured relative risk. Most studies reported risk of mortality increased with higher BMI counts, however, not all studies agreed. Two studies reporting on mortality following total knee arthroplasty showed no association with weight [63, 64]. The often-documented paradoxical link with mortality following stroke was reported in two of three studies [65, 66]. For CV linked mortality, a paradoxical link was reported in two studies [69, 72], two reported no association [78, 85], and in others a more nuanced association was demonstrated [53, 61, 70, 76, 77, 83].

The NOS rating for the studies on mortality from total knee arthroplasty were all of high quality. Of studies detailing mortality from stroke two were assessed as low quality (10 stars) [66, 67], one medium (11 stars) [68], and one high (13 stars) [65]. The majority (12) of studies reporting on risk of mortality from CV events were of medium quality [69–71, 73, 77, 81–87], there were seven of low [53, 67, 72, 74–76, 80], and three of high quality [61, 78, 79].One study achieved the maximum star in this SLR of 14, Das et al. [79]. They assessed the impact of class III obesity on care and outcomes on a specific type of MI, ST-segment MI, in a cohort of 49,329 subjects. Class III obesity was associated with younger age at MI presentation, as well as a higher prevalence of diabetes, hypertension, and dyslipidaemia but less extensive coronary artery disease and better left ventricular systolic function, though in-hospital mortality remains significantly higher than those with class I obesity. Patients with class III obesity and MI were more than a decade younger than normal weight counterparts and more likely to be women and of African American descent.

## Discussion

This SLR represents the current state on available data for risk equations that can predict the incidence of complications in populations with OWRC and obesity. It further exposes the unmet need for obesity specific equations, the limited ability of these equations to estimate risk for higher BMI categories, and the inconsistency of results between different risk equations on the same populations with OWRC and obesity. There is a clear necessity for new data or adjustment of the current risk equations to allow for more robust health economic modelling that can inform accurate decision-making strategies for the management of people living with obesity and specifically class III obesity.

It is important to validate risk equations in external cohorts to assess their usability in different populations and regions [37]. Otherwise potential problems arise when developing multivariable risk prediction models, including overestimating performance and overfitting [6, 21], meaning that the equation’s performance cannot be applied to external data cohorts not used to derive the model [88]. Beyond this, Mathur et al. acknowledges that even though many risk prediction tools have been developed, for T2D for example, relatively few validated risk scoring systems have demonstrated real-world application [35].

Only six studies reported on external validation of the risk equations [23, 35, 38, 41, 43, 44] (eight out of the total 60 included studies used external validation [23, 35, 38, 41, 43, 44, 79, 84]). All of the externally validated equations calculate the risk of T2D and CVD, but none calculated the risk of developing other complications commonly associated with obesity or overweight. However, even when comparing the performance of frequently used and validated risk equations in T2D and CVD, Gray et al. (2014 and 2015) demonstrated that they produce different outcomes and assessments of risk [23, 44]. This highlights the complex nature of these equations and the impact of the specific risk predictors and how they are calibrated.

A common theme in the literature is the lack of granularity among BMI classes of obesity used to develop or validate the published risk equations. People living with obesity were typically characterized as having a BMI of ≥ 30 kg/m^2^ and not broken down further. Therefore, it can be assumed that these risk equations may work well up to a certain BMI level before they lose sensitivity. Only 17% of the risk equations detailed the BMI of the cohort used to develop and/or validate the risk equation by obesity class beyond ≥ 30 kg/m^2^ [30, 42, 49, 52, 56]. Most (62%) risk equation studies did not report any BMI breakdown of the cohort used or only reported the average BMI at best [23, 29, 31–35, 37, 39–41, 44, 47, 48, 50, 51, 54, 55]. No risk equation was developed or validated using a cohort separately distinguished beyond BMI ≥ 40 kg/m^2^. Even with the lack of risk equation specificity for class III obesity some still calculated the risk of developing a complication more granularly, beyond this point. No explanation or insight was identified for including higher BMI groups into these risk equations, or how, if at all, a presumably small sample within the highest BMI groups was dealt with accurately to create these equations. The representation of each population in the development and validation process lacked clarity. Therefore, the accuracy of the calibration for calculating class III obesity specific risk and the subsequent performance of the risk equation is contentious.

Absolute or relative risks, as identified in this SLR, can be used in calibration of risk equations to improve accuracy in higher BMI populations, or in populations not well represented (e.g., those with specific complications). Poorly calibrated risk equations can be misleading and potentially harmful in clinical decisions. The identified calibration targets can either be used to calibrate an existing model to better fit populations with obesity or overweight with more precision, or to confirm if new risk equations are robust. Such risk measures can be used as calibration targets in simulations to help optimize risk equation parameters, for example [89]. This approach would ensure greater accuracy and generalizability in subpopulations currently not well represented.

As for the identified studies reporting absolute or proportional risk for development of T2D, CV events, and mortality in specific populations with obesity or OWRC, those assessing outcome and complication development often failed to distinguish multiple obesity classes and lacked precision. The results of different studies on the same topic were not always in agreement, making it difficult to draw concrete and meaningful conclusions. For example, different studies reported higher, lower, and no association for stroke mortality risk in association with obesity and OWRC [65–68]. Also, for CV mortality risk in association with obesity and OWRC some studies have reported a paradoxical link [69, 72] or no association [78, 85], countering increased [67, 74, 79–81, 84, 86, 87] or nuanced risk [53, 61, 70, 76, 77, 83] from other studies. More consistency in studies is needed, with more granulated risk predictors (e.g., BMI) as well as a wider range of risk factors being explored, such as historical and ongoing weight increases rather than just the current BMI. This would allow robust outcomes to inform calibration and validation for risk equations.

The choice of risk predictor usage and its weighting depend on the outcome and population of interest. There is not one catch all risk predictor for weight related complications. If we consider patients with T2D, BMI is a strong independent risk factor of heart failure in a stepwise fashion, but not for acute myocardial infarction [49]. In addition, risk predictor choice may be limited by availability. For example, in primary care settings potential risk predictors such as liver function tests, pulse rate, and BMI are not always recorded [31], so what makes a good risk predictor also lies in its ease of use. In the absence of singular strong risk predictors the use of multiple risk predictors that individually slightly increase risk can still identify a high risk group [29].

While not perfect, risk equations have been used to inform both clinical and health technology assessment (HTA) decision making in obesity and overweight [90]. The UK’s NICE guidelines recommend using the Cambridge Risk Score, FINDRISC, Leicester Risk Assessment, and QDiabetes for calculating the risk of developing T2D [91]. In a comparison of the four, Gray et al. concluded that either QDiabetes or FINDRISC are the most practical and cost-effective tools for NICE decision making [23]. Mathur et al. concluded that NICE could use the QDScore to identify high T2D risk people, however this SLR did not identify any other studies comparing this risk equation with other tools and therefore this may not be substantiated [35]. In CVD, NICE recommend the use of QRISK2 because Framingham equations overestimate the risk in a UK population and QRISK2 is widely used in UK general practice [41, 48]. Indeed, when compared with three other validated CVD risk equations, QRISK2 was reported to have high accuracy and is frequently updated, making it most appropriate for decision making [44].

## Conclusion

The studies included in this SLR used risk equations adapted for the target population but not built specifically for them and BMI categories that were inconsistently reported and adhered to, providing limited potential for comparison or accurate risk assessment. The included studies calculating absolute or proportional risk of specific disease outcomes can be built upon to inform more robust risk equations and so, provide more reliable health economic models to aid decision makers.

The accuracy of the risk equations when applied to other populations and between BMI classes, in particular class III obesity, remains questionable. The majority of risk equations lacked external validation and those that did only calculated the risk of developing T2D and CVD. The risk equations were largely limited to European cohorts and made little attempt to delineate between obesity classes, particularly with regard to higher BMI groups.

This SLR also identified a number of studies reporting absolute risks of developing T2D, CVD or mortality from CVD, T2D, or total knee arthroplasty in populations with obesity and OWRC. Such absolute scores can be used as calibration targets when calibrating risk equations, to help with accuracy in populations with higher BMIs or in subgroups of populations with obesity or OWRC.

## Implications in practice and recommendations

This SLR has highlighted several gaps to be addressed in future work, with real-world implications. Further work is needed to externally validate equations calculating the risk of populations with obesity or OWRC developing a complication. This should also include validating these equations in diverse populations to ensure generalizability. It should additionally ensure cohorts with obesity are clearly broken down by class, especially focusing on class III obesity, again to ensure accuracy in this population. We identified externally validated risk equations used to predict T2D and CV risk but, particularly for other obesity associated complications, found limited data and further development for all outcomes is required. Other approaches could include utilizing or generating real-world evidence to fill some of the data gaps and recalibrating existing risk equations. This will help to inform robust evaluations of obesity and overweight treatments, HTA decision making, and beyond. More studies informing on the absolute or proportional risk of a wider range of risk factors, specifically for populations ranging from overweight through class I, II, and III obesity, would help to calibrate and improve both existing and new risk equations for these populations.

Many of the points raised above can enhance future risk equations and study design. We have discussed in detail the importance of categorising BMI and recommend that future studies harmonise through using, for example, the relevant WHO BMI scales, and avoid grouping weight categories. In addition to identifying specific risk, it will allow individual data sets to be pooled and reanalysed. Additional guidelines could include new risk equations focusing on weight associated complications that are poorly predicted by the existing risk equations such as metabolic abnormalities and risk of developing T2D. Also, the identification of new risk predictors through methods such as genetic profiling and related to metabolic alterations would add value, while new data can support existing risk predictors and determine how they are best weighted for each equation.

As scientists increasingly understand the value of data sharing and open-source codes, big data will further change the way we predict risk. As an example, all modern predictive models of financial market risks are based on data driven analysis exploiting machine learning. One of the main reasons this is not done in medicine yet is due to the data deficit needed for a multifactorial analysis. Only a few areas in the medical field, such as radiomics, have harnessed this power with data driven analysis for diagnosis and prognosis of several diseases. As more data is gathered, risk modelling for weight related outcomes will become more powerful to accurately predict outcomes for populations living with obesity, populations that are growing rapidly in many parts of the world.

## Limitations

This SLR was limited to countries by geographical scope (Australia, Canada, Europe, and the USA) and therefore may have excluded potentially useful risk equations developed in other regions. Results predominantly reflected European studies, while Canada and the USA are not well represented. Furthermore, because a direct comparison of the risk equations was not possible due to each being developed or calculated using different methods for different purposes, a judgment on how each risk score performs is the opinion of the user or decision-maker in a particular context. Finally, obesity may not be a single homogenous disease. Thus, if there are different obesity subtypes leading to the same BMI in different people then a single unifying risk equation may not be possible, but rather better diagnostic ability of these obesity subtypes may be required to refine future risk equations.

## Supplementary Information

**Additional file 1: Table S1.** Inclusion and exclusion criteria used in the systematic literature review. **Table S2.** Search strings used for the Embase search. **Table S3.** Search strings used for the PubMed search. **Table S4.** Data extraction categories. **Table S5.** The adapted Newcastle–Ottawa Scale. **Table S6.** Studies excluded following the full-text screening. **Table S7.** Included studies. **Table S8.** Quality assessment of the included studies using the adapted Newcastle Ottawa Scale.

## Data Availability

The following electronic databases were searched as standard evidence sources: MEDLINE, MEDLINE In-Process and e-publications ahead-of-print (via PubMed), Embase (via Embase.com), the technology appraisal submissions to the National Institute for Clinical Excellence (NICE) using risk equations, and bibliographic reference lists from included studies. Search strings can be found in Additional file 1: Table S2 (Embase) and Additional file 1: Table S3 (PubMed).

## Abbreviations

ACE: Angiotensin converting enzyme
AR: Absolute risk
BMI: Body mass index
CI: Confidence interval
CV: Cardiovascular
CVD: Cardiovascular disease
FINDRISC: Finnish Diabetes Risk Score
HbA_1c_: Glycated hemoglobin
HR: Hazard ratio
HTA: Health technology assessment
MI: Myocardial infarction
NGT: Normal glucose tolerance
NICE: National Institute for Clinical Excellence
NOS: Newcastle Ottawa Scale
OR: Odds ratio
OWRC: Overweight with a weight-related complication
PRISMA: Preferred Reporting Items for Systematic Reviews and Meta-Analyses
SLR: Systematic literature review
STEMI: ST-elevation myocardial infarction
T2D: Type 2 diabetes
WHO: World Health Organization
