# Supplementary material for: Understanding the risk of developing weight-related complications associated with different body mass index categories: a systematic review
Source: Diabetol Metab Syndr. 2022 Dec 7;14:186. doi: 10.1186/s13098-022-00952-4 (PMC9727983; doi:10.1186/s13098-022-00952-4)
Supplement: Supplementary file 1 — Additional file 1: Table S1. Inclusion and exclusion criteria used in the systematic literature review. Table S2. Search strings used for the Embase search. Table S3. Search strings used for the PubMed search. Table S4. Data extraction categories. Table S5. The adapted Newcastle–Ottawa Scale. Table S6. Studies excluded following the full-text screening. Table S7. Included studies. Table S8. Quality assessment of the included studies using the adapted Newcastle Ottawa Scale. [file 13098_2022_952_MOESM1_ESM.docx]

**Additional file 1:**

Contents

[Table S1. Inclusion and exclusion criteria used in the systematic literature review 2](#_Toc104977417)

[Table S2. Search strings used for the Embase search 4](#_Toc104977418)

[Table S3. Search strings used for the PubMed search 7](#_Toc104977419)

[Table S4. Data extraction categories 10](#_Toc104977420)

[Table S5. The adapted Newcastle-Ottawa Scale [6] 11](#_Toc104977421)

[Table S6. Studies excluded following the full-text screening 12](#_Toc104977422)

[Table S7. Included studies 27](#_Toc104977423)

[Table S8. Quality assessment of the included studies using the adapted Newcastle Ottawa Scale 30](#_Toc104977424)

[References 33](#_Toc104977425)

# Table S1. Inclusion and exclusion criteria used in the systematic literature review

| **PICOS** | **Inclusion criteria** | **Exclusion criteria** |
| --- | --- | --- |
| Participants | - Obese adults (≥18) with a BMI ≥30 kg/m^2^ - Overweight adults (≥18) with a BMI ≥25 kg/m^2^ with the presence of at least one weight-related comorbidity ^a^ | - Adults (≥18) with BMI 25–30 kg/m^2^ without the presence of at least one comorbidity ^a^ - Adults (≥18) with a BMI <25 kg/m^2^ - Pediatric populations (<18) - In vitro data - Animal data - Mixed disease populations (i.e., mixed obese/non-obese) without obese data reported separately - Mixed child/adult populations without adult data reported separately |
| Interventions/ Comparators | - No restrictions or none | N/A |
| Outcomes  Review question  1 ^d^ | - Risk equation – in a population with obesity or overweight with at least one weight-related comorbidity ^a^ – calculating risk of developing at least one additional comorbidity: prediabetes, T2D, CVD, heart disease (incl. HfpEF), ACS (unstable angina or MI), cerebrovascular accident (incl. TIA, stroke), musculoskeletal disorders, knee replacement/arthroplasty, OSA | - Risk equations where outcomes by BMI or change in BMI are not reported - Studies not reporting the equation |
| Outcomes  Review question  2 ^e^ | - Absolute risk ^b^ or proportional risk ^c^ of developing T2D (in an obese population with prediabetes or NGT) | - Glucose tolerance status not reported - Non-obese individuals (i.e.,  BMI <30) - Time period for statistic not reported - HRs where baseline hazard for reference group not reported - Proportional risk outcomes not compared to either lower BMI group or to normal weight group |
| Outcomes  Review question  3 ^f^ | - Absolute risk ^b^ or proportional risk ^c^ of having a CV event (in a morbidly obese population with NGT or T2D) | - Not morbidly obese (i.e.,  BMI <40) - Time period for statistic not reported - HRs where baseline hazard for reference group not reported - Proportional risk outcomes not compared to either lower BMI group or to normal weight group |
| Outcomes  Review question  4 ^g^ | - Absolute risk ^b^ or proportional risk ^c^ of mortality from knee surgery, stroke, or acute CV events (in an obese, or overweight with comorbidities ^a^, population) | - BMI <25 - Overweight comorbidity ^a^ not confirmed - Time period for statistic not reported - HRs where baseline hazard for reference group not reported - Proportional risk outcomes not compared to either lower BMI group or to normal weight group |
| Study design | - Population-based cohort analyses or other large-scale study (e.g., registry, longitudinal study, questionnaire, or case-control study data with appropriate sampling) from which generalization nationwide or regionally is viable | - Study sample from a small region from which it would be un-robust to generalize |
| Publication type and status | - Peer-reviewed articles or errata | - Letters - Editorials - Notes - Comment articles - Abstract-only articles (question 4 only) - News articles |
| Country | - Australia, Canada, Europe, UK, USA | - Africa, Asia, South America |
| Publication year | - Publication year 2011–2021 (past 10 years) | - Publication year <2011 (over 10 years) |
| *ACS* acute coronary syndrome, *BMI* body mass index, *CV* cardiovascular, *CVD* cardiovascular disease, *HfpEF* heart failure preserved ejection fraction, *HR* hazard ratio, *MI* myocardial infarction, *NGT* normal glucose tolerance, *OSA* obstructive sleep apnea, *PICOS* Population, Intervention, Comparison, Outcomes, and Study, *T2D* type 2 diabetes, *TIA* transient ischemic attack  ^a^ T2D, hypertension, dyslipidemia, obstructive sleep apnea, or CVD; ^b^ risk, rate, odds, hazard outcomes; ^c^ risk ratio, odds ratio, HR, relative risk, risk reduction; ^h^ adults with a BMI ≥30 kg/m^2^; ^i^ a BMI ≥25 kg/m^2^; ^j^ prediabetes, T2D, CVD, heart disease, ACS (including MI and unstable angina), stroke (including TIA), musculoskeletal disorders, knee replacement, sleep apnea; ^d^Review question 1: What risk equations have been published to calculate the risk of people with obesity ^h^ or overweight ^i^ with at least one weight-related comorbidity ^a^ developing at least one additional comorbidity ^j^?  ^e^ Review question 2: What is the risk of an adult with a BMI ≥30 and NGT or prediabetes developing T2D?  ^f^ Review question 3: What is the risk of an adult with a BMI ≥40 and NGT or T2D experiencing a CV event?  ^g^ Review question 4: What is the risk of mortality arising from knee surgery, stroke, and acute CV events in people who are obese ^h^ or overweight ^i^ with at least one weight-related comorbidity ^a^? | | |

# Table S2. Search strings used for the Embase search

| No. | Query | Results |
| --- | --- | --- |
| #1 | 'morbid obesity':de OR superobesity:ab,ti OR 'super obesity':ab,ti OR superobese:ab,ti OR 'super obese':ab,ti OR (((morbid* OR severe) NEAR/3 (obese OR obesity)):ab,ti) | 43,431 |
| #2 | 'obesity':de OR 'abdominal obesity':de OR 'diabetic obesity':de OR 'obese patient'/exp OR obesity:ab,ti OR obese:ab,ti | 607,739 |
| #3 | overweight:ab,ti OR 'over weight':ab,ti | 110,963 |
| #4 | #1 OR #2 OR #3 | 614,253 |
| #5 | 'body mass'/exp OR bmi:ab,ti OR 'body mass index':ab,ti OR 'bodymass index':ab,ti OR 'bodymassindex':ab,ti OR 'body massindex':ab,ti OR 'quetelet index':ab,ti | 610,333 |
| #6 | 'metabolic syndrome x':de OR 'waist circumference':de OR 'waist hip ratio':de OR 'high risk population'/exp OR 'intermediate risk population'/exp OR 'low risk population'/exp OR 'high risk patient'/exp OR 'intermediate risk patient'/exp OR 'low risk patient'/exp OR 'insulin resistance'/exp OR ((waist NEAR/3 circumference):ab,ti) OR ((waist NEAR/3 hip NEAR/3 ratio):ab,ti) OR 'metabolic syndrome':ab,ti OR 'general population':ab,ti OR 'normal population':ab,ti OR 'high risk':ab,ti OR 'intermediate risk':ab,ti OR 'low risk':ab,ti OR ((insulin NEAR/3 resist*):ab,ti) OR 'class i':ab,ti OR 'class 1':ab,ti OR 'class ii':ab,ti OR 'class 2':ab,ti OR 'class iii':ab,ti OR 'class 3':ab,ti OR ((weight* NEAR/3 (loss OR lost OR reduc* OR change OR gain OR fluctuation OR variation OR trajectory OR body OR related)):ab,ti) | 1,738,872 |
| #7 | 'impaired glucose tolerance'/exp OR prediabetes:ab,ti OR 'pre diabetes':ab,ti OR prediabetic:ab,ti OR ((impair* NEAR/3 glucose NEAR/3 tolerance):ab,ti) OR ((diabetes NEAR/3 (chemical OR potential OR latent)):ab,ti) | 48,350 |
| #8 | 'non insulin dependent diabetes mellitus'/exp OR t2dm:ab,ti OR t2d:ab,ti OR dm2:ab,ti OR 'dm 2':ab,ti OR niddm:ab,ti OR ((diabetes NEAR/3 ('type 2' OR type2 OR 'type ii' OR typeii)):ab,ti) | 325,099 |
| #9 | 'cardiovascular disease':de OR 'coronary artery disease':de OR 'diabetic angiopathy'/exp OR 'thromboembolism'/exp OR 'heart infarction'/exp OR cvd:ab,ti OR angiocardiopathy:ab,ti OR thromboembolism:ab,ti OR thrombosis:ab,ti OR vte:ab,ti OR embolism:ab,ti OR (((ischemic OR ischaemic OR coronary) NEAR/3 'heart disease'):ab,ti) OR 'coronary artery disease':ab,ti OR (((cardiovascular OR angiocardiovascular OR cv) NEAR/3 (disease OR complication* OR disorder* OR event* OR endpoint* OR outcome*)):ab,ti) OR ((diabetic NEAR/3 angiopathy):ab,ti) | 1,574,687 |
| #10 | 'heart disease':de OR 'heart aneurysm'/exp OR 'heart failure'/exp OR 'ischemic heart disease':de OR 'major adverse cardiac event'/exp OR 'heart ejection fraction'/exp OR 'heart disease':ab,ti OR 'heart aneurysm':ab,ti OR 'heart failure':ab,ti OR hfpef:ab,ti | 1,024,516 |
| #11 | 'acute coronary syndrome'/exp OR 'angina pectoris'/exp OR 'acute coronary syndrome*':ab,ti OR ((acs NEAR/3 (acute OR coronary OR syndrome*)):ab,ti) OR ((angina NEAR/3 unstable):ab,ti) OR (((myocardial OR heart) NEAR/3 infarct*):ab,ti) | 424,073 |
| #12 | 'cerebrovascular accident'/exp OR 'transient ischemic attack'/exp OR stroke:ab,ti OR apoplexy:ab,ti OR apoplexia:ab,ti OR ((cerebrovascular NEAR/3 (accident$ OR lesion OR arrest OR failure OR injury OR insufficiency OR insult)):ab,ti) OR (((cerebro OR cerebral OR brain OR cerebrum) NEAR/3 (vasculopathy OR accident OR attack OR insult* OR insufficiency)):ab,ti) OR ((seizure NEAR/3 (ischaemic OR ischemic)):ab,ti) OR ((cva NEAR/3 (cerebrovascular OR attack)):ab,ti) OR ((tia NEAR/3 (transient OR ischaemic OR ischemic OR attack)):ab,ti) | 534,063 |
| #13 | 'musculoskeletal disorders'/exp OR ((musculoskeletal NEAR/3 disorder$):ab,ti) | 11,217 |
| #14 | 'knee replacement'/exp OR 'knee arthroplasty':de OR ((knee NEAR/3 (replace* OR arthroplasty)):ab,ti) | 53,316 |
| #15 | 'sleep disordered breathing'/exp OR (((sleep OR nocturnal) NEAR/3 (apnea OR apnoea)):ab,ti) OR ((osa NEAR/3 (obstructive OR sleep OR apnea OR apnoea)):ab,ti) | 89,647 |
| #16 | #7 OR #8 OR #9 OR #10 OR #11 OR #12 OR #13 OR #14 OR #15 | 2,983,713 |
| #17 | 'disease risk assessment'/exp OR 'scoring system'/exp OR 'prediction'/exp OR 'prediction model'/exp OR ((risk NEAR/3 (predict* OR equation$ OR score$ OR scale$)):ab,ti) | 775,130 |
| #18 | 'population research'/exp OR 'population'/exp OR 'population risk'/exp OR 'population based':ab,ti OR ((population NEAR/3 (large OR sample OR level)):ab,ti) OR 'whole country':ab,ti OR national:ab,ti OR international:ab,ti OR nationwide:ab,ti OR 'nation wide':ab,ti OR 'large scale':ab,ti OR regional:ab,ti OR 'region wide':ab,ti OR ((representative NEAR/3 (study OR sample OR population)):ab,ti) | 2,583,212 |
| #19 | 'incidence'/exp OR 'hazard ratio'/exp OR incidence:ab,ti OR incident:ab,ti OR (('area under' NEAR/3 curve):ab,ti) OR auc:ab,ti OR hazard:ab,ti OR rate:ab,ti OR risk:ab,ti OR odds:ab,ti OR likelihood:ab,ti | 6,885,182 |
| #20 | 'europe'/exp OR 'north america':de OR 'canada'/exp OR 'united states'/exp OR 'australia'/exp OR (europe*:ab,ti AND eu:ab,ti) OR austria*:ab,ti OR belgium:ab,ti OR belgian:ab,ti OR brussels:ab,ti OR flanders:ab,ti OR wallonia:ab,ti OR benelux:ab,ti OR 'channel islands':ab,ti OR guernsey:ab,ti OR jersey:ab,ti OR france:ab,ti OR french:ab,ti OR corsica*:ab,ti OR german*:ab,ti OR ((german NEAR/3 republic):ab,ti) OR prussia*:ab,ti OR ireland:ab,ti OR irish:ab,ti OR 'isle of man':ab,ti OR liechtenstein:ab,ti OR luxembourg:ab,ti OR monaco:ab,ti OR netherlands:ab,ti OR dutch:ab,ti OR scandinavia*:ab,ti OR denmark:ab,ti OR danish:ab,ti OR 'faroe islands':ab,ti OR finland:ab,ti OR finnish:ab,ti OR aland:ab,ti OR greenland:ab,ti OR iceland*:ab,ti OR norway:ab,ti OR norwegian:ab,ti OR svalbard:ab,ti OR spitsbergen:ab,ti OR 'jan mayen':ab,ti OR sweden:ab,ti OR swedish:ab,ti OR switzerland:ab,ti OR swiss:ab,ti OR 'united kingdom':ab,ti OR uk:ab,ti OR 'great britain':ab,ti OR british:ab,ti OR 'northern ireland':ab,ti OR england:ab,ti OR english:ab,ti OR scotland:ab,ti OR scottish:ab,ti OR wales:ab,ti OR welsh:ab,ti OR andorra:ab,ti OR gibraltar:ab,ti OR greece:ab,ti OR greek:ab,ti OR italy:ab,ti OR italian:ab,ti OR aosta:ab,ti OR 'friuli venezia':ab,ti OR sardinia*:ab,ti OR sicily:ab,ti OR sicilian:ab,ti OR trentino:ab,ti OR 'south tyrol':ab,ti OR malta:ab,ti OR maltan:ab,ti OR portugal:ab,ti OR portuguese:ab,ti OR azores:ab,ti OR madeira:ab,ti OR 'san marino':ab,ti OR spain:ab,ti OR spanish:ab,ti OR 'balearic islands':ab,ti OR 'basque country':ab,ti OR 'canary islands':ab,ti OR catalonia*:ab,ti OR galicia*:ab,ti OR navarre:ab,ti OR valencian:ab,ti OR 'vatican city':ab,ti OR albania*:ab,ti OR armenia*:ab,ti OR azerbaijan:ab,ti OR 'nagorno karabakh':ab,ti OR 'balkan peninsula':ab,ti OR 'baltic states':ab,ti OR estonia*:ab,ti OR latvia*:ab,ti OR lithuania*:ab,ti OR belarus*:ab,ti OR bosnia*:ab,ti OR herzegovina*:ab,ti OR herzegowina*:ab,ti OR srpska:ab,ti OR yugoslavia*:ab,ti OR bulgaria*:ab,ti OR croatia*:ab,ti OR 'czech republic':ab,ti OR georgia*:ab,ti OR abkhazia:ab,ti OR 'south ossetia':ab,ti OR hungary:ab,ti OR hungarian:ab,ti OR kosovo*:ab,ti OR moldova*:ab,ti OR montenegro:ab,ti OR poland:ab,ti OR polish:ab,ti OR macedonia*:ab,ti OR romania*:ab,ti OR serbia*:ab,ti OR vojvodina:ab,ti OR slovakia*:ab,ti OR slovenia*:ab,ti OR ukrain*:ab,ti OR crimea*:ab,ti OR america$:ab,ti OR 'united states':ab,ti OR usa:ab,ti OR 'u s a':ab,ti OR alabama:ab,ti OR alaska:ab,ti OR arizona:ab,ti OR arkansas:ab,ti OR california:ab,ti OR colorado:ab,ti OR connecticut:ab,ti OR delaware:ab,ti OR 'district of columbia':ab,ti OR florida:ab,ti OR georgia:ab,ti OR hawaii:ab,ti OR idaho:ab,ti OR illinois:ab,ti OR indiana:ab,ti OR iowa:ab,ti OR kansas:ab,ti OR kentucky:ab,ti OR louisiana:ab,ti OR maine:ab,ti OR maryland:ab,ti OR massachusetts:ab,ti OR michigan:ab,ti OR minnesota:ab,ti OR mississippi:ab,ti OR missouri:ab,ti OR montana:ab,ti OR nebraska:ab,ti OR nevada:ab,ti OR 'new hampshire':ab,ti OR 'new jersey':ab,ti OR 'new mexico':ab,ti OR 'new york':ab,ti OR 'north carolina':ab,ti OR 'north dakota':ab,ti OR ohio:ab,ti OR oklahoma:ab,ti OR oregon:ab,ti OR pennsylvania:ab,ti OR 'rhode island':ab,ti OR 'south carolina':ab,ti OR 'south dakota':ab,ti OR tennessee:ab,ti OR texas:ab,ti OR utah:ab,ti OR vermont:ab,ti OR virginia:ab,ti OR washington:ab,ti OR wisconsin:ab,ti OR wyoming:ab,ti OR 'new england':ab,ti OR 'appalachian region':ab,ti OR 'great lakes region':ab,ti OR 'mid atlantic region':ab,ti OR canada:ab,ti OR canadian:ab,ti OR alberta*:ab,ti OR 'british columbia':ab,ti OR manitoba*:ab,ti OR 'new brunswick':ab,ti OR newfoundland:ab,ti OR labrador:ab,ti OR 'northwest territories':ab,ti OR 'nova scotia':ab,ti OR nunavut:ab,ti OR ontario:ab,ti OR 'prince edward island':ab,ti OR quebec:ab,ti OR saskatchewan:ab,ti OR yukon:ab,ti OR australia*:ab,ti OR ((nsw NEAR/3 (new OR south)):ab,ti) OR 'northern territory':ab,ti OR queensland:ab,ti OR tasmania*:ab,ti OR victoria*:ab,ti | 5,977,590 |
| #21 | [1 OR (#5 AND #6]) AND #16 AND #17 AND #18 AND #19 AND #20 | 1,802 |
| #22 | 'normal glucose tolerance'/exp OR 'glucose tolerance'/exp OR ((normal NEAR/3 glucose NEAR/3 tolerance):ab,ti) OR ((ngt NEAR/3 (normal OR glucose OR tolerance)):ab,ti) | 29,137 |
| #23 | (#1 OR #2 OR (#5 AND #6)) AND [2 OR #22] AND #8 AND #18 AND #19 AND #20 | 697 |
| #24 | (#1 OR (#5 AND #6)) AND [3 OR #22] AND [4 OR #10 OR #11 OR #12] AND #18 AND #19 AND #20 | 775 |
| #25 | 'hypertension'/exp OR hypertension:ab,ti OR hypertensive$:ab,ti OR ((high NEAR/3 'blood pressure'):ab,ti) | 1,018,155 |
| #26 | 'dyslipidemia'/exp OR dyslipidemia$:ab,ti OR dyslipaemia:ab,ti OR dyslipemia:ab,ti OR dyslipidaemia$:ab,ti OR lipidaemia:ab,ti OR lipidemia:ab,ti | 91,925 |
| #27 | #8 OR #9 OR #15 OR #25 OR #26 | 2,633,901 |
| #28 | 'mortality'/exp OR 'survival analysis'/exp OR mortality:ab,ti OR death$:ab,ti OR 'case fatality rate':ab,ti | 2,494,526 |
| #29 | (#1 OR #2 OR (#3 AND #27)) AND [5 OR #6] AND [4 OR #10 OR #11 OR #12 OR #14] AND #18 AND #20 AND #28 NOT 'conference abstract'/it | 1,446 |
| #30 | #21 OR #23 OR #24 OR #29 | 4,150 |
| #31 | 'cohort analysis'/exp OR 'case control study'/exp OR 'validation process':de OR 'register'/exp OR 'prospective study'/exp OR 'controlled study'/exp OR 'longitudinal study'/exp OR 'questionnaire'/exp OR cohort:ab,ti OR 'case cohort':ab,ti OR 'case base':ab,ti OR 'case control':ab,ti OR register:ab,ti OR registry:ab,ti OR registries:ab,ti OR validat*:ab,ti OR longitudinal:ab,ti OR 'controlled study':ab,ti OR questionnaire$:ab,ti | 10,578,298 |
| #32 | #30 AND #31 | 2,735 |
| #33 | #32 AND ('chapter'/it OR 'conference review'/it OR 'editorial'/it OR 'letter'/it OR 'note'/it) | 23 |
| #34 | #32 NOT #33 | 2,712 |
| #35 | #34 AND ([adolescent]/lim OR [child]/lim OR [fetus]/lim OR [infant]/lim OR [newborn]/lim OR [preschool]/lim OR [school]/lim OR child*:ti) | 244 |
| #36 | 'adult'/exp OR adult*:ab,ti | 9,978,232 |
| #37 | #35 NOT #36 | 61 |
| #38 | #34 NOT #37 AND [2010-2021]/py AND [embase]/lim | 1,928 |

# Table S3. Search strings used for the PubMed search

| No. | Query | Results |
| --- | --- | --- |
| #1 | Search: "Obesity, Morbid"[Mesh:NoExp] OR superobesity[tiab] OR "super obesity"[tiab] OR superobese[tiab] OR "super obese"[tiab] OR ((morbid*[tiab] OR severe[tiab]) AND (obese[tiab] OR obesity[tiab])) | [52,998](https://pubmed.ncbi.nlm.nih.gov/?term=%E2%80%9CObesity%2C+Morbid%E2%80%9D%5BMesh%3ANoExp%5D+OR+superobesity%5Btiab%5D+OR+%E2%80%9Csuper+obesity%E2%80%9D%5Btiab%5D+OR+superobese%5Btiab%5D+OR+%E2%80%9Csuper+obese%E2%80%9D%5Btiab%5D+OR+%28%28morbid%2A%5Btiab%5D+OR+severe%5Btiab%5D%29+AND+%28obese%5Btiab%5D+OR+obesity%5Btiab%5D%29%29&ac=no&sort=relevance) |
| #2 | Search: "Obesity"[Mesh:NoExp] OR "Obesity, Abdominal"[Mesh:NoExp] OR obesity[tiab] OR obese[tiab] | [362,225](https://pubmed.ncbi.nlm.nih.gov/?term=%E2%80%9CObesity%E2%80%9D%5BMesh%3ANoExp%5D+OR+%E2%80%9CObesity%2C+Abdominal%E2%80%9D%5BMesh%3ANoExp%5D+OR+obesity%5Btiab%5D+OR+obese%5Btiab%5D&ac=no&sort=relevance) |
| #3 | Search: overweight[tiab] OR "over weight"[tiab] | [74,255](https://pubmed.ncbi.nlm.nih.gov/?term=overweight%5Btiab%5D+OR+%E2%80%9Cover+weight%E2%80%9D%5Btiab%5D&ac=no&sort=relevance) |
| #4 | Search: #1 OR #2 OR #3 | [381,859](https://pubmed.ncbi.nlm.nih.gov/?term=%231+OR+%232+OR+%233&ac=no&sort=relevance) |
| #5 | Search: "Body Weight"[Mesh:NoExp] OR bmi[tiab] OR "body mass index"[tiab] OR "bodymass index"[tiab] OR "bodymassindex"[tiab] OR "body massindex"[tiab] OR "quetelet index"[tiab] | [439,382](https://pubmed.ncbi.nlm.nih.gov/?term=%E2%80%9CBody+Weight%E2%80%9D%5BMesh%3ANoExp%5D+OR+bmi%5Btiab%5D+OR+%E2%80%9Cbody+mass+index%E2%80%9D%5Btiab%5D+OR+%E2%80%9Cbodymass+index%E2%80%9D%5Btiab%5D+OR+%E2%80%9Cbodymassindex%E2%80%9D%5Btiab%5D+OR+%E2%80%9Cbody+massindex%E2%80%9D%5Btiab%5D+OR+%E2%80%9Cquetelet+index%E2%80%9D%5Btiab%5D&ac=no&sort=relevance) |
| #6 | Search: "Metabolic Syndrome"[Mesh:NoExp] OR "Waist Circumference"[Mesh:NoExp] OR "Waist-Hip Ratio"[Mesh:NoExp] OR (waist[tiab] AND circumference[tiab]) OR (waist[tiab] AND hip[tiab] AND ratio[tiab]) OR "metabolic syndrome"[tiab] OR "general population"[tiab] OR "normal population"[tiab] OR "high risk"[tiab] OR "intermediate risk"[tiab] OR "low risk"[tiab] OR (insulin[tiab] AND resist*[tiab]) OR "class I"[tiab] OR "class 1"[tiab] OR "class II"[tiab] OR "class 2"[tiab] OR "class III"[tiab] OR "class 3"[tiab] OR (weight*[tiab] AND (loss[tiab] OR lost[tiab] OR reduc*[tiab] OR change[tiab] OR gain[tiab] OR fluctuation[tiab] OR variation[tiab] OR trajectory[tiab] OR body[tiab] OR related[tiab])) | 1,342,973 |
| #7 | Search: "Glucose Intolerance"[mh] OR "Prediabetic State"[mh] OR prediabetes[tiab] OR "pre diabetes"[tiab] OR prediabetic[tiab] OR (impair*[tiab] AND glucose[tiab] AND tolerance[tiab]) OR (diabetes[tiab] AND (chemical[tiab] OR potential[tiab] OR latent[tiab])) | [96,745](https://pubmed.ncbi.nlm.nih.gov/?term=%E2%80%9CGlucose+Intolerance%E2%80%9D%5Bmh%5D+OR+%E2%80%9CPrediabetic+State%E2%80%9D%5Bmh%5D+OR+prediabetes%5Btiab%5D+OR+%E2%80%9Cpre+diabetes%E2%80%9D%5Btiab%5D+OR+prediabetic%5Btiab%5D+OR+%28impair%2A%5Btiab%5D+AND+glucose%5Btiab%5D+AND+tolerance%5Btiab%5D%29+OR+%28diabetes%5Btiab%5D+AND+%28chemical%5Btiab%5D+OR+potential%5Btiab%5D+OR+latent%5Btiab%5D%29%29&ac=no&sort=relevance) |
| #8 | Search: "Diabetes Mellitus, Type 2"[mh] OR T2DM[tiab] OR T2D[tiab] OR DM2[tiab] OR "DM 2"[tiab] OR NIDDM[tiab] OR (diabetes[tiab] AND ("type 2"[tiab] OR type2[tiab] OR "type II"[tiab] OR typeII[tiab])) | [205,500](https://pubmed.ncbi.nlm.nih.gov/?term=%E2%80%9CDiabetes+Mellitus%2C+Type+2%E2%80%9D%5Bmh%5D+OR+T2DM%5Btiab%5D+OR+T2D%5Btiab%5D+OR+DM2%5Btiab%5D+OR+%E2%80%9CDM+2%E2%80%9D%5Btiab%5D+OR+NIDDM%5Btiab%5D+OR+%28diabetes%5Btiab%5D+AND+%28%E2%80%9Ctype+2%E2%80%9D%5Btiab%5D+OR+type2%5Btiab%5D+OR+%E2%80%9Ctype+II%E2%80%9D%5Btiab%5D+OR+typeII%5Btiab%5D%29%29&ac=no&sort=relevance) |
| #9 | Search: "Cardiovascular Diseases"[Mesh:NoExp] OR "Coronary Artery Disease"[Mesh:NoExp] OR "Diabetic Angiopathies"[mh] OR "Thromboembolism"[mh] OR "Myocardial Infarction"[mh] OR CVD[tiab] OR angiocardiopathy[tiab] OR thromboembolism[tiab] OR thrombosis[tiab] OR VTE[tiab] OR embolism[tiab] OR ((ischemic[tiab] OR ischaemic[tiab] OR coronary[tiab]) AND "heart disease"[tiab]) OR "coronary artery disease"[tiab] OR ((cardiovascular[tiab] OR angiocardiovascular[tiab] OR CV[tiab]) AND (disease[tiab] OR complication*[tiab] OR disorder*[tiab] OR event*[tiab] OR endpoint*[tiab] OR outcome*[tiab])) OR (diabetic[tiab] AND angiopathy[tiab]) | [960,613](https://pubmed.ncbi.nlm.nih.gov/?term=%E2%80%9CCardiovascular+Diseases%E2%80%9D%5BMesh%3ANoExp%5D+OR+%E2%80%9CCoronary+Artery+Disease%E2%80%9D%5BMesh%3ANoExp%5D+OR+%E2%80%9CDiabetic+Angiopathies%E2%80%9D%5Bmh%5D+OR+%E2%80%9CThromboembolism%E2%80%9D%5Bmh%5D+OR+%E2%80%9CMyocardial+Infarction%E2%80%9D%5Bmh%5D+OR+CVD%5Btiab%5D+OR+angiocardiopathy%5Btiab%5D+OR+thromboembolism%5Btiab%5D+OR+thrombosis%5Btiab%5D+OR+VTE%5Btiab%5D+OR+embolism%5Btiab%5D+OR+%28%28ischemic%5Btiab%5D+OR+ischaemic%5Btiab%5D+OR+coronary%5Btiab%5D%29+AND+%E2%80%9Cheart+disease%E2%80%9D%5Btiab%5D%29+OR+%E2%80%9Ccoronary+artery+disease%E2%80%9D%5Btiab%5D+OR+%28%28cardiovascular%5Btiab%5D+OR+angiocardiovascular%5Btiab%5D+OR+CV%5Btiab%5D%29+AND+%28disease%5Btiab%5D+OR+complication%2A%5Btiab%5D+OR+disorder%2A%5Btiab%5D+OR+event%2A%5Btiab%5D+OR+endpoint%2A%5Btiab%5D+OR+outcome%2A%5Btiab%5D%29%29+OR+%28diabetic%5Btiab%5D+AND+angiopathy%5Btiab%5D%29&ac=no&sort=relevance) |
| #10 | Search: "Heart Diseases"[Mesh:NoExp] OR "Heart Aneurysm"[mh] OR "Heart Failure"[mh] OR "Myocardial Ischemia"[Mesh:NoExp] OR "Stroke Volume"[mh] OR "heart disease"[tiab] OR "heart aneurysm"[tiab] OR "heart failure"[tiab] OR HfpEF[tiab] | [479,785](https://pubmed.ncbi.nlm.nih.gov/?term=%E2%80%9CHeart+Diseases%E2%80%9D%5BMesh%3ANoExp%5D+OR+%E2%80%9CHeart+Aneurysm%E2%80%9D%5Bmh%5D+OR+%E2%80%9CHeart+Failure%E2%80%9D%5Bmh%5D+OR+%E2%80%9CMyocardial+Ischemia%E2%80%9D%5BMesh%3ANoExp%5D+OR+%E2%80%9CStroke+Volume%E2%80%9D%5Bmh%5D+OR+%E2%80%9Cheart+disease%E2%80%9D%5Btiab%5D+OR+%E2%80%9Cheart+aneurysm%E2%80%9D%5Btiab%5D+OR+%E2%80%9Cheart+failure%E2%80%9D%5Btiab%5D+OR+HfpEF%5Btiab%5D&ac=no&sort=relevance) |
| #11 | Search: "Acute Coronary Syndrome"[mh] OR "Angina Pectoris"[mh] OR "acute coronary syndrome*"[tiab] OR (ACS[tiab] AND (acute[tiab] OR coronary[tiab] or syndrome*[tiab])) OR (angina[tiab] AND unstable[tiab]) OR ((myocardial[tiab] OR heart[tiab]) AND infarct*[tiab]) | [277,029](https://pubmed.ncbi.nlm.nih.gov/?term=%E2%80%9CAcute+Coronary+Syndrome%E2%80%9D%5Bmh%5D+OR+%E2%80%9CAngina+Pectoris%E2%80%9D%5Bmh%5D+OR+%E2%80%9Cacute+coronary+syndrome%2A%E2%80%9D%5Btiab%5D+OR+%28ACS%5Btiab%5D+AND+%28acute%5Btiab%5D+OR+coronary%5Btiab%5D+or+syndrome%2A%5Btiab%5D%29%29+OR+%28angina%5Btiab%5D+AND+unstable%5Btiab%5D%29+OR+%28%28myocardial%5Btiab%5D+OR+heart%5Btiab%5D%29+AND+infarct%2A%5Btiab%5D%29&ac=no&sort=relevance) |
| #12 | Search: "Stroke"[mh] OR "Ischemic Attack, Transient"[mh] OR stroke[tiab] OR apoplexy[tiab] OR apoplexia[tiab] OR (cerebrovascular[tiab] AND (accident[tiab] OR accidents[tiab] OR lesion[tiab] OR arrest[tiab] OR failure[tiab] OR injury[tiab] OR insufficiency[tiab] OR insult[tiab])) OR ((cerebro[tiab] OR cerebral[tiab] OR brain[tiab] OR cerebrum[tiab]) AND (vasculopathy[tiab] OR accident[tiab] OR attack[tiab] OR insult*[tiab] OR insufficiency[tiab])) OR (seizure[tiab] AND (ischaemic[tiab] OR ischemic[tiab])) OR (CVA[tiab] AND (cerebrovascular[tiab] OR attack[tiab])) OR (TIA[tiab] AND (transient[tiab] OR ischaemic[tiab] OR ischemic[tiab] OR attack[tiab])) | [343,957](https://pubmed.ncbi.nlm.nih.gov/?term=%E2%80%9CStroke%E2%80%9D%5Bmh%5D+OR+%E2%80%9CIschemic+Attack%2C+Transient%E2%80%9D%5Bmh%5D+OR+stroke%5Btiab%5D+OR+apoplexy%5Btiab%5D+OR+apoplexia%5Btiab%5D+OR+%28cerebrovascular%5Btiab%5D+AND+%28accident%5Btiab%5D+OR+accidents%5Btiab%5D+OR+lesion%5Btiab%5D+OR+arrest%5Btiab%5D+OR+failure%5Btiab%5D+OR+injury%5Btiab%5D+OR+insufficiency%5Btiab%5D+OR+insult%5Btiab%5D%29%29+OR+%28%28cerebro%5Btiab%5D+OR+cerebral%5Btiab%5D+OR+brain%5Btiab%5D+OR+cerebrum%5Btiab%5D%29+AND+%28vasculopathy%5Btiab%5D+OR+accident%5Btiab%5D+OR+attack%5Btiab%5D+OR+insult%2A%5Btiab%5D+OR+insufficiency%5Btiab%5D%29%29+OR+%28seizure%5Btiab%5D+AND+%28ischaemic%5Btiab%5D+OR+ischemic%5Btiab%5D%29%29+OR+%28CVA%5Btiab%5D+AND+%28cerebrovascular%5Btiab%5D+OR+attack%5Btiab%5D%29%29+OR+%28TIA%5Btiab%5D+AND+%28transient%5Btiab%5D+OR+ischaemic%5Btiab%5D+OR+ischemic%5Btiab%5D+OR+attack%5Btiab%5D%29%29&ac=no&sort=relevance) |
| #13 | Search: "Musculoskeletal Diseases"[mh] OR (musculoskeletal[tiab] AND (disorder[tiab] OR disorders[tiab])) | [1,113,729](https://pubmed.ncbi.nlm.nih.gov/?term=%E2%80%9CMusculoskeletal+Diseases%E2%80%9D%5Bmh%5D+OR+%28musculoskeletal%5Btiab%5D+AND+%28disorder%5Btiab%5D+OR+disorders%5Btiab%5D%29%29&ac=no&sort=relevance) |
| #14 | Search: "Arthroplasty, Replacement, Knee"[mh] OR (knee[tiab] AND (replace*[tiab] OR arthroplasty[tiab])) | [42,555](https://pubmed.ncbi.nlm.nih.gov/?term=%E2%80%9CArthroplasty%2C+Replacement%2C+Knee%E2%80%9D%5Bmh%5D+OR+%28knee%5Btiab%5D+AND+%28replace%2A%5Btiab%5D+OR+arthroplasty%5Btiab%5D%29%29&ac=no&sort=relevance) |
| #15 | Search: "Sleep Apnea, Obstructive"[mh] OR ((sleep[tiab] OR nocturnal[tiab]) AND (apnea[tiab] OR apnoea[tiab])) OR (OSA[tiab] AND (obstructive[tiab] OR sleep[tiab] OR apnea[tiab] OR apnoea[tiab])) | [45,013](https://pubmed.ncbi.nlm.nih.gov/?term=%E2%80%9CSleep+Apnea%2C+Obstructive%E2%80%9D%5Bmh%5D+OR+%28%28sleep%5Btiab%5D+OR+nocturnal%5Btiab%5D%29+AND+%28apnea%5Btiab%5D+OR+apnoea%5Btiab%5D%29%29+OR+%28OSA%5Btiab%5D+AND+%28obstructive%5Btiab%5D+OR+sleep%5Btiab%5D+OR+apnea%5Btiab%5D+OR+apnoea%5Btiab%5D%29%29&ac=no&sort=relevance) |
| #16 | Search: #7 OR #8 OR #9 OR #10 OR #11 OR #12 OR #13 OR #14 OR #15 | [2,900,590](https://pubmed.ncbi.nlm.nih.gov/?term=%237+OR+%238+OR+%239+OR+%2310+OR+%2311+OR+%2312+OR+%2313+OR+%2314+OR+%2315&ac=no&sort=relevance) |
| #17 | Search: "Risk Assessment"[mh] OR "Risk Factors"[mh] OR "Forecasting"[mh] OR "Clinical Decision Rules"[mh] (risk[tiab] AND (predict*[tiab] OR equation[tiab] OR equations[tiab] OR score[tiab] OR scores[tiab] OR scale[tiab] OR scales[tiab])) | [197,856](https://pubmed.ncbi.nlm.nih.gov/?term=%E2%80%9CRisk+Assessment%E2%80%9D%5Bmh%5D+OR+%E2%80%9CRisk+Factors%E2%80%9D%5Bmh%5D+OR+%E2%80%9CForecasting%E2%80%9D%5Bmh%5D+OR+%E2%80%9CClinical+Decision+Rules%E2%80%9D%5Bmh%5D+%28risk%5Btiab%5D+AND+%28predict%2A%5Btiab%5D+OR+equation%5Btiab%5D+OR+equations%5Btiab%5D+OR+score%5Btiab%5D+OR+scores%5Btiab%5D+OR+scale%5Btiab%5D+OR+scales%5Btiab%5D%29%29&ac=no&sort=relevance) |
| #18 | Search: "Population"[mh] OR "population based"[tiab] OR (population[tiab] AND (large[tiab] OR sample[tiab] OR level[tiab])) OR "whole country"[tiab] OR national[tiab] OR international[tiab] OR nationwide[tiab] OR "nation wide"[tiab] OR "large scale"[tiab] OR regional[tiab] OR "region wide"[tiab] OR (representative[tiab] AND (study[tiab] OR sample[tiab] OR population[tiab])) | [1,808,384](https://pubmed.ncbi.nlm.nih.gov/?term=%E2%80%9CPopulation%E2%80%9D%5Bmh%5D+OR+%E2%80%9Cpopulation+based%E2%80%9D%5Btiab%5D+OR+%28population%5Btiab%5D+AND+%28large%5Btiab%5D+OR+sample%5Btiab%5D+OR+level%5Btiab%5D%29%29+OR+%E2%80%9Cwhole+country%E2%80%9D%5Btiab%5D+OR+national%5Btiab%5D+OR+international%5Btiab%5D+OR+nationwide%5Btiab%5D+OR+%E2%80%9Cnation+wide%E2%80%9D%5Btiab%5D+OR+%E2%80%9Clarge+scale%E2%80%9D%5Btiab%5D+OR+regional%5Btiab%5D+OR+%E2%80%9Cregion+wide%E2%80%9D%5Btiab%5D+OR+%28representative%5Btiab%5D+AND+%28study%5Btiab%5D+OR+sample%5Btiab%5D+OR+population%5Btiab%5D%29%29&ac=no&sort=relevance) |
| #19 | Search: "Incidence"[mh] OR "Proportional Hazards Models"[mh] OR incidence[tiab] OR incident[tiab] OR ("area under"[tiab] AND curve[tiab]) OR AUC[tiab] OR hazard[tiab] OR rate[tiab] OR risk[tiab] OR odds[tiab] OR likelihood[tiab] OR probability[tiab] | [5,089,326](https://pubmed.ncbi.nlm.nih.gov/?term=%E2%80%9CIncidence%E2%80%9D%5Bmh%5D+OR+%E2%80%9CProportional+Hazards+Models%E2%80%9D%5Bmh%5D+OR+incidence%5Btiab%5D+OR+incident%5Btiab%5D+OR+%28%E2%80%9Carea+under%E2%80%9D%5Btiab%5D+AND+curve%5Btiab%5D%29+OR+AUC%5Btiab%5D+OR+hazard%5Btiab%5D+OR+rate%5Btiab%5D+OR+risk%5Btiab%5D+OR+odds%5Btiab%5D+OR+likelihood%5Btiab%5D+OR+probability%5Btiab%5D&ac=no&sort=relevance) |
| #20 | Search: "Europe"[mh] OR "North America"[Mesh:NoExp] OR "Canada"[mh] OR "United States"[mh] OR "Australia"[mh] OR Europe*[tiab] EU OR Austria*[tiab] OR Belgium[tiab] OR Belgian[tiab] OR Brussels[tiab] OR Flanders[tiab] OR Wallonia[tiab] OR Benelux[tiab] OR "Channel Islands"[tiab] OR Guernsey[tiab] OR Jersey[tiab] OR France[tiab] OR French[tiab] OR Corsica*[tiab] OR German*[tiab] OR (German[tiab] AND Republic[tiab]) OR Prussia*[tiab] OR Ireland[tiab] OR Irish[tiab] OR "Isle of Man"[tiab] OR Liechtenstein[tiab] OR Luxembourg[tiab] OR Monaco[tiab] OR Netherlands[tiab] OR Dutch[tiab] OR Scandinavia*[tiab] OR Denmark[tiab] OR Danish[tiab] OR "Faroe Islands"[tiab] OR Finland[tiab] OR Finnish[tiab] OR Aland[tiab] OR Greenland[tiab] OR Iceland*[tiab] OR Norway[tiab] OR Norwegian[tiab] OR Svalbard[tiab] OR Spitsbergen[tiab] OR "Jan Mayen"[tiab] OR Sweden[tiab] OR Swedish[tiab] OR Switzerland[tiab] OR Swiss[tiab] OR "United Kingdom"[tiab] OR UK[tiab] OR "Great Britain"[tiab] OR British[tiab] OR "Northern Ireland"[tiab] OR England[tiab] OR English[tiab] OR Scotland[tiab] OR Scottish[tiab] OR Wales[tiab] OR Welsh[tiab] OR Andorra[tiab] OR Gibraltar[tiab] OR Greece[tiab] OR Greek[tiab] OR Italy[tiab] OR Italian[tiab] OR Aosta[tiab] OR "Friuli Venezia"[tiab] OR Sardinia*[tiab] OR Sicily[tiab] OR Sicilian[tiab] OR Trentino[tiab] OR "South Tyrol"[tiab] OR Malta[tiab] OR Maltan[tiab] OR Portugal[tiab] OR Portuguese[tiab] OR Azores[tiab] OR Madeira[tiab] OR "San Marino"[tiab] OR Spain[tiab] OR Spanish[tiab] OR "Balearic Islands"[tiab] OR "Basque Country"[tiab] OR "Canary Islands"[tiab] OR Catalonia*[tiab] OR Galicia*[tiab] OR Navarre[tiab] OR Valencian[tiab] OR "Vatican City"[tiab] OR Albania*[tiab] OR Armenia*[tiab] OR Azerbaijan[tiab] OR "Nagorno Karabakh"[tiab] OR "Balkan Peninsula"[tiab] OR "Baltic States"[tiab] OR Estonia*[tiab] OR Latvia*[tiab] OR Lithuania*[tiab] OR Belarus*[tiab] OR Bosnia*[tiab] OR Herzegovina*[tiab] OR Herzegowina*[tiab] OR Srpska[tiab] OR Yugoslavia*[tiab] OR Bulgaria*[tiab] OR Croatia*[tiab] OR "Czech Republic"[tiab] OR Georgia*[tiab] OR Abkhazia[tiab] OR "South Ossetia"[tiab] OR Hungary[tiab] OR Hungarian[tiab] OR Kosovo*[tiab] OR Moldova*[tiab] OR Montenegro[tiab] OR Poland[tiab] OR Polish[tiab] OR Macedonia*[tiab] OR Romania*[tiab] OR Serbia*[tiab] OR Vojvodina[tiab] OR Slovakia*[tiab] OR Slovenia*[tiab] OR Ukrain*[tiab] OR Crimea*[tiab] OR America[tiab] OR American[tiab] OR "United States"[tiab] OR USA[tiab] OR "U S A"[tiab] OR Alabama[tiab] OR Alaska[tiab] OR Arizona[tiab] OR Arkansas[tiab] OR California[tiab] OR Colorado[tiab] OR Connecticut[tiab] OR Delaware[tiab] OR "District of Columbia"[tiab] OR Florida[tiab] OR Georgia[tiab] OR Hawaii[tiab] OR Idaho[tiab] OR Illinois[tiab] OR Indiana[tiab] OR Iowa[tiab] OR Kansas[tiab] OR Kentucky[tiab] OR Louisiana[tiab] OR Maine[tiab] OR Maryland[tiab] OR Massachusetts[tiab] OR Michigan[tiab] OR Minnesota[tiab] OR Mississippi[tiab] OR Missouri[tiab] OR Montana[tiab] OR Nebraska[tiab] OR Nevada[tiab] OR "New Hampshire"[tiab] OR "New Jersey"[tiab] OR "New Mexico"[tiab] OR "New York"[tiab] OR "North Carolina"[tiab] OR "North Dakota"[tiab] OR Ohio[tiab] OR Oklahoma[tiab] OR Oregon[tiab] OR Pennsylvania[tiab] OR "Rhode Island"[tiab] OR "South Carolina"[tiab] OR "South Dakota"[tiab] OR Tennessee[tiab] OR Texas[tiab] OR Utah[tiab] OR Vermont[tiab] OR Virginia[tiab] OR Washington[tiab] OR Wisconsin[tiab] OR Wyoming[tiab] OR "New England"[tiab] OR "appalachian region"[tiab] OR "great lakes region"[tiab] OR "mid atlantic region"[tiab] OR Canada[tiab] OR Canadian[tiab] OR Alberta*[tiab] OR "British Columbia"[tiab] OR Manitoba*[tiab] OR "New Brunswick"[tiab] OR Newfoundland[tiab] OR Labrador[tiab] OR "Northwest Territories"[tiab] OR "Nova Scotia"[tiab] OR Nunavut[tiab] OR Ontario[tiab] OR "Prince Edward Island"[tiab] OR Quebec[tiab] OR Saskatchewan[tiab] OR Yukon[tiab] OR Australia*[tiab] OR (NSW[tiab] AND (New[tiab] OR South[tiab])) OR "Northern Territory"[tiab] OR Queensland[tiab] OR Tasmania*[tiab] OR Victoria*[tiab] | [2,541,954](https://pubmed.ncbi.nlm.nih.gov/?term=longqueryb73a24e23f6a3b261add&ac=no&sort=relevance) |
| #21 | Search: [1 OR (#5 AND #6]) AND #16 AND #17 AND #18 AND #19 AND #20 | [1,165](https://pubmed.ncbi.nlm.nih.gov/?term=%28%234%C2%A0OR+%28%235%C2%A0AND%C2%A0%236%29%29+AND%C2%A0%2316%C2%A0AND%C2%A0%2317%C2%A0AND%C2%A0%2318%C2%A0AND%C2%A0%2319%C2%A0AND%C2%A0%2320&ac=no&sort=relevance) |
| #22 | Search: (normal[tiab] AND glucose[tiab] AND tolerance[tiab]) OR (NGT[tiab] AND (normal[tiab] OR glucose[tiab] OR tolerance[tiab])) | [14,989](https://pubmed.ncbi.nlm.nih.gov/?term=%28normal%5Btiab%5D+AND+glucose%5Btiab%5D+AND+tolerance%5Btiab%5D%29+OR+%28NGT%5Btiab%5D+AND+%28normal%5Btiab%5D+OR+glucose%5Btiab%5D+OR+tolerance%5Btiab%5D%29%29&ac=no&sort=relevance) |
| #23 | Search: (#1 OR #2 OR (#5 AND #6)) AND [2 OR #22] AND #8 AND #18 AND #19 AND #20 | [480](https://pubmed.ncbi.nlm.nih.gov/?term=%28%231%C2%A0OR%C2%A0%232%C2%A0OR+%28%235%C2%A0AND%C2%A0%236%29%29+AND+%28%237%C2%A0OR%C2%A0%2322%29+AND%C2%A0%238%C2%A0AND%C2%A0%2318%C2%A0AND%C2%A0%2319%C2%A0AND%C2%A0%2320&ac=no&sort=relevance) |
| #24 | Search: (#1 OR (#5 AND #6)) AND [3 OR #22] AND [4 OR #10 OR #11 OR #12] AND #18 AND #19 AND #20 | [426](https://pubmed.ncbi.nlm.nih.gov/?term=%28%231%C2%A0OR+%28%235%C2%A0AND%C2%A0%236%29%29+AND+%28%238%C2%A0OR%C2%A0%2322%29+AND+%28%239%C2%A0OR%C2%A0%2310%C2%A0OR%C2%A0%2311%C2%A0OR%C2%A0%2312%29+AND%C2%A0%2318%C2%A0AND%C2%A0%2319%C2%A0AND%C2%A0%2320&ac=no&sort=relevance) |
| #25 | Search: "Hypertension"[mh] OR hypertension[tiab] OR hypertensive[tiab] OR hypertensives[tiab] OR (high[tiab] AND "blood pressure"[tiab]) | [558,802](https://pubmed.ncbi.nlm.nih.gov/?term=%E2%80%9CHypertension%E2%80%9D%5Bmh%5D+OR+hypertension%5Btiab%5D+OR+hypertensive%5Btiab%5D+OR+hypertensives%5Btiab%5D+OR+%28high%5Btiab%5D+AND+%E2%80%9Cblood+pressure%E2%80%9D%5Btiab%5D%29&ac=no&sort=relevance) |
| #26 | Search: "Dyslipidemias"[mh] OR dyslipidemia[tiab] OR dyslipidemias[tiab] OR dyslipaemia[tiab] OR dyslipemia[tiab] OR dyslipidaemia[tiab] OR dyslipidaemias[tiab] OR lipidaemia[tiab] OR lipidemia[tiab] | [105,607](https://pubmed.ncbi.nlm.nih.gov/?term=%E2%80%9CDyslipidemias%E2%80%9D%5Bmh%5D+OR+dyslipidemia%5Btiab%5D+OR+dyslipidemias%5Btiab%5D+OR+dyslipaemia%5Btiab%5D+OR+dyslipemia%5Btiab%5D+OR+dyslipidaemia%5Btiab%5D+OR+dyslipidaemias%5Btiab%5D+OR+lipidaemia%5Btiab%5D+OR+lipidemia%5Btiab%5D&ac=no&sort=relevance) |
| #27 | Search: #8 OR #9 OR #15 OR #25 OR #26 | [1,626,942](https://pubmed.ncbi.nlm.nih.gov/?term=%238%C2%A0OR%C2%A0%239%C2%A0OR%C2%A0%2315%C2%A0OR%C2%A0%2325%C2%A0OR%C2%A0%2326&ac=no&sort=relevance) |
| #28 | Search: "Mortality"[mh] OR "Mortality"[Subheading] OR "Survival Analysis"[mh] OR mortality[tiab] OR death[tiab] OR deaths[tiab] OR "case fatality rate"[tiab] | [2,043,006](https://pubmed.ncbi.nlm.nih.gov/?term=%E2%80%9CMortality%E2%80%9D%5Bmh%5D+OR+%E2%80%9CMortality%E2%80%9D%5BSubheading%5D+OR+%E2%80%9CSurvival+Analysis%E2%80%9D%5Bmh%5D+OR+mortality%5Btiab%5D+OR+death%5Btiab%5D+OR+deaths%5Btiab%5D+OR+%E2%80%9Ccase+fatality+rate%E2%80%9D%5Btiab%5D&ac=no&sort=relevance) |
| #29 | Search: (#1 OR #2 OR (#3 AND #27)) AND [5 OR #6] AND [4 OR #10 OR #11 OR #12 OR #14] AND #18 AND #20 AND #28 | [956](https://pubmed.ncbi.nlm.nih.gov/?term=%28%231%C2%A0OR%C2%A0%232%C2%A0OR+%28%233%C2%A0AND%C2%A0%2327%29%29+AND+%28%235%C2%A0OR%C2%A0%236%29+AND+%28%239%C2%A0OR%C2%A0%2310%C2%A0OR%C2%A0%2311%C2%A0OR%C2%A0%2312%C2%A0OR%C2%A0%2314%29+AND%C2%A0%2318%C2%A0AND%C2%A0%2320%C2%A0AND%C2%A0%2328&ac=no&sort=relevance) |
| #30 | Search: "english abstract"[Publication Type] | [1,482,778](https://pubmed.ncbi.nlm.nih.gov/?term=%22english+abstract%22%5BPublication+Type%5D&ac=no&sort=relevance) |
| #31 | Search: #29 NOT #30 | [919](https://pubmed.ncbi.nlm.nih.gov/?term=%2329+NOT+%2330&ac=no&sort=relevance) |
| #32 | Search: #21 OR #23 OR #24 OR #31 | [2,513](https://pubmed.ncbi.nlm.nih.gov/?term=%2321%C2%A0OR%C2%A0%2323%C2%A0OR%C2%A0%2324%C2%A0OR%C2%A0%2331&ac=no&sort=relevance) |
| #33 | Search: "Cohort Studies"[mh] OR "Case-Control Studies"[mh] OR "Registries"[mh] OR "Validation Study"[mh] OR "Surveys and Questionnaires"[mh] OR cohort[tiab] OR "case cohort"[tiab] OR "case base"[tiab] OR "case control"[tiab] OR register[tiab] OR registry[tiab] OR registries[tiab] OR validat*[tiab] OR longitudinal[tiab] OR "controlled study"[tiab] OR questionnaire[tiab] OR questionnaires[tiab] | [4,293,215](https://pubmed.ncbi.nlm.nih.gov/?term=%E2%80%9CCohort+Studies%E2%80%9D%5Bmh%5D+OR+%E2%80%9CCase-Control+Studies%E2%80%9D%5Bmh%5D+OR+%E2%80%9CRegistries%E2%80%9D%5Bmh%5D+OR+%E2%80%9CValidation+Study%E2%80%9D%5Bmh%5D+OR+%E2%80%9CSurveys+and+Questionnaires%E2%80%9D%5Bmh%5D+OR+cohort%5Btiab%5D+OR+%E2%80%9Ccase+cohort%E2%80%9D%5Btiab%5D+OR+%E2%80%9Ccase+base%E2%80%9D%5Btiab%5D+OR+%E2%80%9Ccase+control%E2%80%9D%5Btiab%5D+OR+register%5Btiab%5D+OR+registry%5Btiab%5D+OR+registries%5Btiab%5D+OR+validat%2A%5Btiab%5D+OR+longitudinal%5Btiab%5D+OR+%E2%80%9Ccontrolled+study%E2%80%9D%5Btiab%5D+OR+questionnaire%5Btiab%5D+OR+questionnaires%5Btiab%5D&ac=no&sort=relevance) |
| #34 | Search: #32 AND #33 | [1,755](https://pubmed.ncbi.nlm.nih.gov/?term=%2332+AND+%2333&ac=no&sort=relevance) |
| #35 | Search: "comment"[Publication Type] OR "editorial"[Publication Type] OR "letter"[Publication Type] | [1,941,945](https://pubmed.ncbi.nlm.nih.gov/?term=%22comment%22%5BPublication+Type%5D+OR+%22editorial%22%5BPublication+Type%5D+OR+%22letter%22%5BPublication+Type%5D&ac=no&sort=relevance) |
| #36 | Search: #34 NOT #35 | [1,749](https://pubmed.ncbi.nlm.nih.gov/?term=%2334+NOT+%2335&ac=no&sort=relevance) |
| #37 | Search: ("2010/01/01"[Date - Publication] : "3000"[Date - Publication]) | [12,377,278](https://pubmed.ncbi.nlm.nih.gov/?term=%28%222010%2F01%2F01%22%5BDate+-+Publication%5D+%3A+%223000%22%5BDate+-+Publication%5D%29&ac=no&sort=relevance) |
| #38 | Search: #36 AND #37 | [1,224](https://pubmed.ncbi.nlm.nih.gov/?term=%2336+AND+%2337&ac=no&sort=relevance) |

# Table S4. Data extraction categories

| **Question** | **Information category** |  |
| --- | --- | --- |
| All | Study characteristics | Publication year, author, country, title, region, and study type |
| Review question 1 ^a^ | Cohort characteristics | Size, BMI range, background, comorbidities, baseline characteristics (used in risk equation) |
|  | Risk equation details | Risk predictor selection rationale, validation, timeframe, stats, equation performance, equation comparisons |
|  | Outcome | Risk of developing, definition of outcome, method of reporting outcome |
| Review questions 2 and 3 ^b, c^ | Cohort characteristics | Size, BMI range, average age, male/female, glucose tolerance |
|  | Outcomes | Risk of developing, absolute risk, hazard ratio, odds ratio, definition of outcome, descriptive conclusion |
| Review question 4 ^d^ | Cohort characteristics | Size, BMI range, average age, male/female, comorbidities |
|  | Outcome | Risk of mortality from, absolute risk, hazard ratio, odds ratio, definition of mortality, method used to calculate risk, descriptive conclusion |
| *ACS* acute coronary syndrome, *BMI* body mass index, *CV* cardiovascular, *CVD* cardiovascular disease, *MI* myocardial infarction, *NGT* normal glucose tolerance, *T2D* type 2 diabetes, *TIA* transient ischemic attack  ^a^ Review question 1: What risk equations have been published to calculate the risk of people with obesity ^e^ or overweight ^h^ with at least one weight-related comorbidity ^f^ developing at least one additional comorbidity ^g^?  ^b^ Review question 2: What is the risk of an adult with a BMI ≥30 and NGT or prediabetes developing T2D?  ^c^ Review question 3: What is the risk of an adult with a BMI ≥40 and NGT or T2D experiencing a CV event?  ^d^ Review question 4: What is the risk of mortality arising from knee surgery, stroke, and acute CV events in people who are obese ^e^ or overweight ^h^ with at least one weight-related comorbidity ^f^?  ^e^ Adults with a BMI ≥30 kg/m^2^; ^f^ T2D, hypertension, dyslipidemia, obstructive sleep apnea, or CVD; ^g^ Prediabetes, T2D, CVD, heart disease, ACS (including MI and unstable angina), stroke (including TIA), musculoskeletal disorders, knee replacement, sleep apnea; ^h^ BMI ≥25 kg/m^2^ | | |

# Table S5. The adapted Newcastle-Ottawa Scale [6]

| **Selection**  **Maximum 8* ^a^** | **Comparability**  **Maximum 2*** | **Outcome**  **Maximum 3*** | **Validation**  **Maximum 2*** |
| --- | --- | --- | --- |
| Ascertainment of exposure (i.e., measurement of BMI) | Comparability of the exposed (i.e., overweight and obese) and non-exposed (i.e.,  BMI 18.5–24.9) populations, based on study design and analysis | Measurement of outcome | Validation of the risk equation or assessment method |
| Representativeness of the cohort |  | Measurement of  follow-up |  |

*BMI* body mass index

Each study was assessed and awarded stars based on the three Newcastle-Ottawa Scale criteria plus an additional fourth validation criteria. A maximum of 15 stars was possible. The addition validation criteria questions were: Was the equation validated: internally (a,*) externally (b,**) or no mention (c ), or compared risk equations (d,*)

^a^ ; * indicates the star awarded

# Table S6. Studies excluded following the full-text screening

| **Author(s)** | **Title** | **Year** | **Reason for exclusion** |
| --- | --- | --- | --- |
| M. Abd Alamir, V. Radulescu, M. Goyfman, E. R. Mohler, 3rd, Y. L. Gao, and M. J. Budoff | Prevalence and correlates of mitral annular calcification in adults with chronic kidney disease: results from CRIC study | 2015 | Confounding variables |
| M. Acevedo, G. Valentino, V. Kramer, M. J. Bustamante, M. Adasme, L. Orellana, F. Baraona, and C. Navarrete | Evaluation the American College of Cardiology and American Heart Association Predictive score for cardiovascular diseases | 2017 | Country |
| S. Acharjee, M. T. Roe, E. A. Amsterdam, D. Holmes, and W. E. Boden | Prevalence of low levels of high-density lipoprotein cholesterol (HDL-C) at presentation in acute coronary syndromes (ACS) and its relation to in-hospital mortality: results from the NCDR^®^ | 2011 | Abstract only |
| T. D. Adams, L. E. Davidson, S. E. Litwin, R. L. Kolotkin, M. J. LaMonte, R. C. Pendleton, M. B. Strong, R. Vinik, N. A. Wanner, P. N. Hopkins, R. E. Gress, J. M. Walker, T. V. Cloward, R. T. Nuttall, A. Hammoud, J. L. Greenwood, R. D. Crosby, R. McKinlay, S. C. Simper, S. C. Smith, and S. C. Hunt | Health benefits of gastric bypass surgery after 6 years | 2012 | Population |
| S. Adriouch, C. Julia, E. Kesse-Guyot, P. Ducrot, S. Péneau, C. Méjean, K. E. Assmann, M. Deschasaux, S. Hercberg, M. Touvier, and L. K. Fezeu | Association between a dietary quality index based on the food standard agency nutrient profiling system and cardiovascular disease risk among French adults | 2017 | Risk equation not reported |
| E. J. Aguiar, P. J. Morgan, C. E. Collins, R. C. Plotnikoff, M. D. Young, and R. Callister | The PULSE (Prevention Using LifeStyle Education) trial protocol: a randomised controlled trial of a Type 2 Diabetes Prevention programme for men | 2014 | Risk not calculated |
| A. Ahmad Kiadaliri, P. M. Clarke, U. G. Gerdtham, P. M. Nilsson, B. Eliasson, S. Gudbjörnsdottir, and K. Steen Carlsson | Predicting changes in risk factors in type 2 diabetes in the post-UKPDS era: longitudinal analysis of the Swedish National Diabetes Register | 2011 | Risk not calculated |
| A. M. Ali, M. D. Loeffler, P. Aylin, and A. Bottle | Predictors of 30-day readmission after total knee arthroplasty: analysis of 566,323 procedures in the United Kingdom | 2019 | Risk not calculated |
| L. Allet, O. Giet, J. Barral, N. Junod, D. Durrer, F. Amati, G. P. Sykiotis, P. Marques-Vidal, and J. J. Puder | Educational level is related to physical fitness in patients with type 2 diabetes - a cross-sectional study | 2016 | Confounding variables |
| A. Alonso-Fernández, M. de la Peña, D. Romero, J. Piérola, M. Carrera, A. Barceló, J. B. Soriano, A. García Suquia, C. Fernández-Capitán, A. Lorenzo, and F. García-Río | Association between obstructive sleep apnea and pulmonary embolism | 2013 | Confounding variables |
| I. B. Ancheta, A. Volgman, C. V. Ancheta, and C. Battie | Comparison of cardiovascular disease risk scores in Filipino-American women: results from the Filipino Across the Nations Study (FANS) | 2014 | Abstract only |
| D. Andernord, N. Desai, H. Björnsson, M. Ylander, J. Karlsson, and K. Samuelsson | Patient predictors of early revision surgery after anterior cruciate ligament reconstruction: a cohort study of 16,930 patients with 2-year follow-up | 2015 | Risk not calculated |
| S. S. Andersen, C. Andersson, S. M. Berger, T. B. Jensen, C. T. Torp-Pedersen, G. H. Gislason, L. Køber, and M. D. Schmiegelow | Impact of metabolic disorders on the relation between overweight/obesity and incident myocardial infarction and ischaemic stroke in fertile women: a nationwide cohort study | 2015 | Risk not calculated |
| L. J. Andes, Y. J. Cheng, D. B. Rolka, E. W. Gregg, and G. Imperatore | Prevalence of prediabetes among adolescents and young adults in the United States, 2005-2016 | 2020 | Risk not calculated |
| J. Araújo, H. Barros, E. Ramos, and L. Li | Trajectories of total and central adiposity throughout adolescence and cardiometabolic factors in early adulthood | 2016 | Abstract only |
| R. Ariely, R. Klein, W. K. Tham, C. Bell, L. Smolen, and A. Tynan | Identifying and adapting a prognostic risk equation for relevant subpopulations in a pharmacoeconomic model of major adverse cardiovascular event prevention | 2013 | Abstract only |
| J. M. Arteagoitia, J. A. Piniés, J. M. Altzibar, F. Gonzalez-Carril, I. Irigoien, L. Echevarriarteun, and J. L. Rodriguez-Murua | Cardiovascular risk factors outcomes in patients with newly-diagnosed type 2 diabetes. The Basque country 10-yr prospective diabetes study | 2014 | Abstract only |
| L. M. Artigao-Rodenas, J. A. Carbayo-Herencia, A. Palazon-Bru, J. A. Divison-Garrote, C. Sanchis-Domenech, I. Vigo-Aguiar, and V. F. Gil-Guillen | Construction and validation of a 14-year cardiovascular risk score for use in the general population: the Puras-GEVA chart | 2015 | Obesity not defined or not defined by BMI |
| M. Arzt, H. Woehrle, O. Oldenburg, A. Graml, A. Suling, E. Erdmann, H. Teschler, and K. Wegscheider | Prevalence and predictors of sleep-disordered breathing in patients with stable chronic heart failure: the SchlaHF registry | 2016 | Risk not calculated |
| M. Ashworth, S. Durbaba, D. Whitney, J. Crompton, M. Wright, and H. Dodhia | Journey to multimorbidity: longitudinal analysis exploring cardiovascular risk factors and sociodemographic determinants in an urban setting | 2019 | Confounding variables |
| A. T. Atayoglu, N. Inanc, E. Başmisirli, and A. G. Çapar | Evaluation of the Finnish Diabetes Risk Score (FINDRISC) for diabetes screening in Kayseri, Turkey | 2020 | Country |
| J. L. Atkins, P. H. Whincup, R. W. Morris, L. T. Lennon, O. Papacosta, and S. G. Wannamethee | Sarcopenic obesity and risk of cardiovascular disease and mortality: a population-based cohort study of older men | 2014 | Obesity not defined or not defined by BMI |
| R. Aurora, J. Shaw, G. Fulcher, M. Naughton, P. Cistulli, R. M. Bergenstal, P. Zimmet, and N. M. Punjabi | Utility of the Berlin questionnaire in identifying obstructive sleep apnea in patients with type 2 diabetes | 2012 | Abstract only |
| A. Azeem, S. K. Musani, W. L. Beard, T. H. Mosley, K. R. Butler, and E. R. Fox | Long term prediction of incident heart failure in African Americans - the Atherosclerosis Risk in Communities study | 2016 | Abstract only |
| J. Badarienė, E. Rinkūnienė, V. Kasiulevičius, G. Smaliukaitė, V. Selskaitė, J. Barysienė, and V. Dženkevičiūtė | Management of patients with coronary artery disease in Lithuania: a comparison with other central Eastern European countries based on data from the international CLARIFY registry | 2020 | Risk not calculated |
| A. Badawi, G. D. Giuseppe, and P. Arora | Cardiovascular disease risk in patients with hepatitis C infection: results from two general population health surveys in Canada and the United States (2007-2017) | 2018 | Risk equation not reported |
| E. Badrick, M. Sperrin, I. E. Buchan, and A. G. Renehan | Obesity paradox and mortality in adults with and without incident type 2 diabetes: a matched population-level cohort study | 2017 | Risk not calculated |
| S. Bailly, M. Destors, Y. Grillet, P. Richard, B. Stach, I. Vivodtzev, J. F. Timsit, P. Lévy, R. Tamisier, and J. L. Pépin | Obstructive sleep apnea: a cluster analysis at time of diagnosis | 2016 | Risk equation not reported |
| F. Banfi-Bacsardi, Z. Ruzsa, A. Lux, I. Edes, L. Molnar, G. Y. Barczi, D. Becker, and B. Merkely | The specific characteristics and independent predictors of no-reflow phenomenon, development of a clinically-adaptable risk estimation system | 2019 | Abstract only |
| E. Banks, J. Welsh, G. Joshy, M. Martin, E. Paige, and R. J. Korda | Comparison of cardiovascular disease risk factors, assessment and management in men and women, including consideration of absolute risk: a nationally representative cross-sectional study | 2020 | Risk not calculated |
| B. J. M. H. Barbara J.M.H. Jefferis, P. H. Whincup, L. Lennon, and S. G. Wannamethee | Low levels of physical activity protect against onset of type 2 diabetes in older men; a British population based prospective cohort study | 2011 | Abstract only |
| M. Barroso, A. Goday, R. Ramos, A. Marín-Ibañez, M. J. Guembe, F. Rigo, M. J. Tormo-Díaz, C. Moreno-Iribas, J. J. Cabré, A. Segura, J. M. Baena-Díez, A. G. de la Cámara, J. Lapetra, M. Quesada, M. J. Medrano, J. Berjón, G. Frontera, D. Gavrila, A. Barricarte, J. Basora, J. M. García, M. García-Lareo, D. Lora-Pablos, E. Mayoral, M. Grau, and J. Marrugat | Interaction between cardiovascular risk factors and body mass index and 10-year incidence of cardiovascular disease, cancer death, and overall mortality | 2018 | Population |
| H. Bays, K. M. Fox, and S. Grandy | Adiposity, age, and family history as a simplified prediction of future diabetes mellitus from the shield study | 2011 | Abstract only |
| G. Beccuti, D. Grimaldi, E. Tasali, V. Abraham, H. Whitmore, D. A. Ehrmann, E. Van Cauter, and B. Mokhlesi | Absence of sex disparity in the prevalence of obstructive sleep apnea in type 2 diabetes mellitus | 2013 | Abstract only |
| M. Beckowski, L. Polonski, M. Gierlotka, T. Zdrojewski, W. Drygas, J. Karwowski, I. Kowalik, and H. Szwed | The cardiovascular disease risk factor profile in a young woman (under the age of 45) with acute coronary syndrome (ACS) | 2015 | Risk not calculated |
| P. J. Belmont, Jr., G. P. Goodman, B. R. Waterman, J. O. Bader, and A. J. Schoenfeld | Thirty-day postoperative complications and mortality following total knee arthroplasty: incidence and risk factors among a national sample of 15,321 patients | 2014 | Confounding variables |
| T. M. Bergsten, A. Nicholson, R. Donnino, B. Wang, Y. Fang, and S. Natarajan | Predicting adults likely to develop heart failure using readily available clinical information: an analysis of heart failure incidence using the NHEFS | 2020 | Confounding variables |
| G. Bevan, C. De Poli, M. J. Keng, and R. Raine | How valid are projections of the future prevalence of diabetes? Rapid reviews of prevalence-based and Markov chain models and comparisons of different models' projections for England | 2020 | Obesity not defined or not defined by BMI |
| P. T. Bhattacharya, R. R. Golamari, S. Vunnam, S. Moparthi, N. Venkatappa, D. J. Dollard, J. Missri, W. Yang, S. E. Kimmel, and G. Tarantino | Predictive risk stratification using HEART (history, electrocardiogram, age, risk factors, and initial troponin) and TIMI (thrombolysis in myocardial infarction) scores in non-high risk chest pain patients: an African American urban community based hospital study | 2019 | Obesity not defined or not defined by BMI |
| L. Björck, M. Novak, M. Schaufelberger, K. W. Giang, and A. Rosengren | Body weight in midlife and long-term risk of developing heart failure-a 35-year follow-up of the primary prevention study in Gothenburg, Sweden | 2015 | Obesity not defined or not defined by BMI |
| F. Bocchi, P. Marques-Vidal, E. Pruvot, G. Waeber, P. Vollenweider, and D. Gachoud | Clinical and biological determinants of P-wave duration: cross-sectional data from the population-based CoLaus\|PsyCoLaus study | 2020 | Confounding variables |
| V. Boddapati, M. C. Fu, B. U. Nwachukwu, C. L. Camp, A. M. Spiker, R. J. Williams, and A. S. Ranawat | Procedure length is independently associated with overnight hospital stay and 30-day readmission following anterior cruciate ligament reconstruction | 2020 | Risk not calculated |
| J. Bodin, C. Ha, C. Sérazin, A. Descatha, A. Leclerc, M. Goldberg, and Y. Roquelaure | Effects of individual and work-related factors on incidence of shoulder pain in a large working population | 2012 | Confounding variables |
| D. D. Bohl, M. G. Maltenfort, R. Huang, J. Parvizi, J. R. Lieberman, and C. J. Della Valle | Development and validation of a risk stratification system for pulmonary embolism after elective primary total joint arthroplasty | 2016 | Risk not calculated |
| J. Boivin, A. Quartino, R. Fay, N. Girerd, and P. Rossignol | Clinical and biological associated factors with elevated ankle brachial index in general practice | 2015 | Abstract only |
| M. Bonaccio, A. Di Castelnuovo, S. Costanzo, A. De Curtis, M. Persichillo, C. Cerletti, M. B. Donati, G. de Gaetano, and L. Iacoviello | Impact of combined healthy lifestyle factors on survival in an adult general population and in high-risk groups: prospective results from the Moli-sani Study | 2019 | Obesity not defined or not defined by BMI |
| I. Bouloukaki, F. Kapsimalis, C. Mermigkis, M. Kryger, N. Tzanakis, P. Panagou, V. Moniaki, E. M. Vlachaki, G. Varouchakis, N. M. Siafakas, and S. E. Schiza | Prediction of obstructive sleep apnea syndrome in a large Greek population | 2010 | Published pre-2011 |
| D. W. Bowden, F. C. Hsu, B. I. Freedman, J. Carr, and A. J. Cox | Contributors to mortality in high risk diabetes patients | 2014 | Abstract only |
| J. Brandts, S. R. Tittel, P. Bramlage, T. Danne, P. Schermann, E. Hess, M. Huptas, R. W. Holl, and D. Müller-Wieland | Remnant cholesterol in individuals with type 2 diabetes: correlation to components of the metabolic syndrome and triglyceride-glucose index in the DIVE and DPV registries | 2020 | Abstract only |
| J. Bressler, J. S. Pankow, J. Coresh, and E. Boerwinkle | Interaction between the NOS3 gene and obesity as a determinant of risk of type 2 diabetes: the Atherosclerosis Risk in Communities study | 2013 | Confounding variables |
| E. Bruckert, S. Kownator, J. Dallongeville, G. Bonnelye, F. Thomas-Delecourt, P. H. Delaage, and J. Ferrières | Cardiovascular risk prevalence in high risk primary care patients not treated with lipid-lowering treatment in France, results of an online study | 2013 | Abstract only |
| E. M. Bucholz, K. M. Strait, R. P. Dreyer, S. T. Lindau, G. D'Onofrio, M. Geda, E. S. Spatz, J. F. Beltrame, J. H. Lichtman, N. P. Lorenze, H. Bueno, and H. M. Krumholz | Editor's Choice-Sex differences in young patients with acute myocardial infarction: a VIRGO study analysis | 2017 | Risk not calculated |
| M. Budnik and G. Opolski | The assessment of coronary heart disease risk factors correlated with demographic and social data in post-coronary intervention patients in Polish population | 2015 | Risk not calculated |
| N. Burokienė, I. Domarkienė, L. Ambrozaitytė, I. Uktverytė, R. Meškienė, D. Karčiauskaitė, V. Kasiulevičius, V. Šapoka, V. Kučinskas, and Z. A. Kučinskienė | Classical rather than genetic risk factors account for high cardiovascular disease prevalence in Lithuania: a cross-sectional population study | 2017 | Confounding variables |
| D. Canoy, B. J. Cairns, A. Balkwill, F. Lucy Wright, J. Green, G. Reeves, and V. Beral | Coronary heart disease incidence in women by waist circumference within categories of body mass index | 2013 | Risk not calculated |
| L. M. S. Carlsson, K. Sjöholm, P. Jacobson, J. C. Andersson-Assarsson, P. A. Svensson, M. Taube, B. Carlsson, and M. Peltonen | Life expectancy after bariatric surgery in the Swedish Obese Subjects study | 2020 | Confounding variables |
| L. M. S. Carlsson, K. Sjöholm, C. Karlsson, P. Jacobson, J. C. Andersson-Assarsson, P. A. Svensson, I. Larsson, S. Hjorth, M. Neovius, M. Taube, B. Carlsson, and M. Peltonen | Long-term incidence of microvascular disease after bariatric surgery or usual care in patients with obesity, stratified by baseline glycaemic status: a post-hoc analysis of participants from the Swedish Obese Subjects study | 2017 | Confounding variables |
| J. M. Caswell | Exploring spatial trends in Canadian incidence of hospitalization due to myocardial infarction with additional determinants of health | 2016 | Risk not calculated |
| F. L. Cavalot, A. Pagliarino, M. Valle, L. Di Martino, F. Mazzaglia, K. Bonomo, and M. Trovati | Gender differences in cardiovascular events, cardiovascular and all-cause mortality in type 2 diabetes: lesson from the San Luigi Gonzaga Diabetes study | 2013 | Abstract only |
| M. Charakida, T. Khan, W. Johnson, N. Finer, J. Woodside, P. H. Whincup, N. Sattar, D. Kuh, R. Hardy, and J. Deanfield | Lifelong patterns of BMI and cardiovascular phenotype in individuals aged 60-64 years in the 1946 British birth cohort study: an epidemiological study | 2014 | Population |
| K. Chau, N. Girerd, E. Bozec, J. P. Ferreira, K. Duarte, J. A. Nazare, M. Laville, A. Benetos, F. Zannad, J. M. Boivin, and P. Rossignol | Association between abdominal adiposity and 20-year subsequent aortic stiffness in an initially healthy population-based cohort | 2018 | Confounding variables |
| Y. Y. Commodore-Mensah, C. A. Berko, M. E. Sampah, J. Cudjoe, and C. D. Himmelfarb | The “Afro-Cardiac study” identifies high burden of cardiovascular disease risk in West African immigrants in the United States | 2015 | Abstract only |
| P. Costanzo, D. Hepburn, T. Sathyapalan, J. Cleland, A. Clark, E. Kilpatrick, and S. Atkin | Impact of the body mass index on the cause of mortality in type 2 diabetes from the “obesity paradox” cohort | 2017 | Abstract only |
| G. Danaei, M. A. Hernán, and F. B. Hu | Using parametric G-formula to estimate the effect of multiple lifestyle and dietary interventions for preventing type 2 diabetes in a prospective cohort | 2011 | Abstract only |
| A. Deev, A. Kapustina, Y. Balanova, A. Imaeva, S. Shalnova, and V. Shkolnikov | Prediction of all-cause and cardiovascular disease mortality using the counting scale of selected diseases and risk factors among Muscovites of advanced ages | 2017 | Abstract only |
| S. S. Dhaliwal, T. A. Welborn, and P. A. Howat | Recreational physical activity as an independent predictor of multivariable cardiovascular disease risk | 2013 | Obesity not defined or not defined by BMI |
| C. Di Tecco, L. Fontana, G. Adamo, M. Petyx, and S. Iavicoli | Gender differences and occupational factors for the risk of obesity in the Italian working population | 2020 | Confounding variables |
| W. Doehner, E. Erdmann, R. Cairns, A. L. Clark, J. A. Dormandy, E. Ferrannini, and S. D. Anker | Inverse relation of body weight and weight change with mortality and morbidity in patients with type 2 diabetes and cardiovascular co-morbidity: an analysis of the PROactive study population | 2012 | Risk not calculated |
| N. Doody, C. McIntyre, and A. Morrison | A comparison of two screening tools for obstructive sleep apnoea in the surgical pre-assessment clinic | 2012 | Abstract only |
| K. Dragsbæk, J. S. Neergaard, M. Karsdal, and C. Christiansen | Metabolic syndrome in elderly women: is waist circumference the correct 'entrance criteria'? | 2015 | Abstract only |
| K. R. Duchman, A. J. Pugely, C. T. Martin, Y. Gao, N. A. Bedard, and J. J. Callaghan | Operative time affects short-term complications in total joint arthroplasty | 2017 | Confounding variables |
| M. K. Edwards, O. Addoh, and P. D. Loprinzi | Predictive validity of the ACC/AHA pooled cohort equations in predicting residual-specific mortality in a national prospective cohort study of adults in the United States | 2016 | Confounding variables |
| K. Eeg-Olofsson, S. Gudbjörnsdottir, B. Eliasson, B. Zethelius, and J. Cederholm | The triglycerides-to-HDL-cholesterol ratio and cardiovascular disease risk in obese patients with type 2 diabetes: a report from the Swedish National Diabetes Register | 2013 | Duplicate |
| T. W. Elffers, R. De Mutsert, H. J. Lamb, A. C. Maan, P. W. Macfarlane, J. A. P. Willems Van Dijk, F. R. Rosendaal, J. W. Jukema, and S. Trompet | The associations of overall and abdominal adiposity with ECG measures of subclinical cardiovascular disease in a middle-aged population | 2017 | Abstract only |
| B. Eliasson, V. Liakopoulos, S. Franzén, I. Näslund, A. M. Svensson, J. Ottosson, and S. Gudbjörnsdottir | Cardiovascular disease and mortality in patients with type 2 diabetes after bariatric surgery in Sweden: a nationwide, matched, observational cohort study | 2015 | Confounding variables |
| J. Engel, I. van der Wulp, M. de Bruijne, and C. Wagner | A cross-sectional multicentre study of cardiac risk score use in the management of unstable angina and non-ST-elevation myocardial infarction | 2015 | Population |
| B. S. Ferket, B. J. H. van Kempen, J. Heeringa, S. Spronk, K. E. Fleischmann, R. L. G. Nijhuis, A. Hofman, E. W. Steyerberg, and M. G. M. Hunink | Personalized prediction of lifetime benefits with statin therapy for asymptomatic individuals: a modeling study | 2012 | Confounding variables |
| I. Figliuzzi, V. Presta, B. Citoni, F. Miceli, F. Simonelli, A. Battistoni, R. Coluccia, A. Ferrucci, M. Volpe, and G. Tocci | Achievement of multiple therapeutic targets for cardiovascular disease prevention: retrospective analysis of real practice in Italy | 2018 | Duplicate |
| I. Florath, S. Brandt, M. N. Weck, A. Moss, P. Gottmann, D. Rothenbacher, M. Wabitsch, and H. Brenner | Evidence of inappropriate cardiovascular risk assessment in middle-age women based on recommended cut-points for waist circumference | 2014 | Confounding variables |
| B. T. Fokkens, R. P. Van Waateringe, D. J. Mulder, B. H. R. Wolffenbuttel, and A. J. Smit | Skin autofluorescence improves the Finnish Diabetes Risk Score in the detection of diabetes in a large population cohort | 2017 | Abstract only |
| V. Foos, M. Lamotte, and P. McEwan | Contrasting the relative risk reduction of cardiovascular events in the core diabetes model associated with single risk factor changes across alternative risk engines: UKPDS68, UKPDS82 and Swedish National Diabetes Registry equations | 2015 | Abstract only |
| R. Fukuoka, T. Kohno, S. Kohsaka, Y. Shiraishi, M. Sawano, T. Abe, Y. Nagatomo, A. Goda, A. Mizuno, K. Fukuda, R. Shadman, T. F. Dardas, W. C. Levy, and T. Yoshikawa | Predicting sudden cardiac death in Japanese heart failure patients: international validation of the Seattle Proportional Risk Model | 2018 | Abstract only |
| R. Gascuena, L. Gomez Paredes, J. Munoz Gutierrez, J. M. Mendiguren, L. Calderon, M. Cogollos, and C. Lledo | Improvement in cardiovascular risk scores by a diet and exercise programme for obese and overweighted young adults | 2017 | Abstract only |
| R. Germany, J. Kalgreen, A. Ketelsen, and L. Austin | What is the likelihood of sleep disordered breathing in your heart failure patient? | 2011 | Abstract only |
| N. Geubbels, M. De Brauw, Y. I. Acherman, A. W. Van De Laar, and S. C. Bruin | How risk stratification models fail to predict adverse outcomes in a large Dutch bariatric cohort | 2014 | Abstract only |
| S. Gibson and M. Ashwell | A simple cut-off for waist-to-height ratio (0·5) can act as an indicator for cardiometabolic risk: recent data from adults in the Health Survey for England | 2020 | Obesity not defined or not defined by BMI |
| S. N. Gobardhan, A. C. Dimitriu-Leen, A. R. van Rosendael, E. W. van Zwet, C. J. Roos, P. V. Oemrawsingh, A. V. Kharagjitsingh, J. W. Jukema, V. Delgado, M. J. Schalij, J. J. Bax, and A. J. H. A. Scholte | Prevalence by computed tomographic angiography of coronary plaques in South Asian and white patients with type 2 diabetes mellitus at low and high risk using four cardiovascular risk scores (UKPDS, FRS, ASCVD, and JBS3) | 2017 | Country |
| J. G. Godino, E. M. Van Sluijs, S. S. Sutton, and S. J. Griffin | Understanding perceived risk of type 2 diabetes in healthy middle-aged adults | 2014 | Obesity not defined or not defined by BMI |
| D. M. Grant, P. McEwan, V. Foos, J. Palmer, and A. Lloyd | Are we failing to capture the relationship between body mass index and cardiovascular disease and mortality in subjects with type 2 diabetes? | 2013 | Abstract only |
| P. Graversen, S. Z. Abildstrøm, L. Jespersen, A. Borglykke, and E. Prescott | Cardiovascular risk prediction: Can Systematic Coronary Risk Evaluation (SCORE) be improved by adding simple risk markers? Results from the Copenhagen City Heart Study | 2016 | Confounding variables |
| L. J. Gray, M. Davies, D. R. Webb, N. A. Taub, and K. Khunti | The Leicester Practice Computer Risk Score - An automated tool for identifying those with impaired glucose regulation or type 2 diabetes mellitus using the new diagnostic criteria | 2011 | Abstract only |
| E. W. Gregg, J. M. Jakicic, G. Blackburn, P. Bloomquist, G. A. Bray, J. M. Clark, M. Coday, J. M. Curtis, C. Egan, M. Evans, J. Foreyt, G. Foster, H. P. Hazuda, J. O. Hill, E. S. Horton, V. S. Hubbard, R. W. Jeffery, K. C. Johnson, A. E. Kitabchi, W. C. Knowler, A. Kriska, W. Lang, C. E. Lewis, M. G. Montez, D. M. Nathan, R. H. Neiberg, J. Patricio, A. Peters, X. Pi-Sunyer, H. Pownall, B. Redmon, J. Regensteiner, J. Rejeski, P. M. Ribisl, M. Safford, K. Stewart, D. Trence, T. A. Wadden, R. R. Wing, and S. Z. Yanovski | Association of the magnitude of weight loss and changes in physical fitness with long-term cardiovascular disease outcomes in overweight or obese people with type 2 diabetes: a post-hoc analysis of the Look AHEAD randomised clinical trial | 2016 | Obesity not defined or not defined by BMI |
| M. Guasch-Ferré, M. Bulló, B. Costa, M. Martínez-Gonzalez, N. Ibarrola-Jurado, R. Estruch, F. Barrio, and J. Salas-Salvadó | A risk score to predict type 2 diabetes mellitus in an elderly Spanish Mediterranean population at high cardiovascular risk | 2012 | Duplicate |
| M. Guasch-Ferré, M. Bulló, M. Á. Martínez-González, D. Corella, R. Estruch, M. I. Covas, F. Arós, J. Wärnberg, M. Fiol, J. Lapetra, M. Á. Muñoz, L. Serra-Majem, X. Pintó, N. Babio, A. Díaz-López, and J. Salas-Salvadó | Waist-to-height ratio and cardiovascular risk factors in elderly individuals at high cardiovascular risk | 2012 | Confounding variables |
| R. Guijarro, J. Montes, C. S. Roman, J. I. Arcelus, G. Barillari, X. Granero, and M. Monreal | Venous thromboembolism and bleeding after total knee and hip arthroplasty: findings from the Spanish National Discharge Database | 2011 | Confounding variables |
| M. Gupta, A. Dugan, E. Chacon, D. L. Davenport, M. B. Shah, F. Marti, J. S. Roth, A. Bernard, J. B. Zwischenberger, and R. Gedaly | Detailed perioperative risk among patients with extreme obesity undergoing nonbariatric general surgery | 2020 | Confounding variables |
| E. Hagman, P. Danielsson, L. Brandt, A. Ekbom, and C. Marcus | Association between impaired fasting glycaemia in pediatric obesity and type 2 diabetes in young adulthood | 2016 | Population |
| J. P. J. Halcox, F. Tubach, J. R. Banegas, C. Borghi, J. Dallongeville, G. De Backer, E. Guallar, J. Perk, P. G. Steg, and F. Rodriguez-Artalejo | Reclassification of cardiovascular risk in Europe: application of the updated Systematic COronary Risk Evaluation (SCORE) algorithm incorporating high-density lipoprotein levels | 2012 | Abstract only |
| A. K. Halldin, L. Lissner, B. Lernfelt, and C. Björkelund | Impact of changes in physical activity or BMI on risk of heart failure in women - the prospective population study of women in Gothenburg | 2020 | Confounding variables |
| A. K. Halldin, M. Schaufelberger, B. Lernfelt, L. Björck, A. Rosengren, L. Lissner, and C. Björkelund | Obesity in middle age increases risk of later heart failure in women—results from the prospective Population Study of Women and H70 studies in Gothenburg, Sweden | 2017 | Confounding variables |
| M. Hamer, G. D. Batty, E. Stamatakis, and M. Kivimaki | Comparison of risk factors for fatal stroke and ischemic heart disease: a prospective follow up of the health survey for England | 2011 | Obesity not defined or not defined by BMI |
| L. Han, D. You, W. Ma, T. Astell-Burt, X. Feng, S. Duan, and L. Qi | National trends in American Heart Association revised Life's Simple 7 metrics associated with risk of mortality among US adults | 2019 | Confounding variables |
| R. J. Hanusek, B. A. Grice, J. Pomeroy, W. C. Knowler, and R. L. Hanson | Comparison of regression and ratio indices for weight or waist circumference adjusted for height as predictors of diabetes | 2014 | Abstract only |
| J. Harrington, A. P. Fitzgerald, P. M. Kearney, D. L. Dahly, V. J. C. McCarthy, E. Kennedy, and I. J. Perry | Diet quality, insulin resistance and risk of type 2 diabetes in middle-aged men and women: results from the Cork and Kerry Diabetes and Heart Disease study | 2012 | Abstract only |
| E. W. Holroyd, A. Sirker, C. S. Kwok, E. Kontopantelis, P. F. Ludman, M. A. De Belder, R. Butler, J. Cotton, A. Zaman, and M. A. Mamas | The relationship of body mass index to percutaneous coronary intervention outcomes: does the obesity paradox exist in contemporary percutaneous coronary intervention cohorts? Insights from the British Cardiovascular Intervention Society Registry | 2017 | Confounding variables |
| Q. Huynh, E. K. Chowdhury, M. M. Huq, B. Billah, C. M. Reid, and M. R. Nelson | Prediction of cardiovascular and all-cause mortality at 10 years in the hypertensive aged | 2014 | Duplicate |
| Q. L. Huynh, C. M. Reid, E. K. Chowdhury, M. M. Huq, B. Billah, L. M. Wing, A. M. Tonkin, L. A. Simons, and M. R. Nelson | Prediction of cardiovascular and all-cause mortality at 10 years in the hypertensive aged population | 2015 | Risk not calculated |
| A. Jagodzinski, F. Ojeda, T. Zeller, K. Sydow, S. Blankenberg, T. Jorgensen, K. Kuulasma, R. B. Schnabel, and V. Salomaa | Development of a European heart failure risk score in the general population | 2013 | Abstract only |
| B. J. Jefferis, L. Lennon, P. H. Whincup, and S. G. Wannamethee | Longitudinal associations between changes in physical activity and onset of type 2 diabetes in older British men: the influence of adiposity | 2012 | Population |
| D. A. Jenkins, J. Bowden, H. A. Robinson, N. Sattar, R. J. F. Loos, M. K. Rutter, and M. Sperrin | Adiposity-mortality relationships in type 2 diabetes, coronary heart disease, and cancer subgroups in the UK Biobank, and their modification by smoking | 2018 | Risk not calculated |
| D. J. Johnson, J. P. Castle, M. J. Hartwell, A. M. D'Heurle, and D. W. Manning | Risk factors for greater than 24-hour length of stay after primary total knee arthroplasty | 2020 | Risk not calculated |
| C. A. Jones, V. Cox, G. S. Jhangri, and M. E. Suarez-Almazor | Delineating the impact of obesity and its relationship on recovery after total joint arthroplasties | 2012 | Risk not calculated |
| G. Joodi, J. A. Maradey, B. Bogle, M. Mirzaei, M. I. Sadaf, I. Pursell, C. Henderson, J. P. Mounsey, and R. J. Simpson | Coronary artery disease and atherosclerotic risk factors in a population-based study of sudden death | 2020 | Risk not calculated |
| M. D. Kashani, A. H. Eliasson, K. Bailey, and M. N. Vernalis | Novel tool improves cardiovascular risk stratification and guides therapy | 2011 | Abstract only |
| M. A. Khan, R. Grinberg, S. Johnson, J. N. Afthinos, and K. E. Gibbs | Perioperative risk factors for 30-day mortality after bariatric surgery: is functional status important? | 2013 | Risk not calculated |
| N. Kleefstra, G. W. D. Landman, K. J. J. Van Hateren, R. O. B. Gans, K. H. Groenier, and H. J. G. Bilo | Calculating risk in patients with type 2 diabetes in the Netherlands: UKPDS versus ZODIAC risk engine performance (ZODIAC-26) | 2011 | Abstract only |
| B. Klijs, S. J. Otto, R. J. Heine, Y. van der Graaf, J. J. Lous, and H. J. de Koning | Screening for type 2 diabetes in a high-risk population: study design and feasibility of a population-based randomized controlled trial | 2012 | Obesity not defined or not defined by BMI |
| R. J. Korda, B. Liu, M. S. Clements, A. E. Bauman, L. R. Jorm, H. J. Bambrick, and E. Banks | Prospective cohort study of body mass index and the risk of hospitalisation: findings from 246361 participants in the 45 and Up Study | 2013 | Risk equation not reported |
| B. Kowall, W. Rathmann, M. Heier, R. Holle, A. Peters, B. Thorand, C. Herder, K. Strassburger, G. Giani, and C. Meisinger | Impact of weight and weight change on normalization of prediabetes and on persistence of normal glucose tolerance in an older population: the KORA S4/F4 study | 2012 | Risk equation not reported |
| B. Kowall, W. Rathmann, O. Kuss, C. Herder, M. Roden, A. Stang, C. Huth, B. Thorand, C. Meisinger, and A. Peters | Reversion from prediabetes to normoglycaemia after weight change in older persons: the KORA F4/FF4 study | 2021 | Risk equation not reported |
| B. Kowall, A. Stang, R. Erbel, S. Moebus, A. Petersmann, A. Steveling, K. H. Jöckel, and H. Völzke | Is the obesity paradox in type 2 diabetes due to artefacts of biases? An analysis of pooled cohort data from the Heinz Nixdorf Recall study and the Study of Health in Pomerania | 2020 | Risk not calculated |
| M. J. Kwasny, A. I. Edelstein, and D. W. Manning | Statistical methods dictate the estimated impact of body mass index on major and minor complications after total joint arthroplasty | 2018 | Confounding variables |
| M. Lamotte, V. Foos, and P. McEwan | Contrasting eight cardiovascular risk equations for use in type 2 diabetes cohorts using the CORE Diabetes Model | 2015 | Abstract only |
| J. R. Lewis, S. S. Dhaliwal, K. Zhu, and R. L. Prince | A predictive model for knee joint replacement in older women | 2013 | Risk equation not reported |
| M. Li, M. L. Rahman, J. Wu, M. Ding, J. E. Chavarro, Y. Lin, S. H. Ley, L. Grunnet, S. Hinkle, A. C. B. Thuesen, E. Yeung, R. E. Gorelangton, S. J. Sherman, L. Hjort, F. B. Kampmann, P. Damm, F. Tekola-Ayele, A. Liu, J. Mills, A. A. Vaag, S. F. O. Esq, F. Hu, and C. Zhang | Genetic risk score of type 2 diabetes and progression risk from gestational diabetes to type 2 diabetes: results from two independent populations | 2019 | Abstract only |
| V. Liakopoulos, S. Franzén, A. M. Svensson, M. Miftaraj, J. Ottosson, I. Näslund, S. Gudbjörnsdottir, and B. Eliasson | Pros and cons of gastric bypass surgery in individuals with obesity and type 2 diabetes: nationwide, matched, observational cohort study | 2019 | Risk not calculated |
| V. Liakopoulos, S. Franzén, A. M. Svensson, B. Zethelius, J. Ottosson, I. Näslund, S. Gudbjörnsdottir, and B. Eliasson | Changes in risk factors and their contribution to reduction of mortality risk following gastric bypass surgery among obese individuals with type 2 diabetes: a nationwide, matched, observational cohort study | 2017 | Confounding variables |
| A. López-De-Andrés, V. Hernández-Barrera, M. A. Martínez-Huedo, M. Villanueva-Martinez, I. Jiménez-Trujillo, and R. Jiménez-García | Type 2 diabetes and in-hospital complications after revision of total hip and knee arthroplasty | 2017 | Risk not calculated |
| L. Lorgis, Y. Cottin, N. Danchin, L. Mock, P. Sicard, P. Buffet, I. L'Huillier, C. Richard, J. C. Beer, C. Touzery, P. Gambert, and M. Zeller | Impact of obesity on the prognostic value of the N-terminal pro-B-type natriuretic peptide (NT-proBNP) in patients with acute myocardial infarction | 2011 | Confounding variables |
| D. M. Lyall, C. Celis-Morales, J. Ward, S. Iliodromiti, J. J. Anderson, J. M. R. Gill, D. J. Smith, U. E. Ntuk, D. F. MacKay, M. V. Holmes, N. Sattar, and J. P. Pell | Body-mass index and cardiometabolic disease: a Mendelian randomisation study of UK Biobank participants | 2016 | Abstract only |
| D. M. Lyall, C. Celis-Morales, J. Ward, S. Iliodromiti, J. J. Anderson, J. M. R. Gill, D. J. Smith, U. E. Ntuk, D. F. Mackay, M. V. Holmes, N. Sattar, and J. P. Pell | Association of body mass index with cardiometabolic disease in the UK Biobank: a mendelian randomization study | 2017 | Confounding variables |
| A. G. Mainous, R. J. Tanner, K. P. Rahmanian, A. Jo, and P. J. Carek | Effect of sedentary lifestyle on cardiovascular disease risk among healthy adults with body mass indexes 18.5 to 29.9 kg/m^2^ | 2019 | Population |
| M. Martin O'flaherty, J. Critchley, S. Wild, N. Unwin, and S. Capewell | Forecasting U.S. type 2 diabetes prevalence to 2030: validation of a simple model | 2011 | Abstract only |
| M. A. Martínez-Huedo, R. Jiménez-García, I. Jiménez-Trujillo, V. Hernández-Barrera, B. del Rio Lopez, and A. López-de-Andrés | Effect of type 2 diabetes on in-hospital postoperative complications and mortality after primary total hip and knee arthroplasty | 2017 | Obesity not defined or not defined by BMI |
| A. Mavrea, O. Ancusa, I. Citu, and M. Tomescu | Obesity and metabolic syndrome in heart failure with preserved ejection fraction | 2013 | Abstract only |
| A. Meadows, S. Wright, and M. Lillicrap | Patients with psoriatic arthritis are at high risk of myocardial infarction/cardiovascular disease according to QRISK2 | 2017 | Abstract only |
| H. Mohammadi, J. Ohm, A. Discacciati, J. Sundstrom, K. Hambraeus, T. Jernberg, and P. Svensson | Abdominal obesity and the risk of recurrent atherosclerotic cardiovascular disease after myocardial infarction | 2020 | Population |
| M. L. Mongraw-Chaffin, C. A. M. Anderson, J. M. Clark, and W. L. Bennett | Prepregnancy body mass index and cardiovascular disease mortality: the Child Health and Development Studies | 2014 | Confounding variables |
| M. L. Mongraw-Chaffin, S. A. E. Peters, R. R. Huxley, and M. Woodward | The sex-specific association between BMI and coronary heart disease: a systematic review and meta-analysis of 95 cohorts with 1·2 million participants | 2015 | Population |
| I. J. Neeland, A. T. Turer, C. R. Ayers, T. M. Powell-Wiley, G. L. Vega, R. Farzaneh-Far, S. M. Grundy, A. Khera, D. K. McGuire, and J. A. De Lemos | Dysfunctional adiposity and the risk of prediabetes and type 2 diabetes in obese adults | 2012 | Obesity not defined or not defined by BMI |
| M. A. Ohlsson, L. M. A. Kennedy, T. Juhlin, and O. Melander | Midlife risk factor exposure and incidence of cardiac arrest depending on cardiac or non-cardiac origin | 2017 | Population |
| K. Olson, F. Ahmad, B. Bogle, H. Ning, J. Goldberger, and D. Lloyd-Jones | Sudden cardiac death risk distribution in the United States population: results from the National Health and Nutrition Examination Survey (NHANES), 2005-2012 | 2017 | Risk not calculated |
| K. A. Olson, R. B. Patel, F. S. Ahmad, H. Ning, B. M. Bogle, J. J. Goldberger, and D. M. Lloyd-Jones | Sudden cardiac death risk distribution in the United States population (from NHANES, 2005 to 2012) | 2019 | Duplicate |
| R. S. Padwal, S. W. Klarenbach, X. Wang, A. M. Sharma, S. Karmali, D. W. Birch, and S. R. Majumdar | A simple prediction rule for all-cause mortality in a cohort eligible for bariatric surgery | 2013 | Risk not calculated |
| J. Parissis, D. Farmakis, N. Kadoglou, I. Ikonomidis, E. Fountoulaki, E. Hatziagelaki, S. Deftereos, F. Follath, A. Mebazaa, J. Lekakis, and G. Filippatos | Body mass index in acute heart failure: association with clinical profile, therapeutic management and in-hospital outcome | 2016 | Confounding variables |
| F. Perego, E. Renesto, M. Arquati, L. Scandiani, C. Cogliati, D. Torzillo, L. Zocchi, G. Casazza, P. Duca, S. Chirchiglia, G. Lacaita, M. Panteghini, and M. Cortellaro | Target organ damage in a population at intermediate cardiovascular risk, with adjunctive major risk factors: CArdiovascular PREvention Sacco Study (CAPRESS) | 2011 | Risk not calculated |
| M. Piché, J. Martin, K. Cianflone, M. Bastien, S. Marceau, S. Biron, F. S. Hould, and P. Poirier | Changes in predicted cardiovascular disease risk after biliopancreatic diversion surgery in severely obese patients | 2014 | Confounding variables |
| M. Plecka Östlund, R. Marsk, F. Rasmussen, J. Lagergren, and E. Näslund | Morbidity and mortality before and after bariatric surgery for morbid obesity compared with the general population | 2011 | Risk not calculated |
| I. Ponce-Garcia, M. Simarro-Rueda, J. A. Carbayo-Herencia, J. A. Divisón-Garrote, L. M. Artigao-Ródenas, F. Botella-Romero, A. Palazón-Bru, D. R. Martínez-St John, and V. F. Gil-Guillén | Prognostic value of obesity on both overall mortality and cardiovascular disease in the general population | 2015 | Confounding variables |
| A. E. Pontiroli, A. S. Zakaria, M. Fanchini, C. Osio, E. Tagliabue, G. Micheletto, A. Saibene, and F. Folli | A 23-year study of mortality and development of co-morbidities in patients with obesity undergoing bariatric surgery (laparoscopic gastric banding) in comparison with medical treatment of obesity | 2018 | Risk not calculated |
| A. E. Pontiroli, A. S. Zakaria, E. Mantegazza, A. Morabito, A. Saibene, E. Mozzi, and G. Micheletto | Long-term mortality and incidence of cardiovascular diseases and type 2 diabetes in diabetic and nondiabetic obese patients undergoing gastric banding: a controlled study | 2016 | Confounding variables |
| K. Rahimi, D. Bennett, N. Conrad, T. M. Williams, J. Basu, J. Dwight, M. Woodward, A. Patel, J. McMurray, and S. MacMahon | Risk prediction in patients with heart failure: a systematic review and analysis | 2014 | Risk equation not reported |
| J. Robertson, M. Lindgren, M. Schaufelberger, M. Adiels, L. Björck, C. E. Lundberg, N. Sattar, A. Rosengren, and M. Åberg | Body mass index in young women and risk of cardiomyopathy: a long-term follow-up study in Sweden | 2020 | Population |
| K. R. Sahakyan, V. K. Somers, J. P. Rodriguez-Escudero, D. O. Hodge, R. E. Carter, O. Sochor, T. Coutinho, M. D. Jensen, V. L. Roger, P. Singh, and F. Lopez-Jimenez | Normal-weight central obesity: implications for total and cardiovascular mortality | 2015 | Confounding variables |
| B. Sahle, A. J. Owen, and C. M. Reid | Predicting incident heart failure in elderly hypertensive patients: the ANBP2 study | 2016 | Abstract only |
| B. W. Sahle, A. J. Owen, L. M. Wing, M. R. Nelson, G. L. Jennings, and C. M. Reid | Prediction of 10-year risk of incident heart failure in elderly hypertensive population: the ANBP2 study | 2017 | Confounding variables |
| M. Schmidt, H. E. Bøtker, L. Pedersen, and H. T. Sørensen | Young adulthood obesity and risk of acute coronary syndromes, stable angina pectoris, and congestive heart failure: a 36-year cohort study | 2014 | Population |
| M. Schmidt, A. J. Sigrun, S. Lemeshow, T. Lash, P. U. Sinna, H. Erik Boetker, and H. T. Soerensen | Obesity in young adult men and risk of type II diabetes, cardiovascular morbidity, or death before 55 years of age: a Danish 33-year follow-up study | 2013 | Abstract only |
| R. Shadman, J. E. Poole, D. Mozaffarian, J. G. Cleland, K. Swedberg, A. P. Maggioni, I. S. Anand, P. Carson, and W. Levy | Predicting the proportional risk of sudden cardiac death in a multicenter heart failure cohort | 2011 | Abstract only |
| B. Sharif, C. Smith, and D. Marshall | Development of predictive models for medical adverse outcomes during and after primary total joint replacement surgery using obesity and comorbidity indicators | 2017 | Abstract only |
| M. V. Shestakova, A. S. Kolbin, G. R. Galstyan, M. A. Arepeva, E. L. Zaitseva, A. Y. Mayorov, O. I. Karpov, M. A. Proskurin, A. A. Kurilev, and I. I. Dedov | «DIARISK» — The first national prediabetes and diabetes mellitus type 2 risk calculator | 2021 | Obesity not defined or not defined by BMI |
| X. Song, S. Söderberg, P. Jousilahti, C. D. A. Stehouwer, J. S. Yudkin, A. Onat, T. Laatikainen, R. Dankner, R. Morris, J. Tuomilehto, and Q. Qiao | Comparison of abdominal obesity indicators and body mass index as a predictor of mortality among Europeans | 2013 | Abstract only |
| A. Stokes and S. H. Preston | Smoking and reverse causation create an obesity paradox in cardiovascular disease | 2015 | Risk not calculated |
| Y. Sun, B. Liu, L. G. Snetselaar, R. B. Wallace, B. J. Caan, T. E. Rohan, M. L. Neuhouser, A. H. Shadyab, R. T. Chlebowski, J. E. Manson, and W. Bao | Association of normal-weight central obesity with all-cause and cause-specific mortality among postmenopausal women | 2019 | Confounding variables |
| Y. Q. Sun, S. Burgess, J. R. Staley, A. M. Wood, S. Bell, S. K. Kaptoge, Q. Guo, T. R. Bolton, A. M. Mason, A. S. Butterworth, E. Di Angelantonio, G. Å. Vie, J. H. Bjørngaard, J. M. Kinge, Y. Chen, and X. M. Mai | Body mass index and all cause mortality in HUNT and UK Biobank studies: linear and non-linear mendelian randomisation analyses | 2019 | Risk not calculated |
| F. Tabassum and G. D. Batty | Are current UK National Institute for Health and Clinical Excellence (NICE) obesity risk guidelines useful? Cross-sectional associations with cardiovascular disease risk factors in a large, representative English population | 2013 | Risk equation not reported |
| M. Taghavi Azar Sharabiani, T. P. Debray, E. Riboli, M. R. Jarvelin, and P. Vineis | Excessive body weight can predict mortality from ischemic heart disease equally well as cholesterol: evidence from the prospective European EPIC study | 2014 | Abstract only |
| C. Thomas, P. Watson, H. Squires, J. Chilcott, and A. Brennan | Validation of the SPHR diabetes prevention model | 2014 | Abstract only |
| F. Thomas, B. Pannier, A. Benetos, and U. M. Vischer | Visceral obesity is not an independent risk factor of mortality in subjects over 65 years | 2013 | Risk not calculated |
| M. Tohidi, S. B. Brogly, K. Lajkosz, H. J. Grant, E. G. VanDenKerkhof, and A. R. Campbell | Ten-year mortality and revision after total knee arthroplasty in morbidly obese patients | 2018 | Confounding variables |
| G. Veronesi, L. E. Chambless, F. Gianfagna, G. Mancia, G. Cesana, and M. M. Ferrario | Predicting the 20-year risk of first coronary or ischemic stroke event in Northern Italy: the CAMUNI absolute risk equation | 2013 | Abstract only |
| Y. Wang, J. Tuomilehto, P. Jousilahti, R. Antikainen, M. Mähönen, P. T. Katzmarzyk, and G. Hu | Lifestyle factors in relation to heart failure among Finnish men and women | 2011 | Confounding variables |
| C. Weimar, M. Siebler, T. Brandt, D. Römer, L. Rosin, P. Bramlage, and D. Sander | Vascular risk prediction in ischemic stroke patients undergoing in-patient rehabilitation - insights from the investigation of patients with ischemic stroke in neurologic rehabilitation (INSIGHT) registry | 2013 | Obesity not defined or not defined by BMI |
| C. Westerdahl, B. Zöller, E. Arslan, S. Erdine, and P. M. Nilsson | Morbidity and mortality risk among patients with screening-detected severe hypertension in the Malmö Preventive Project | 2014 | Confounding variables |
| J. Winter and A. Wuppermann | Do they know what is at risk? Health risk perception among the obese | 2014 | Risk not calculated |
| M. Yamamoto, G. Mouillet, A. Oguri, M. Gilard, M. Laskar, H. Eltchaninoff, J. Fajadet, B. Iung, P. Donzeau-Gouge, P. Leprince, A. Leuguerrier, A. Prat, M. Lievre, K. Chevreul, J. L. Dubois-Rande, and E. Teiger | Effect of body mass index on 30- and 365-day complication and survival rates of transcatheter aortic valve implantation (from the FRench Aortic National CoreValve and Edwards 2 [FRANCE 2] registry) | 2013 | Confounding variables |
| YanFeng Li, Linda S. Geiss, Nilka R. Burrows, Deborah B. Rolka, and Ann Albright, | Awareness of prediabetes--United States, 2005-2010 | 2013 | Risk equation not reported |
| Global Burden of Metabolic Risk Factors for Chronic Diseases Collaboration | Cardiovascular disease, chronic kidney disease, and diabetes mortality burden of cardiometabolic risk factors from 1980 to 2010: a comparative risk assessment | 2014 | Obesity not defined or not defined by BMI |
| Look AHEAD Research Group | Association of the magnitude of weight loss and changes in physical fitness with long-term cardiovascular disease outcomes in overweight or obese people with type 2 diabetes: a post-hoc analysis of the Look AHEAD randomised clinical trial | 2016 | Risk not calculated |
| Prospective Studies Collaboration and Asia Pacific Cohort Studies Collaboration | Sex-specific relevance of diabetes to occlusive vascular and other mortality: a collaborative meta-analysis of individual data from 980 793 adults from 68 prospective studies | 2018 | Confounding variables |
| L. Albarqouni, J. A. Doust, D. Magliano, E. L. Barr, J. E. Shaw and P. P. Glasziou | External validation and comparison of four cardiovascular risk prediction models with data from the Australian Diabetes, Obesity and Lifestyle study | 2019 | Risk not calculated |
| S. Atasoy, H. Johar, X. Y. Fang, J. Kruse and K. H. Ladwig | Cumulative effect of depressed mood and obesity on type II diabetes incidence: Findings from the MONICA/KORA cohort study | 2018 | Confounding variables |
| M. L. Bayog and C. M. Waters | Cardiometabolic risks, lifestyle health behaviors and heart disease in Filipino Americans | 2017 | Population |
| J. A. Bell, M. Kivimaki and M. Hamer | Metabolically healthy obesity and risk of incident type 2 diabetes: A meta-analysis of prospective cohort studies | 2014 | Confounding variables |
| R. M. Berg, H. Wallaschofski, M. Nauck, R. Rettig, M. R. Markus, R. Laqua, N. Friedrich and A. Hannemann | Positive association between adipose tissue and bone stiffness | 2015 | Risk not calculated |
| M. G. Colombo, C. Meisinger, U. Amann, M. Heier, W. von Scheidt, B. Kuch, A. Peters and I. Kirchberger | Association of obesity and long-term mortality in patients with acute myocardial infarction with and without diabetes mellitus: Results from the MONICA/KORA myocardial infarction registry | 2015 | Risk not calculated |
| D. Culliford, J. Maskell, A. Judge and N. K. Arden | A population-based survival analysis describing the association of body mass index on time to revision for total hip and knee replacements: results from the UK general practice research database | 2013 | Risk not calculated |
| J. Dallongeville, D. L. Bhatt, P. G. Steg, P. Ravaud, P. W. Wilson, K. A. Eagle, S. Goto, J. L. Mas and G. Montalescot | Relation between body mass index, waist circumference, and cardiovascular outcomes in 19,579 diabetic patients with established vascular disease: The REACH Registry | 2012 | Obesity not defined or not defined by BMI |
| P. B. Derman, P. D. Fabricant and G. David | The role of overweight andobesity in relation to the more rapid growth of total knee arthroplasty volume compared with total hip arthroplasty volume | 2014 | Risk not calculated |
| N. N. Dhalwani, F. Zaccardi, M. J. Davies and K. Khunti | Body mass index and mortality in people with and without diabetes: A UK Biobank study | 2018 | Risk not calculated |
| M. Ding, Y. Hu, J. Schwartz, W. P. Koh, J. M. Yuan, H. D. Sesso, J. Ma, J. Chavarro, F. B. Hu and A. Pan | Delineation of body mass index trajectory predicting lowest risk of mortality in U.S. men using generalized additive mixed model | 2016 | Risk not calculated |
| A. L. Feldman, S. J. Griffin, A. L. Ahern, G. H. Long, L. Weinehall, E. Fhärm, M. Norberg and P. Wennberg | Impact of weight maintenance and loss on diabetes risk and burden: a population-based study in 33,184 participants | 2017 | Risk not calculated |
| I. Figliuzzi, V. Presta, B. Citoni, F. Miceli, F. Simonelli, A. Battistoni, R. Coluccia, A. Ferrucci, M. Volpe and G. Tocci | Achievement of multiple therapeutic targets for cardiovascular disease prevention: Retrospective analysis of real practice in Italy | 2018 | Risk not calculated |
| V. L. Gordon-Dseagu, N. Shelton and J. Mindell | Diabetes mellitus and mortality from all-causes, cancer, cardiovascular and respiratory disease: evidence from the Health Survey for England and Scottish Health Survey cohorts | 2014 | Risk not calculated |
| S. A. Grover, M. Kaouache, P. Rempel, L. Joseph, M. Dawes, D. C. W. Lau and I. Lowensteyn | Years of life lost and healthy life-years lost from diabetes and cardiovascular disease in overweight and obese people: A modelling study | 2015 | Risk not calculated |
| F. J. Ha, H. C. Han, P. Sanders, K. Fendel, A. W. Teh, J. M. Kalman, D. O'Donnell, T. Leong, O. Farouque and H. S. Lim | Sudden cardiac death in the young: Incidence, trends, and risk factors in a nationwide study | 2020 | Population |
| A. Heltberg, J. S. Andersen, H. Sandholdt, V. Siersma, J. Kragstrup and C. Ellervik | Predictors of undiagnosed prevalent type 2 diabetes – The Danish General Suburban Population Study | 2018 | Risk not calculated |
| A. Hirayama, T. Goto, Y. J. Shimada, M. K. Faridi, C. A. Camargo and K. Hasegawa | Association of obesity with severity of heart failure exacerbation: A population-based study | 2018 | Confounding variables |
| L. L. N. Husemoen, L. S. Mørch, P. K. Christensen, N. V. Hartvig and M. D. Feher | All-cause and cardiovascular mortality among insulin-naïve people with type 2 diabetes treated with insulin Detemir or Glargine: A Cohort Study in the UK | 2021 | Obesity not defined or not defined by BMI |
| V. R. Kakarla, K. Nandipati, M. Lalla, A. Castro and S. Merola | Are laparoscopic bariatric procedures safe in superobese (BMI <50 kg/m2) patients? An NSQIP data analysis | 2011 | Risk not calculated |
| C. Koebnick, N. Smith, K. Huang, M. P. Martinez, H. A. Clancy and L. H. Kushi | The prevalence of obesity and obesity-related health conditions in a large, multiethnic cohort of young adults in California | 2012 | Confounding variables |
| R. Køster-Rasmussen, M. K. Simonsen, V. Siersma, J. E. Henriksen, B. L. Heitmann and N. De Fine Olivarius | Intentional weight loss and longevity in overweight patients with type 2 diabetes: A population-based cohort study | 2016 | Obesity not defined or not defined by BMI |
| E. R. C. Millett, S. A. E. Peters and M. Woodward | Sex differences in risk factors for myocardial infarction: Cohort study of UK Biobank participants | 2018 | Risk not calculated |
| C. L. Nelson, N. M. Elkassabany, A. F. Kamath and J. Liu | Low albumin levels, more than morbid obesity, are associated with complications after TKA | 2015 | Risk not calculated |
| R. W. Nicolay, R. S. Selley, M. A. Terry and V. K. Tjong | Body mass index as a risk factor for 30-day postoperative complications in knee, hip, and shoulder arthroscopy | 2019 | Risk not calculated |
| P. Nordström, N. L. Pedersen, Y. Gustafson, K. Michaëlsson and A. Nordström | Risks of myocardial infarction, death, and diabetes in identical twin pairs with different body mass indexes | 2016 | Obesity not defined or not defined by BMI |
| C. G. Owen, V. V. Kapetanakis, A. R. Rudnicka, A. K. Wathern, L. Lennon, O. Papacosta, D. G. Cook, S. G. Wannamethee and P. H. Whincup | Body mass index in early and middle adult life: prospective associations with myocardial infarction, stroke and diabetes over a 30-year period: the British Regional Heart Study | 2015 | Population |
| L. G. Persson, H. Lingfors, M. Nilsson and S. Mölstad | The possibility of lifestyle and biological risk markers to predict morbidity and mortality in a cohort of young men after 26 years follow-up | 2015 | Risk not calculated |
| K. Poulsen, B. Cleal, T. Clausen and L. L. Andersen | Work, diabetes and obesity: a seven year follow-up study among Danish health care workers | 2014 | Risk not calculated |
| J. S. Rana, G. H. Tabada, M. Solomon, J. C. Lo, M. Jaffe, S. H. Sung and A. S. Go | Levels of obesity and accuracy of the atherosclerotic cardiovascular risk equation in a large community-based cohort | 2017 | Abstract only |
| M. Schmidt, S. A. Johannesdottir, S. Lemeshow, T. L. Lash, S. P. Ulrichsen, H. E. Botker and H. T. Sorensen | Obesity in young men, and individual and combined risks of type 2 diabetes, cardiovascular morbidity and death before 55 years of age: A Danish 33-year follow-up study | 2013 | Confounding variables |
| C. Thornqvist, G. H. Gislason, L. Køber, P. F. Jensen, C. Torp-Pedersen and C. Andersson | Body mass index and risk of perioperative cardiovascular adverse events and mortality in 34,744 Danish patients undergoing hip or knee replacement | 2014 | Risk not calculated |
| Haase CL, Eriksen KT, Lopes S, Satylganova A, Schnecke V, McEwan P. | Body mass index and risk of obesity-related conditions in a cohort of 2.9 million people: Evidence from a UK primary care database. | 2020 | Confounding variables |

*BMI* body mass index

# Table S7. Included studies

| **Author** | **Title** | **Year** |
| --- | --- | --- |
| Ahlin et al. [7] | A new sensitive and accurate model to predict moderate to severe obstructive sleep apnea in patients with obesity | 2019 |
| Alssema et al. [8] | One risk assessment tool for cardiovascular disease, type 2 diabetes, and chronic kidney disease | 2012 |
| Alvi et al. [9] | The effect of BMI on 30 day outcomes following total joint arthroplasty | 2015 |
| Andersen et al. [10] | The obesity paradox in stroke: lower mortality and lower risk of readmission for recurrent stroke in obese stroke patients | 2015 |
| Batsis et al. [11] | Anthropometric measurements and survival in older Americans: results from the third National Health and Nutrition Examination Survey | 2014 |
| Bhaskaran et al. [12] | Association of BMI with overall and cause-specific mortality: a population-based cohort study of 3·6 million adults in the UK | 2018 |
| Blume et al. [13] | Variation in the risk of progression between glycemic stages across different levels of body mass index: evidence from a United States electronic health records system | 2015 |
| Bo et al. [14] | Prognostic implications for insulin-sensitive and insulin-resistant normal-weight and obese individuals from a population-based cohort | 2012 |
| Boggs et al. [15] | General and abdominal obesity and risk of death among black women | 2011 |
| Booth et al. [16] | Incidence of type 2 diabetes after bariatric surgery: population-based matched cohort study | 2014 |
| Borgeraas et al. [17] | Association of body mass index with risk of acute myocardial infarction and mortality in Norwegian male and female patients with suspected stable angina pectoris: a prospective cohort study | 2014 |
| Bruce [18] | The association between central fat distribution and recurrent cardiovascular disease events in female survivors of nonfatal myocardial infarction | 2015 |
| Burns et al. [19] | Body mass index and early outcomes following mitral valve surgery for degenerative disease | 2019 |
| Calori et al. [20] | Prevalence, metabolic features, and prognosis of metabolically healthy obese Italian individuals: the Cremona study | 2011 |
| Champagne-Langabeer et al. [21] | Obesity, treatment times, and cardiovascular outcomes after ST-elevation myocardial infarction: findings from Mission: Lifeline North Texas | 2017 |
| Chang et al. [22] | Impact of the National Health Service Health Check on cardiovascular disease risk: a difference-in-differences matching analysis | 2016 |
| Coles et al. [23] | Prediction of type 2 diabetes risk in people with non-diabetic hyperglycaemia: model derivation and validation using UK primary care data | 2020 |
| Crotti et al. [24] | Body mass index and mortality in elderly subjects from the Moli-sani Study: a possible mediation by low-grade inflammation? | 2018 |
| Czernichow et al. [25] | Body mass index, waist circumference and waist-hip ratio: which is the better discriminator of cardiovascular disease mortality risk?: evidence from an individual-participant meta-analysis of 82 864 participants from nine cohort studies | 2011 |
| Das et al. [26] | Impact of body weight and extreme obesity on the presentation, treatment, and in-hospital outcomes of 50,149 patients with ST-segment elevation myocardial infarction: results from the NCDR (National Cardiovascular Data Registry) | 2011 |
| de Boer et al. [27] | Overweight can be used as a tool to guide case-finding for cardiovascular risk assessment | 2015 |
| Ding et al. [28] | Risk factors of incident type 2-diabetes mellitus over a 3-year follow-up: results from a large Australian sample | 2015 |
| Edqvist et al.[29] | Contrasting associations of body mass index and hemoglobin A1c on the excess risk of acute myocardial infarction and heart failure in type 2 diabetes mellitus | 2019 |
| Eeg-Olofsson et al. [30] | The triglycerides-to-HDL-cholesterol ratio and cardiovascular disease risk in obese patients with type 2 diabetes: an observational study from the Swedish National Diabetes Register (NDR) | 2014 |
| Erridge et al. [31] | Obstructive sleep apnea in obese patients: a UK population analysis | 2021 |
| Ferket et al. [32] | Separate prediction of intracerebral hemorrhage and ischemic stroke | 2014 |
| Glogner et al. [33] | The association between BMI and hospitalization for heart failure in 83 021 persons with type 2 diabetes: a population-based study from the Swedish National Diabetes Registry | 2014 |
| Gray et al. [34] | Predicted 10-year risk of cardiovascular disease is influenced by the risk equation adopted: a cross-sectional analysis | 2014 |
| Gray et al. [5] | Different type 2 diabetes risk assessments predict dissimilar numbers at 'high risk': a retrospective analysis of diabetes risk-assessment tools | 2015 |
| Guasch Ferré et al. [35] | A risk score to predict type 2 diabetes mellitus in an elderly Spanish Mediterranean population at high cardiovascular risk | 2012 |
| Hoffman et al. [36] | The obesity paradox in spontaneous intracerebral hemorrhage: results from a retrospective analysis of the nationwide inpatient sample | 2020 |
| Hotchkiss et al. [37] | Adiposity has differing associations with incident coronary heart disease and mortality in the Scottish population: cross-sectional surveys with follow-up | 2013 |
| Jackson et al. [38] | Class I obesity is paradoxically associated with decreased risk of postoperative stroke after carotid endarterectomy | 2012 |
| Joshy et al. [39] | Body mass index and incident hospitalisation for cardiovascular disease in 158 546 participants from the 45 and Up Study | 2014 |
| Kjøllesdal et al. [40] | The association between obesity and cardiovascular disease mortality in different strata of socioeconomic position: evidence from pooled Norwegian health surveys | 2019 |
| Kjøllesdal et al. [41] | The association between BMI and mortality using early adulthood BMI as an instrumental variable for midlife BMI | 2018 |
| Ligthart et al. [42] | Lifetime risk of developing impaired glucose metabolism and eventual progression from prediabetes to type 2 diabetes: a prospective cohort study | 2016 |
| Ma et al. [43] | Body mass index in young adulthood and premature death: analyses of the US National Health Interview Survey linked mortality files | 2011 |
| Ma et al. [44] | Joint association of adiposity and smoking with mortality among U.S. adults | 2013 |
| Mathur et al. [45] | Quantifying the risk of type 2 diabetes in East London using the QDScore: a cross-sectional analysis | 2012 |
| McAuley et al. [46] | Fitness, fatness, and survival in adults with prediabetes | 2014 |
| Mørkedal et al. [47] | Risk of myocardial infarction and heart failure among metabolically healthy but obese individuals: HUNT (Nord-Trøndelag Health Study), Norway | 2014 |
| Mustafina et al. [48] | The risk of type 2 diabetes mellitus in a Russian population cohort according to data from the HAPIEE project | 2021 |
| Payvar et al. [49] | In-hospital outcomes of percutaneous coronary interventions in extremely obese and normal-weight patients: findings from the NCDR (National Cardiovascular Data Registry) | 2013 |
| Perotto et al. [50] | Obesity is associated with lower mortality risk in elderly diabetic subjects: the Casale Monferrato study | 2013 |
| Phillips et al. [51] | Comparison of diabetes risk score estimates and cardiometabolic risk profiles in a middle-aged Irish population | 2013 |
| Ratwatte et al. [52] | Relation of body mass index to outcomes in acute coronary syndrome | 2021 |
| Rauh et al. [53] | External validation of a tool predicting 7-year risk of developing cardiovascular disease, type 2 diabetes or chronic kidney disease | 2018 |
| Silventoinen et al. [54] | Weight status in young adulthood and survival after cardiovascular diseases and cancer | 2014 |
| Sing et al. [55] | Is obesity a risk factor for adverse events after knee arthroscopy? | 2016 |
| Skolarus et al. [56] | Association of body mass index and mortality after acute ischemic stroke | 2014 |
| Sundaram et al. [57] | An increased body mass index was not associated with higher rates of 30-day postoperative complications after unicompartmental knee arthroplasty | 2019 |
| Wilkinson et al. [58] | Development and validation of a model for predicting incident type 2 diabetes using quantitative clinical data and a Bayesian logistic model: a nationwide cohort and modeling study | 2020 |
| Winter et al. [59] | Obesity and abdominal fat markers in patients with a history of stroke and transient ischemic attacks | 2016 |
| Zatońska et al. [60] | Changes in diabetes prevalence and corresponding risk factors - findings from 3- and 6-year follow-up of PURE Poland cohort study | 2020 |
| Zhang et al. [61] | Nottingham knee osteoarthritis risk prediction models | 2011 |
| Iyen et al. [62] | Long-term body mass index changes in overweight and obese adults and the risk of heart failure, cardiovascular disease and mortality: a cohort study of over 260,000 adults in the UK | 2021 |
| Apold et al. [63] | Risk factors for knee replacement due to primary osteoarthritis, a population based, prospective cohort study of 315,495 individuals | 2014 |
| Hippisley-Cox et al. [64] | Derivation and validation of QStroke score for predicting risk of ischaemic stroke in primary care and comparison with other risk scores: a prospective open cohort study | 2013 |
| Costanzo et al. [65] | The obesity paradox in type 2 diabetes mellitus: relationship of body mass index to prognosis a cohort study | 2015 |

# Table S8. Quality assessment of the included studies using the adapted Newcastle Ottawa Scale

| **Author** | | **Selection**  **(max 8*)** | **Comparability ^a^**  **(max 2*)** | **Outcome**  **(max 3*)** | **Validation**  **(max 2*)** | **Total score**  **(max 15*)** |
| --- | --- | --- | --- | --- | --- | --- |
| **Risk equations** | |  |  |  |  |  |
| Ahlin et al. [7] | | ***** (5) | ** (2) | * (1) | * (1) | 9 |
| Alssema et al. [8] | | ***** (5) | ** (2) | *** (3) | * (1) | 11 |
| Booth et al. [16] | | ****** (6) | ** (2) | * (1) | (0) | 9 |
| Borgeraas et al. ^b^ [17] | | ****** (6) | ** (2) | ** (2) | (0) | 10 |
| Bruce [18] | | ***** (5) | ** (2) | ** (2) | (0) | 9 |
| Burns et al. [19] | | ****** (6) | ** (2) | * (1) | (0) | 9 |
| Chang et al. [22] | | ******* (7) | ** (2) | * (1) | ** (2) | 12 |
| Coles et al. [23] | | ******** (8) | ** (2) | ** (2) | * (1) | 13 |
| de Boer et al. [27] | | ****** (6) | ** (2) | * (1) | ** (2) | 11 |
| Ding et al. [28] | | ***** (5) | ** (2) | * (1) | (0) | 8 |
| Erridge et al. [31] | | ******* (7) | **(2) | *** (3) | (0) | 12 |
| Ferket et al. [32] | | ***** (5) | ** (2) | *** (3) | * (1) | 11 |
| Glogner et al. [33] | | ******** (8) | ** (2) | *** (3) | (0) | 13 |
| Gray et al. [34] | | ***** (5) | ** (2) | ** (2) | ** (2) | 11 |
| Gray et al. [5] | | ***** (5) | ** (2) | ** (2) | ** (2) | 11 |
| Guasch Ferré et al. [35] | | ******** (8) | ** (2) | ** (2) | * (1) | 13 |
| Jackson et al. [38] | | ****** (6) | ** (2) | *** (3) | (0) | 11 |
| Joshy et al. [39] | | ****** (6) | ** (2) | ** (2) | (0) | 10 |
| Ligthart et al. [42] | | ***** (5) | ** (2) | *** (3) | (0) | 10 |
| Mathur et al. [45] | | ******* (7) | ** (2) | ** (2) | ** (2) | 13 |
| Mustafina et al. [48] | | ******** (8) | ** (2) | ** (2) | * (1) | 13 |
| Phillips et al. [51] | | ******** (8) | ** (2) | *** (3) | * (1) | 14 |
| Rauh et al. [53] | | *** (3) | ** (2) | ** (2) | ** (2) | 9 |
| Wilkinson et al. [58] | | ******* (7) | ** (2) | *** (3) | * (1) | 13 |
| Winter et al. [59] | | ****** (6) | ** (2) | * (1) | (0) | 9 |
| Zhang et al. [61] | | **** (4) | ** (2) | *** (3) | * (1) | 10 |
| Apold et al. [63] | | ******** (8) | ** (2) | *** (3) | (0) | 13 |
| Hippisley-Cox et al. [64] | | ******** (8) | ** (2) | *** (3) | * (1) | 14 |
| Costanzo et al. [65] | | ***** (5) | ** (2) | ** (2) | (0) | 9 |
| **Risk of T2D** |  |  |  |  |  |  |
| Blume et al. [13] | | ******* (7) | ** (2) | * (1) | (0) | 10 |
| Ligthart et al. [42] | | ***** (5) | ** (2) | *** (3) | (0) | 10 |
| Zatońska et al. [60] | | ****** (6) | ** (2) | *** (3) | (0) | 11 |
| **Risk of CV event** |  |  |  |  |  |  |
| Edqvist et al. [29] | | ****** (6) | ** (2) | *** (3) | (0) | 11 |
| Iyen et al. ^b^ [62] | | ******** (8) | ** (2) | *** (3) | (0) | 13 |
| Mørkedal et al. [47] | | ****** (6) | ** (2) | *** (3) | (0) | 11 |
| **Risk of mortality (CV)** |  |  |  |  |  |  |
| Batsis et al. [11] | | ******* (7) | ** (2) | *** (3) | (0) | 12 |
| Bhaskaran et al. [12] | | ******* (7) | ** (2) | *** (3) | (0) | 12 |
| Boggs et al. [15] | | ***** (5) | ** (2) | *** (3) | (0) | 10 |
| Borgeraas et al. ^b^ [17] | | ****** (6) | ** (2) | ** (2) | (0) | 10 |
| Champagne-Langabeer et al. [21] | | ***** (5) | ** (2) | ** (2) | (0) | 9 |
| Crotti et al. [24] | | ******* (7) | ** (2) | *** (3) | (0) | 12 |
| Perotto et al. [50] | | ****** (6) | ** (2) | *** (3) | (0) | 11 |
| Ratwatte et al. [52] | | ***** (5) | ** (2) | ** (2) | (0) | 9 |
| Czernichow et al. [25] | | ******* (7) | ** (2) | *** (3) | * (1) | 13 |
| Das et al. [26] | | ******* (7) | ** (2) | *** (3) | ** (2) | 14 |
| Eeg-Olofsson et al. [30] | | ****** (6) | ** (2) | * (1) | (0) | 9 |
| Hotchkiss et al. [37] | | ****** (6) | ** (2) | *** (3) | (0) | 11 |
| Kjøllesdal et al. [40] | | ****** (6) | ** (2) | *** (3) | (0) | 11 |
| Kjøllesdal et al. [41] | | ****** (6) | ** (2) | *** (3) | (0) | 11 |
| Ma et al. [43] | | ****** (6) | ** (2) | *** (3) | (0) | 11 |
| Ma et al. [44] | | ****** (6) | ** (2) | *** (3) | (0) | 11 |
| McAuley et al. [46] | | ****** (6) | ** (2) | *** (3) | (0) | 11 |
| Payvar et al. [49] | | ****** (6) | ** (2) | * (1) | ** (2) | 11 |
| Iyen et al. ^b^ [62] | | ******** (8) | ** (2) | *** (3) | (0) | 13 |
| Silventoinen et al. ^b^ [54] | | ****** (6) | ** (2) | ** (2) | (0) | 10 |
| Bo et al. [14] | | ****** (6) | ** (2) | ** (2) | (0) | 10 |
| Calori et al. [20] | | ******* (7) | ** (2) | *** (3) | (0) | 12 |
| **Risk of mortality (TKA)** |  |  |  |  |  |  |
| Alvi et al. [9] | | ******** (8) | ** (2) | *** (3) | (0) | 13 |
| Sing et al. [55] | | ******** (8) | ** (2) | *** (3) | (0) | 13 |
| Sundaram et al. [57] | | ******** (8) | ** (2) | *** (3) | (0) | 13 |
| **Risk of mortality (stroke)** |  |  |  |  |  |  |
| Silventoinen et al. ^b^ [54] | | ****** (6) | ** (2) | ** (2) | (0) | 10 |
| Andersen et al. [10] | | ******** (8) | ** (2) | *** (3) | (0) | 13 |
| Hoffman et al. [36] | | ****** (6) | ** (2) | ** (2) | (0) | 10 |
| Skolarus et al. [56] | | ****** (6) | ** (2) | ** (2) | * (1) | 11 |
| BMI body mass index, CV cardiovascular, T2D type 2 diabetes, TKA total knee arthroplasty  ^a^ All studies were rated with the full number of stars, because the same risk equation and/or adjusted analysis (using the same variables) were used to compare the exposed (high BMI) and non-exposed (lower BMI) groups  ^b^ Studies in more than one category | | | | | | |

# References

1. van Staa TP, Gulliford M, Ng ES, Goldacre B, Smeeth L. Prediction of cardiovascular risk using Framingham, ASSIGN and QRISK2: how well do they predict individual rather than population risk? PLoS One. 2014;9(10):e106455.

2. Collins G, Mallett S, Omar O, Yu LM. Developing risk prediction models for type 2 diabetes: a systematic review of methodology and reporting. BMC Med. 2011;9(103):1–14.

3. Pate A, Emsley R, Ashcroft DM, Brown B, van Staa T. The uncertainty with using risk prediction models for individual decision making: an exemplar cohort study examining the prediction of cardiovascular disease in English primary care. BMC Med. 2019;17(1):134.

4. Page MJ, McKenzie JE, Bossuyt PM, Boutron I, Hoffmann TC, Mulrow CD, et al. The PRISMA 2020 statement: an updated guideline for reporting systematic reviews. BMJ. 2021;372:n71.

5. Gray BJ, Bracken RM, Turner D, Morgan K, Thomas M, Williams SP, et al. Different type 2 diabetes risk assessments predict dissimilar numbers at 'high risk': a retrospective analysis of diabetes risk-assessment tools. Br J Gen Pract. 2015;65(641):e852–60.

6. Wells G, Shea B, O'Connell D, Peterson J, Welch V, Losos M, et al. The Newcastle-Ottawa Scale (NOS) for assessing the quality of nonrandomised studies in meta-analyses. 2009. Available at: <http://www.ohri.ca/programs/clinical_epidemiology/oxford.asp>. Accessed June 30, 2021.

7. Ahlin S, Manco M, Panunzi S, Verrastro O, Giannetti G, Prete A, et al. A new sensitive and accurate model to predict moderate to severe obstructive sleep apnea in patients with obesity. Medicine (Baltimore). 2019;98(32):e16687.

8. Alssema M, Newson R, Bakker S, Stehouwer C, Heymans M, Nijpels G, et al. One risk assessment tool for cardiovascular disease, type 2 diabetes, and chronic kidney disease. Diabetes Care. 2012;35(4):741–8.

9. Alvi HM, Mednick RE, Krishnan V, Kwasny MJ, Beal MD, Manning DW. The effect of BMI on 30 day outcomes following total joint arthroplasty. J Arthroplasty. 2015;30(7):1113–7.

10. Andersen KK, Olsen TS. The obesity paradox in stroke: lower mortality and lower risk of readmission for recurrent stroke in obese stroke patients. Int J Stroke. 2015;10(1):99–104.

11. Batsis JA, Singh S, Lopez-Jimenez F. Anthropometric measurements and survival in older Americans: results from the third National Health and Nutrition Examination Survey. J Nutr Health Aging. 2014;18(2):123–30.

12. Bhaskaran K, dos-Santos-Silva I, Leon DA, Douglas IJ, Smeeth L. Association of BMI with overall and cause-specific mortality: a population-based cohort study of 3·6 million adults in the UK. Lancet Diabetes Endocrinol. 2018;6(12):944–53.

13. Blume SW, Li Q, Huang JC, Hammer M, Graf TR. Variation in the risk of progression between glycemic stages across different levels of body mass index: evidence from a United States electronic health records system. Curr Med Res Opin. 2015;31(1):115–24.

14. Bo S, Musso G, Gambino R, Villois P, Gentile L, Durazzo M, et al. Prognostic implications for insulin-sensitive and insulin-resistant normal-weight and obese individuals from a population-based cohort. Am J Clin Nutr. 2012;96(5):962–9.

15. Boggs DA, Rosenberg L, Cozier YC, Wise LA, Coogan PF, Ruiz-Narvaez EA, et al. General and abdominal obesity and risk of death among black women. N Engl J Med. 2011;365(10):901–8.

16. Booth H, Khan O, Prevost T, Reddy M, Dregan A, Charlton J, et al. Incidence of type 2 diabetes after bariatric surgery: population-based matched cohort study. Lancet Diabetes Endocrinol. 2014;2(12):963–8.

17. Borgeraas H, Hertel JK, Svingen GFT, Seifert R, Pedersen EKR, Schartum-Hansen H, et al. Association of body mass index with risk of acute myocardial infarction and mortality in Norwegian male and female patients with suspected stable angina pectoris: a prospective cohort study. BMC Cardiovasc Disord. 2014;14(68).

18. Bruce SA. The association between central fat distribution and recurrent cardiovascular disease events in female survivors of nonfatal myocardial infarction. J Cardiovasc Nurs. 2015;30(2):E15–22.

19. Burns DJP, Rapetto F, Angelini GD, Benedetto U, Caputo M, Ciulli F, et al. Body mass index and early outcomes following mitral valve surgery for degenerative disease. J Thorac Cardiovasc Surg. 2021;161(5):1765–73.e2.

20. Calori G, Lattuada G, Piemonti L, Garancini MP, Ragogna F, Villa M, et al. Prevalence, metabolic features, and prognosis of metabolically healthy obese Italian individuals: the Cremona study. Diabetes Care. 2011;34(1):210–5.

21. Champagne-Langabeer T, Kim J, Bower JK, Gardner A, Fowler R, Langabeer JR, 2nd. Obesity, treatment times, and cardiovascular outcomes after ST-elevation myocardial infarction: findings from Mission: Lifeline North Texas. J Am Heart Assoc. 2017;6(9):e005827.

22. Chang KCM, Lee JT, Vamos EP, Soljak M, Johnston D, Khunti K, et al. Impact of the National Health Service Health Check on cardiovascular disease risk: a difference-in-differences matching analysis. CMAJ. 2016;188(10):E228–38.

23. Coles B, Khunti K, Booth S, Zaccardi F, Davies MJ, Gray LJ. Prediction of type 2 diabetes risk in people with non-diabetic hyperglycaemia: model derivation and validation using UK primary care data. BMJ Open. 2020;10(10):e037937.

24. Crotti G, Gianfagna F, Bonaccio M, Di Castelnuovo A, Costanzo S, Persichillo M, et al. Body mass index and mortality in elderly subjects from the Moli-sani Study: a possible mediation by low-grade inflammation? Immunol Invest. 2018;47(8):774–89.

25. Czernichow S, Kengne AP, Stamatakis E, Hamer M, Batty GD. Body mass index, waist circumference and waist-hip ratio: which is the better discriminator of cardiovascular disease mortality risk?: evidence from an individual-participant meta-analysis of 82 864 participants from nine cohort studies. Obes Rev. 2011;12(9):680–7.

26. Das SR, Alexander KP, Chen AY, Powell-Wiley TM, Diercks DB, Peterson ED, et al. Impact of body weight and extreme obesity on the presentation, treatment, and in-hospital outcomes of 50,149 patients with ST-segment elevation myocardial infarction: results from the NCDR (National Cardiovascular Data Registry). J Am Coll Cardiol. 2011;58(25):2642–50.

27. de Boer AW, de Mutsert R, den Heijer M, Jukema JW, Rosendaal FR, Blom JW, et al. Overweight can be used as a tool to guide case-finding for cardiovascular risk assessment. Fam Pract. 2015;32(6):646–51.

28. Ding D, Chong S, Jalaludin B, Comino E, Bauman AE. Risk factors of incident type 2-diabetes mellitus over a 3-year follow-up: results from a large Australian sample. Diabetes Res Clin Pract. 2015;108(2):306–15.

29. Edqvist J, Rawshani A, Adiels M, Björck L, Lind M, Svensson AM, et al. Contrasting associations of body mass index and hemoglobin A1c on the excess risk of acute myocardial infarction and heart failure in type 2 diabetes mellitus. J Am Heart Assoc. 2019;8(24):e013871.

30. Eeg-Olofsson K, Gudbjörnsdottir S, Eliasson B, Zethelius B, Cederholm J, NDR. The triglycerides-to-HDL-cholesterol ratio and cardiovascular disease risk in obese patients with type 2 diabetes: an observational study from the Swedish National Diabetes Register (NDR). Diabetes Res Clin Pract. 2014;106(1):136–44.

31. Erridge S, Moussa O, McIntyre C, Hariri A, Tolley N, Kotecha B, et al. Obstructive sleep apnea in obese patients: a UK population analysis. Obes Surg. 2021;31(5):1986–93.

32. Ferket BS, Van Kempen BJH, Wieberdink RG, Steyerberg EW, Koudstaal PJ, Hofman A, et al. Separate prediction of intracerebral hemorrhage and ischemic stroke. Neurology. 2014;82(20):1804–12.

33. Glogner S, Rosengren A, Olsson M, Gudbjörnsdottir S, Svensson AM, Lind M. The association between BMI and hospitalization for heart failure in 83,021 persons with type 2 diabetes: a population-based study from the Swedish National Diabetes Registry. Diabet Med. 2014;31(5):586–94.

34. Gray BJ, Bracken RM, Turner D, Morgan K, Mellalieu SD, Thomas M, et al. Predicted 10-year risk of cardiovascular disease is influenced by the risk equation adopted: a cross-sectional analysis. Br J Gen Pract. 2014;64(627):e634–40.

35. Guasch-Ferre M, Bullo M, Costa B, Martinez-Gonzalez MA, Ibarrola-Jurado N, Estruch R, et al. A risk score to predict type 2 diabetes mellitus in an elderly Spanish Mediterranean population at high cardiovascular risk. PLoS One. 2012;7(3):e33437.

36. Hoffman H, Jalal MS, Furst T, Chin LS. The obesity paradox in spontaneous intracerebral hemorrhage: results from a retrospective analysis of the nationwide inpatient sample. Neurocrit Care. 2020;32(3):765–74.

37. Hotchkiss JW, Davies CA, Leyland AH. Adiposity has differing associations with incident coronary heart disease and mortality in the Scottish population: cross-sectional surveys with follow-up. Int J Obes (Lond). 2013;37(5):732–9.

38. Jackson RS, Black JH, 3rd, Lum YW, Schneider EB, Freischlag JA, Perler BA, et al. Class I obesity is paradoxically associated with decreased risk of postoperative stroke after carotid endarterectomy. J Vasc Surg. 2012;55(5):1306–12.

39. Joshy G, Korda RJ, Attia J, Liu B, Bauman AE, Banks E. Body mass index and incident hospitalisation for cardiovascular disease in 158 546 participants from the 45 and Up Study. Int J Obes (Lond). 2014;38(6):848–56.

40. Kjøllesdal M, Degerud E, Næss O. The association between obesity and cardiovascular disease mortality in different strata of socioeconomic position: evidence from pooled Norwegian health surveys. Eur J Public Health. 2019;29(6):1160–6.

41. Kjøllesdal MKR, Smith GD, Ariansen I, Kinge JM, Degerud E, Næss O. The association between BMI and mortality using early adulthood BMI as an instrumental variable for midlife BMI. Sci Rep. 2018;8(1):11499.

42. Ligthart S, van Herpt TTW, Leening MJG, Kavousi M, Hofman A, Stricker BHC, et al. Lifetime risk of developing impaired glucose metabolism and eventual progression from prediabetes to type 2 diabetes: a prospective cohort study. Lancet Diabetes Endocrinol. 2016;4(1):44–51.

43. Ma J, Flanders WD, Ward EM, Jemal A. Body mass index in young adulthood and premature death: analyses of the US National Health Interview Survey linked mortality files. Am J Epidemiol. 2011;174(8):934–44.

44. Ma J, Jemal A, Flanders WD, Ward EM. Joint association of adiposity and smoking with mortality among U.S. adults. Prev Med. 2013;56(3-4):178–84.

45. Mathur R, Noble D, Smith D, Greenhalgh T, Robson J. Quantifying the risk of type 2 diabetes in East London using the QDScore: a cross-sectional analysis. Br J Gen Pract. 2012;62(603):e663–70.

46. McAuley PA, Artero EG, Sui X, Lavie CJ, Almeida MJ, Blair SN. Fitness, fatness, and survival in adults with prediabetes. Diabetes Care. 2014;37(2):529–36.

47. Mørkedal B, Vatten LJ, Romundstad PR, Laugsand LE, Janszky I. Risk of myocardial infarction and heart failure among metabolically healthy but obese individuals: HUNT (Nord-Trondelag Health Study), Norway. J Am Coll Cardiol. 2014;63(11):1071–8.

48. Mustafina SV, Rymar OD, Shcherbakova LV, Verevkin EG, Pikhart H, Sazonova OV, et al. The risk of type 2 diabetes mellitus in a Russian population cohort according to data from the HAPIEE project. J Pers Med. 2021;11(2):119.

49. Payvar S, Kim S, Rao SV, Krone R, Neely M, Paladugu N, et al. In-hospital outcomes of percutaneous coronary interventions in extremely obese and normal-weight patients: findings from the NCDR (National Cardiovascular Data Registry). J Am Coll Cardiol. 2013;62(8):692–6.

50. Perotto M, Panero F, Gruden G, Fornengo P, Lorenzati B, Barutta F, et al. Obesity is associated with lower mortality risk in elderly diabetic subjects: the Casale Monferrato study. Acta Diabetol. 2013;50(4):563–8.

51. Phillips CM, Kearney PM, McCarthy VJ, Harrington JM, Fitzgerald AP, Perry IJ. Comparison of diabetes risk score estimates and cardiometabolic risk profiles in a middle-aged Irish population. PLoS One. 2013;8(11):e78950.

52. Ratwatte S, Hyun K, D'Souza M, Barraclough J, Chew DP, Shetty P, et al. Relation of body mass index to outcomes in acute coronary syndrome. Am J Cardiol. 2021;138:11–9.

53. Rauh SP, Rutters F, van der Heijden A, Luimes T, Alssema M, Heymans MW, et al. External validation of a tool predicting 7-year risk of developing cardiovascular disease, type 2 diabetes or chronic kidney disease. J Gen Intern Med. 2018;33(2):182–8.

54. Silventoinen K, Tynelius P, Rasmussen F. Weight status in young adulthood and survival after cardiovascular diseases and cancer. Int J Epidemiol. 2014;43(4):1197–204.

55. Sing DC, Luan TF, Feeley BT, Zhang AL. Is obesity a risk factor for adverse events after knee arthroscopy? Arthroscopy. 2016;32(7):1346–53.e1.

56. Skolarus LE, Sanchez BN, Levine DA, Baek J, Kerber KA, Morgenstern LB, et al. Association of body mass index and mortality after acute ischemic stroke. Circ Cardiovasc Qual Outcomes. 2014;7(1):64–9.

57. Sundaram K, Warren J, Anis H, George J, Murray T, Higuera CA, et al. An increased body mass index was not associated with higher rates of 30-day postoperative complications after unicompartmental knee arthroplasty. Knee. 2019;26(3):720–8.

58. Wilkinson L, Yi N, Mehta T, Judd S, Garvey WT. Development and validation of a model for predicting incident type 2 diabetes using quantitative clinical data and a Bayesian logistic model: a nationwide cohort and modeling study. PLoS Med. 2020;17(8):e1003232.

59. Winter Y, Pieper L, Klotsche J, Riedel O, Wittchen HU. Obesity and abdominal fat markers in patients with a history of stroke and transient ischemic attacks. J Stroke Cerebrovasc Dis. 2016;25(5):1141–7.

60. Zatońska K, Basiak-Rasała A, Różańska D, Karczewski M, Wołyniec M, Szuba A, et al. Changes in diabetes prevalence and corresponding risk factors - findings from 3- and 6-year follow-up of PURE Poland cohort study. BMC Public Health. 2020;20(1):843.

61. Zhang W, McWilliams DF, Ingham SL, Doherty SA, Muthuri S, Muir KR, et al. Nottingham knee osteoarthritis risk prediction models. Ann Rheum Dis. 2011;70(9):1599–604.

62. Iyen B, Weng S, Vinogradova Y, Akyea RK, Qureshi N, Kai J. Long-term body mass index changes in overweight and obese adults and the risk of heart failure, cardiovascular disease and mortality: a cohort study of over 260,000 adults in the UK. BMC Public Health. 2021;21(1):576.

63. Apold H, Meyer H, Nordsletten L, Furnes O, Baste V, Flugsrud GB. Risk factors for knee replacement due to primary osteoarthritis, a population based, prospective cohort study of 315,495 individuals. BMC Musculoskelet Disord. 2014;15(217).

64. Hippisley-Cox J, Coupland C, Brindle P. Derivation and validation of QStroke score for predicting risk of ischaemic stroke in primary care and comparison with other risk scores: a prospective open cohort study. BMJ. 2013;346:f2573.

65. Costanzo P, Cleland JG, Pellicori P, Clark AL, Hepburn D, Kilpatrick ES, et al. The obesity paradox in type 2 diabetes mellitus: relationship of body mass index to prognosis: a cohort study. Ann Intern Med. 2015;162(9):610–8.
